# A feasibility study with embedded pilot randomised controlled trial and process evaluation of electronic cigarettes for smoking cessation in patients with periodontitis

**DOI:** 10.1186/s40814-019-0451-4

**Published:** 2019-06-04

**Authors:** Richard Holliday, Philip M. Preshaw, Vicky Ryan, Falko F. Sniehotta, Suzanne McDonald, Linda Bauld, Elaine McColl

**Affiliations:** 1 Centre for Oral Health Research, School of Dental Sciences, Newcastle University, Framlington Place, Newcastle upon Tyne, NE2 4BW UK; 2 National University Centre for Oral Health, National University of Singapore, Singapore, Singapore; 3 Institute of Health & Society, Newcastle University, Newcastle upon Tyne, NE2 4AX UK; 4 Centre for Clinical Research, The University of Queensland, Building 71/918, Royal Brisbane and Women’s Hospital Campus, Herston, Queensland 4029 Australia; 5 Usher Institute of Population Health Sciences and Informatics, University of Edinburgh, Edinburgh, EH8 9AG UK

**Keywords:** Electronic cigarettes, Smoking, Tobacco, Periodontitis, Dental, Oral health, Cessation

## Abstract

**Background:**

Tobacco smoking is a major risk factor for several oral diseases, including periodontitis, and electronic cigarettes (e-cigarettes) are increasingly being used for smoking cessation. This study aimed to assess the viability of delivering and evaluating an e-cigarette intervention for smoking cessation within the dental setting, prior to a definitive study.

**Methods:**

A feasibility study, comprising a pilot randomised controlled trial and qualitative process evaluation, was conducted over 22 months in the Newcastle upon Tyne Hospitals NHS Dental Clinical Research Facility, UK. The pilot trial comprised a two-armed, parallel group, individually randomised, controlled trial, with 1:1 allocation. Participant eligibility criteria included being a tobacco smoker, having periodontitis and not currently using an e-cigarette. All participants received standard non-surgical periodontal therapies and brief smoking cessation advice. The intervention group additionally received an e-cigarette starter kit with brief training. Proposed outcomes for a future definitive trial, in terms of smoking behaviour and periodontal/oral health, were collected over 6 months to assess data yield and quality and estimates of parameters. Analyses were descriptive, with 95% confidence intervals presented, where appropriate.

**Results:**

Eighty participants were successfully recruited from a range of dental settings. Participant retention was 73% (*n* = 58; 95% CI 62–81%) at 6 months. The e-cigarette intervention was well received, with usage rates of 90% (*n* = 36; 95% CI 77–96%) at quit date. Twenty percent (*n* = 8; 95% CI 11–35%) of participants in the control group used an e-cigarette at some point during the study (against advice). The majority of the outcome measures were successfully collected, apart from a weekly smoking questionnaire (only 30% of participants achieved ≥ 80% completion). Reductions in expired air carbon monoxide over 6 months of 6 ppm (95% CI 1–10 ppm) and 12 ppm (95% CI 8–16 ppm) were observed in the control and intervention groups, respectively. Rates of abstinence (carbon monoxide-verified continuous abstinence for 6 months) for the two groups were 5% (*n* = 2; 95% CI 1–17%; control group) and 15% (*n* = 6; 95% CI 7–29%; intervention group).

**Conclusions:**

Data suggest that a definitive trial is feasible and that the intervention may improve smoking quit rates. Insights were gained into how best to conduct the definitive trial and estimates of parameters to inform design were obtained.

**Trial registration:**

ISRCTN, ISRCTN17731903; registered 19 September 2016 http://www.isrctn.com/ISRCTN17731903.

**Electronic supplementary material:**

The online version of this article (10.1186/s40814-019-0451-4) contains supplementary material, which is available to authorized users.

## Background

Periodontal diseases are amongst the most common inflammatory conditions in humans [1]. Periodontitis, an advanced form of periodontal disease, has a multifactorial aetiology, but the principal process involves a dental plaque biofilm accumulating in the subgingival environment, causing an immune and inflammatory response that leads to destruction of the tooth supporting structures. Consequences of periodontal disease progression include tooth mobility and eventually tooth loss. Severe periodontitis, threatening tooth retention, affects approximately 10% of UK adults, with moderate periodontitis affecting 40–60% [2]. A recent estimate is that 4.4 million adults in the UK may have severe disease [3].

There are multiple risk factors for periodontal diseases, but tobacco smoking is one of the most important [4]. Smoking is thought to affect the periodontal tissues via multiple pathways, including effects on the host immune and inflammatory response, impaired blood flow and microbiological changes [5]. Of particular relevance in the management of periodontitis is the knowledge that smokers who quit are 30% more likely to see clinically significant improvements than individuals who continue to smoke [6]. Smoking cessation advice (SCA) is therefore a critical component of periodontal therapy, and usual care involves a brief advice intervention, e.g. the ‘3 A’s’ technique: Ask, Advise, Act [7, 8].

The recent development of electronic cigarettes (e-cigarettes) has introduced a new option for smokers who wish to attempt quitting. Although still limited, there is a growing body of evidence to suggest that e-cigarettes are a useful smoking cessation aid [9]. The risks associated with using e-cigarettes appear to be much lower than those of tobacco cigarettes with several government and professional bodies estimating the health risks to be less than 5% of those associated with tobacco smoking [10, 11]. At a population level, e-cigarettes have been popular with 2.8 million users in the UK as of 2017 [12].

Research is required to determine the effectiveness of e-cigarettes as a smoking cessation or reduction tool within the dental setting and any subsequent impacts on oral health, specifically with regard to the periodontal tissues and periodontal treatment outcomes. An essential pre-requisite to a definitive trial of the effectiveness and cost-effectiveness of e-cigarettes in this context is a well-designed pilot trial to reduce the uncertainties inherent in the introduction and evaluation of a new technology. The aims of this feasibility study and pilot trial were to assess eligibility, recruitment and retention rates; to explore the feasibility and acceptability of the intervention and of trial procedures; and to collect data to inform power calculations for the definitive trial.

## Methods

### Study design and setting

This feasibility study included a single-centre, two-arm, parallel group, individually randomised controlled pilot trial (pilot RCT), with 1:1 allocation, conducted in a dental clinical research facility (DCRF), located in the Newcastle Dental Hospital (NDH), part of the Newcastle upon Tyne Hospitals NHS Foundation Trust, Newcastle upon Tyne, UK. The participants were smokers who had a diagnosis of periodontitis and who were provided with a smoking cessation intervention alongside their standard periodontal therapy. Participants in the control group received usual care (SCA), and those in the intervention group received usual care plus an offer of an e-cigarette starter kit.

A favourable ethical opinion was obtained from the North East-Tyne & Wear South NHS Research Ethics Committee (16/NE/0219), and the trial was prospectively registered (ISRCTN17731903). The study adhered to the Consolidating Standards of Reporting Trials (CONSORT) guidance for pilot and feasibility trials [13] (see Additional file 1 for the completed CONSORT checklist). Protocol amendments with reasons are detailed in Additional file 2.

### Identification and recruitment

Potential participants were identified through two routes: at new patient and treatment clinics of the NDH or by primary care practitioners (general dental practitioners, therapists, hygienists) working in the north east England region. Table 1 details the participant eligibility criteria. Potential participants were identified by a member of the existing clinical care team. Where possible (in the NDH), a member of the research team attended the clinic to discuss the study with the patient, provide the participant information sheet and arrange study appointments. If required, a screening visit was arranged in the DCRF to check for participant eligibility and answer any further questions. Primary care practitioners in participant identification centres (PICs) who identified potentially eligible patients were asked to provide the patient with a participant information sheet and refer them directly to the DCRF.

**Table 1:** Eligibility criteria

Inclusion criteria	
Aged over 18 years old	
Smoker of burnt tobacco (≥10 factory-made cigarettes/day or 7 g [0.25 oz]) loose tobacco/day or 14 hand-rolled cigarettes/day)	
Not currently using an e-cigarette, or not having used one for more than 2 days in the last 30 days	
Willing and able to come to the DCRF for the required study visits	
Having a minimum of 16 natural teeth (excluding third molars)	
Being diagnosed with periodontitis, having interproximal pocket probing depths (PPDs) of ≥ 5 mm at ≥ 8 sites	
Exclusion criteria	
Having used an e-cigarette for more than 2 days in the last 30 days	
Infectious or systemic diseases (myocardial infarction, cerebrovascular accident; phaeochromocytoma; uncontrolled hyperthyroidism; liver or kidney problems; chronic obstructive pulmonary disease) that may be unduly affected by participation in this study	
Haemodynamically unstable patients hospitalised with severe arrhythmias	
Patients taking the medication adenosine (due to drug interaction risk)	
Lack of capacity to be able to consent to the research project and/or inability to follow study instructions	
Participation in a dental research study within the previous 20 days	
Pregnant by medical history, or nursing	
Received any non-surgical periodontal therapy other than a routine scale and polish in the last 6 months	
Currently undergoing or requiring extensive dental, orthodontic or implant treatment, or treatment for peri-implantitis	
Clinical characteristics requiring further discussion with potential participants	
Asthma (severity needed to be assessed, patient made aware that nicotine replacement therapy (NRT) is better than smoking but best to use NRT as a short-term stop smoking treatment)	
Long-term throat disease (severity needs to be assessed, NRT use may exacerbate symptoms)	
Stomach ulcer, duodenal ulcer, irritation or inflammation of the stomach or throat (NRT may exacerbate symptoms)	
Diabetes mellitus (advised to monitor their blood glucose more closely when initiating treatment, advised to discuss this with their doctor or diabetic nurse specialist)	
Those taking theophylline, clozapine and ropinirole medications (metabolised by CYP 1A2 and with a narrow therapeutic window, can be affected by stopping smoking, advised to see their doctor to discuss changing the dose prior to starting the quit attempt).	

### Randomisation

Following assessment of eligibility and completion of written informed consent, participants were randomised to the control or intervention group, in a 1:1 ratio using random permuted blocks of variable size (2, 4 or 6). The allocation schedule was generated by a statistician with no other involvement in the study, and randomisation was performed using a secure password-protected web-based system. There were no stratification factors.

### Sample size

No formal sample size calculation was performed for this pilot RCT. In line with recommendations for pilot trials [14], our target was to achieve at least 30 patients retained in each arm of the trial. Based on prior experience, we anticipated an attrition rate of 25% and therefore aimed to randomise 40 participants to each arm of the study, 80 in total.

### Interventions

All participants in this study received SCA delivered by a single treating dentist (RH) alongside their dental and periodontal treatment, as part of usual care. This SCA followed the ‘3 A’s’ (Ask, Advise, Act) technique [8]. A referral to the ‘Newcastle Stop Smoking’ services was available to all participants. The SCA intervention was audio-recorded to allow tests for fidelity and is fully described in Additional file 3.

Participants in the intervention group were also offered an e-cigarette starter kit (as detailed in Additional file 4). The participants were provided with an approximately 2-week supply of e-liquid (with a choice of flavour and nicotine strength) and information on where to buy more. The e-cigarette intervention is fully described in Additional file 5.

All participants received standard non-surgical periodontal therapy. Oral hygiene instruction (detailed in Additional file 6) and full-mouth debridement were provided in line with local and international guidance on an individualised basis [15].

### Concomitant care

Participants in the control group were asked to commit to not using an e-cigarette for the duration of the study, especially during the first 4 weeks, and were invited to sign a commitment form agreeing to this. Participants in the intervention group were advised to use only the recommended brand of e-liquids for the duration of the study.

### Outcomes and data collection methods

Feasibility outcome measures included rates of eligibility, recruitment and retention; intervention acceptability; randomisation group cross-over rates; and the provision of data on smoking and oral health outcomes being rehearsed for the future definitive trial. The smoking behaviour outcomes comprised self-reported tobacco and e-cigarette use, expired air carbon monoxide (eCO), salivary cotinine (SC), salivary anabasine (SA), Fagerstrom Test for Nicotine Dependence (FTND) [16] and Mood and Physical Symptoms Scale (MPSS) [17]. Rates of continuous eCO-verified smoking abstinence at 6 months were calculated following the Russell Standard (RS6) [18]. The oral health outcomes comprised pocket probing depth (PPD), Modified Gingival Index (MGI) [19], plaque index (PI) [20], clinical attachment level (CAL), bleeding on probing (BOP), clinical oral dryness score (CODS) [21], periodontal epithelial surface area (PESA) [22], Periodontal Inflamed Surface Area (PISA) [22] and the UK Oral Health-related Quality of Life measure (OHQoL-UK) [23]. The data collection method for each outcome is described in Additional file 7. Adverse events were monitored at each study visit.

### Blinding

Due to the nature of this study, the participants and treating clinicians could not be blinded to the assigned intervention. However, the oral health indices were collected by a single, trained, calibrated research hygienist who was blinded to the assigned intervention. Participants were asked not to disclose their smoking status or methods of smoking cessation during the assessment appointments.

### Study visits and follow-up

Participants were asked to attend for six study visits over 6 months. There were no additional visits for research purposes with all visits being in line with normal periodontal therapy and follow-up. Randomisation, smoking cessation interventions and baseline measurements/sample collection were delivered at visit 1. Visit 1 was delivered in two parts. First, a dentist confirmed eligibility, obtained written consent, collected baseline smoking-related outcome measures and OHQoL-UK, randomised participants and provided the appropriate smoking cessation intervention. Second, participants were seen by a research hygienist, blinded to treatment allocation and smoking status, who collected the remaining clinical measurements. The initial periodontal treatment was delivered over visits 2 and 3. Visit 2 was designated as the target quit date and was arranged after discussion with the participant with the recommendation that it was ideally within 4 weeks of visit 1 (the actual duration varied between 0 and 9 weeks with a mean [SD] of 2.6 [2.0] weeks). Three follow-up visits with data collection and supportive periodontal therapy were conducted at 4 weeks (visit 4), 3 months (visit 5) and 6 months (visit 6) post-quit date (visit 2). A weekly smoking status online or paper questionnaire was distributed for the duration of the study, depending on participant preference. The online version was distributed weekly, and the paper version was distributed and completed at each study visit. A schedule of events is provided in Additional file 8.

### Collection and analysis of biological samples

Saliva was collected at each study visit (excluding visit 3). Gingival crevicular fluid (GCF) and subgingival plaque samples were collected prior to recording periodontal indices at visits 1, 5 and 6 (this pilot RCT will report on the ability to collect these samples rather than their analysis, which will be reported elsewhere).

### Data collection and analysis for the process evaluation

One-to-one semi-structured interviews were conducted with a purposive sample of 28 participants, 14 of whom received the e-cigarette intervention and the remainder of whom received the control condition (dentist-delivered SCA). Participants were sampled to reflect a range of ages and smoking behaviours. An initial interview was conducted shortly after the SCA intervention (usually at visit 3) with a follow-up interview approximately 6 months later (usually at visit 6). Interviews were audio-recorded, anonymised and transcribed verbatim. Transcripts were analysed thematically.

### Data analysis for the pilot RCT

Analyses were performed according to a predefined statistical analysis plan (Additional file 9). In accordance with recommendations for the analysis of pilot and feasibility studies, the data analyses were descriptive, and statistical comparisons between the randomised groups were not undertaken. For the feasibility outcome measures, all proportions/rates were reported with 95% confidence intervals (CIs). Quantitative outcome measures were reported as means and standard deviations with 95% CIs.

Participants with missing smoking outcome data (e.g. those not attending for review) were considered as continuing smokers or to have resumed smoking, in line with standard research practice [9, 18]. For continuous data (eCO, SC, SA), missing data were not imputed. Missing periodontal data due to participant loss to follow-up were not imputed. For teeth that were lost or extracted during the study period, a ‘last observation carried forward’ approach was used for the periodontal indices where possible, i.e. if a tooth was lost between visit 5 and 6, then the periodontal data were carried forwards from visit 5. If a tooth was lost before visit 5, then no data were imputed. For missing FTND and MPSS questionnaire data, the ‘rule of halves’ was employed [24–26]. For missing OHQoL-UK questionnaire data, patients who had not responded to ≥ 10% of the items in OHQoL-UK questionnaire were excluded from analyses, with their responses being treated as missing data. For patients who had < 10% missing responses, values for the missing items were derived using group mean score imputation for each item in order to calculate the individual domain scores and the summary scores as reported previously [27–29]. Data were analysed in SPSS (IBM SPSS Statistics, Version 24, Chicago, SPSS Inc.).

## Results

**Fig. 1:**
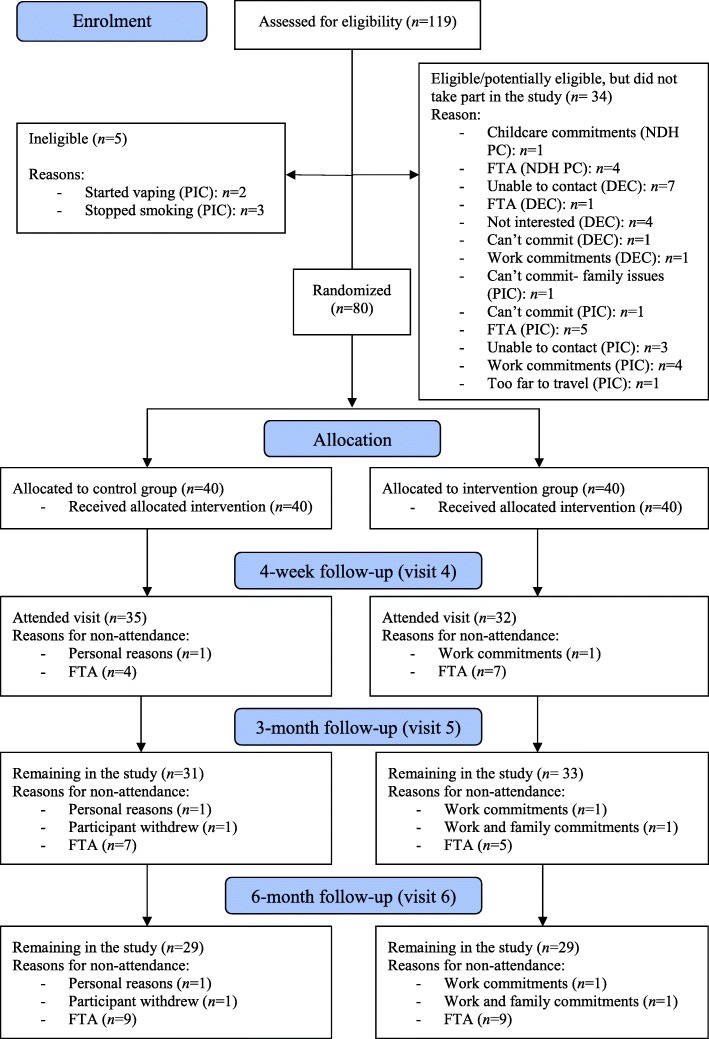
CONSORT flow diagram. NDH PC = Newcastle Dental Hospital (NDH) periodontal new patient clinic, DEC = dental emergency clinic (at NDH), PIC = participant identifying centre (primary care), FTA = failed to attend

One hundred and nineteen potentially eligible participants were identified over the 15-month recruitment period (20/09/2016–07/12/2017). Of these, 80 were found to be eligible and enrolled in the study. Participants were recruited from a range of sources (see Additional file 10). Screening data were only available for one recruitment source (NDH periodontal new patient clinic), from which the eligibility rate (number eligible/number screened) was estimated to be 7.4% (29/391, 95% CI 5.2–10.5%) [complete eligibility outcomes are presented in Additional file 11]. Data collection was completed on 7 June 2018, when the last patient visit occurred. Figure 1 shows the CONSORT flow diagram.

### Participant baseline characteristics

The study sample comprised 38 (47.5%) males and 42 (52.5%) females (Table 2). Ethnicity was primarily white (*n* = 75, 94%) (British, Irish or other white) with five (6%) Asian or Asian British, reflective of the North East’s population [30]. The majority of the participants were in employment (*n* = 60, 75%), mainly working in routine or manual (*n* = 20, 25%) or intermediate (*n* = 22, 27.5%) occupations. The mean age of the participants at randomisation was 44.3 years, ranging from 19 to 71 years. The mean number of cigarettes smoked per day was 17.4. The majority of participants reported started smoking in their teenage years, with a mean age of 16 years. The mean baseline eCO reading was 20.6 ppm. The participants had a moderate nicotine dependence with a mean FTND score at baseline of 5.0. The number of teeth (excluding third molars) that participants had at baseline ranged from 16 to 28, with a mean of 23.9. The participants demonstrated a severe level of periodontal disease, in keeping with the study inclusion criteria. The mean PPD was 4.0 mm with the mean percentage of sites with PPDs ≥ 5 mm being 40%. The mean percentage BOP was 20%. A low-moderate amount of xerostomia was observed with a mean CODS of 4.0, indicating some reduced mucosal wetness.

**Table 2:** Participant baseline characteristics

	Control group *n* = 40	Intervention group *n* = 40	Total *n* = 80
Sex, *n* (%)			
Female	20 (50%)	22 (55%)	42 (52.5%)
Male	20 (50%)	18 (45%)	38 (47.5%)
Ethnicity, *n* (%)			
White (British, Irish, other White)	36 (90%)	39 (97.5%)	75 (93.8%)
Asian or Asian British (Indian, Pakistani, Bangladeshi, other Asian)	4 (10%)	1 (2.5%)	5 (6.3%)
Occupation, *n* (%)			
Working in a routine or manual occupation	9 (22.5%)	11 (27.5%)	20 (25%)
Working in an intermediate occupation	9 (22.5%)	13 (32.5%)	22 (27.5%)
Working in a managerial or professional occupation	9 (22.5%)	9 (22.5%)	18 (22.5%)
Unemployed/not working for a year or more	6 (15%)	2 (5%)	8 (10%)
Full-time student	0	1 (2.5%)	1 (1.3%)
Retired	1 (2.5%)	4 (10%)	5 (6.3%)
Sick/disabled/unable to return to work	4 (10%)	0	4 (5.0%)
Home carer (unpaid)	2 (5%)	0	2 (2.5%)
Age (years), mean (SD)	44.6 (9.5)	44.0 (11.8)	44.3 (10.7)
Number of cigarettes/day (any), mean (SD)	17.5 (6.9)	17.4 (6.4)	17.4 (6.6)
Number of factory cigarettes/day, mean (SD)	16.6 (7.2), *n* = 33	14.8 (4.4), *n* = 26	15.8 (6.1), *n* = 59
Number of hand-rolled cigarettes/day, mean (SD)	19.0 (7.1), *n* = 8	22.1 (7.0), *n* = 14	21.0 (7.0), *n* = 22
Age started smoking, mean (SD)	16.0 (2.8)	15.3 (3.2)	15.7 (3.0)
eCO (ppm), mean (SD)	18.1 (10.0)	23.0 (12.2)	20.6 (11.3)
FTND, mean (SD)	5.0 (2.4)	5.0 (1.8)	5.0 (2.1)
MPSS, mean (SD)	22.8 (7.0)	22.8 (5.9)	22.8 (6.4)
SC (ng/ml), mean (SD)	303.4 (128.3)	342.6 (138.1)	323.5 (134.0)
SA (ng/ml), mean (SD)	1.1 (1.4)	1.2 (1.2)	1.2 (1.3)
Number of teeth (excluding 3rd molars), mean (SD)	24.0 (3.6)	23.8 (4.0)	23.9 (3.8)
Mean PI, mean (SD)	1.1 (0.7)	0.8 (0.6)	1.0 (0.7)
% BOP score, mean (SD)	23.9 (18.3)	16.5 (13.4)	20 (16.4)
Mean MGI, mean (SD)	2.5 (0.5)	2.5 (0.4)	2.5 (0.5)
Mean PPD (mm), mean (SD)	4.1 (0.8)	3.9 (0.7)	4.0 (0.7)
Mean CAL (mm), mean (SD)	5.2 (1.4)	5.1 (1.3)	5.1 (1.3)
PESA (mm^2^), mean (SD)	2134.1 (666.7)	2013.8 (644.4)	2073.9 (654.3)
PISA (mm^2^), mean (SD)	634.5 (629.9)	386.7 (346.2)	510.6 (520.2)
No. of sites with PPD ≥ 5 mm, mean (SD)	60.5 (30.9)	54.3 (27.5)	57.4 (29.2)
% of sites with PPD ≥ 5 mm, mean (SD)	42.2 (19.6)	38.4 (18.1)	40.3 (18.8)
No. of sites with PPD ≤ 4 mm, mean (SD)	83.3 (32.1)	87.1 (30.3)	85.2 (31.1)
% of sites with PPD ≤ 4 mm, mean (SD)	58.0 (19.4)	60.8 (19.1)	59.4 (19.2)
CODS, mean (SD)	4.0 (1.3)	4.1 (1.0)	4.0 (1.2)
OHQoL-UK, mean (SD)	42.7 (6.6)	43.6 (8.7)	43.1 (7.7)

*BOP* bleeding on probing, *CAL* clinical attachment level, *CODS* clinical oral dryness score, *eCO* expired air carbon monoxide, *FTND* Fagerstrom Test of Nicotine Dependence, *MGI* Modified Gingival Index, *MPSS* Mood and Physical Symptoms Scale, *NCSCT* National Centre for Smoking Cessation and Training, *OHQoL-UK* UK Oral Health-related Quality of Life measure, *PESA* periodontal epithelial surface area, *PI* plaque index, *PISA* Periodontal Inflamed Surface Area, *PPD* Pocket probing depth, *SA* salivary anabasine, *SC* salivary cotinine

There was good balance with respect to sex, age, ethnicity, occupation and smoking behaviour demographics across the groups (Table 2). Most of the oral health outcomes were approximately balanced across randomisation groups, although it was noted that percentage sites with PPD ≥ 5 mm, PI, BOP and PISA (which are associated with the severity of periodontitis) were slightly higher on average in the control group (Table 2).

### Participant follow-up

Four participants withdrew from the study, and 18 were lost to follow-up. The most frequent time point for withdrawal/loss to follow-up was after visit 3 (second and final treatment visit). There were no differences in the numbers of participants attending each visit by randomisation group and only minor differences in the proportion attending within the designated visit window (see Additional file 12). At the final data collection point (visit 6, 6 months), 11 participants in each randomisation group did not attend, giving 58 participants for the final analysis. Those participants lost to follow-up appeared to have higher eCO and FTND readings and more severe periodontal diseases at baseline (see Additional file 13).

### Safety data

There were no serious adverse events (SAEs). However, 56 adverse events (AEs) were reported in 35 participants. The most frequently reported AEs were toothache, dentine hypersensitivity, tooth/teeth loss, dental/periodontal abscess and fractured/carious filling or tooth (Table 3). Seven participants had unplanned tooth extractions during the study period, losing a total of 15 teeth between them. Two participants reported mouth ulceration, and three separate participants reported soreness of the intra-oral soft tissues. These five participants were in the intervention group, and the symptoms could be related to the e-cigarette intervention (other forms of orally administered NRT have been associated with soreness and ulceration) or could be the result of the higher quit rate in the intervention group (smoking cessation is also associated with soreness and ulceration).

**Table 3:** Summary of adverse events (AEs)

Adverse events	Control group		Intervention group	
	AEs (*n*)	Participants affected (*n*)	AEs (*n*)	Participants affected (*n*)
Toothache	4	4	11	9
Dentine hypersensitivity	3	3	3	3
Tooth/teeth loss	5 (6 teeth)	4	5 (9 teeth)	3
Dental/periodontal abscess	2	2	3	3
Mouth ulceration	0	0	2	2
Soreness of intra-oral soft tissues	0	0	3	3
Fractured/carious filling or tooth	3	3	2	2
Other	3	2	6	5

### Participant compliance

Participant compliance with attending review visits (visits 4, 5, 6) was similar across randomisation group, but differences were present across recruitment source (see Additional file 14). Participant compliance with completing the weekly smoking status questionnaire was poor, with only 46% of participants completing the questionnaire at least half of the time (> 14 entries) and 30% completing it at least 80% of the time (> 23 entries), increasing to 64% and 41% respectively, for those 58 participants who completed the study (see Additional file 15).

### Intervention usage

One participant in the intervention group declined the starter kit on the grounds that he did not intend to change his smoking behaviour (intention to quit was not an inclusion criterion for this study). Acceptability of the e-cigarette intervention was high, with 90% of participants using the device at the quit date (visit 2). The most popular initial e-liquid flavour combination was tobacco and mint followed by mint alone. Over half of participants (52%) did not include a tobacco flavour in their initial selection, while 62% included mint flavour (see Additional file 16). The most frequently selected nicotine strengths were 12 mg/ml and 18 mg/ml, selected by over half of participants, while none opted for the lowest concentration option (see Additional file 17). The proportion of participants still using the device remained high (> 70%) at both the 4-week (visit 4) and 3-month (visit 5) reviews. By the end of the study, approximately half of the participants in the intervention group were still using the e-cigarette, increasing to 72% for those 29 who completed the study (see Additional file 18). Based on self-reporting of those who attended all visits, the mean (SD) number of days using the e-cigarette in the intervention group was 196 (40.2).

Eleven participants used non-recommended e-liquid brands on at least one time point during the study (see Additional file 17). They were not obviously different from the other participants in the intervention group with regard to age, gender and baseline smoking behaviour. However, they made up a high proportion of the quitters, e.g. five of the six participants (83%) who achieved RS6. Six participants reported using a different e-cigarette device (i.e. not the one supplied in the starter kit) during the study (see Additional file 17).

### Process evaluation

Interviews with study participants found that a number of factors could influence both positive and negative attitudes towards e-cigarettes. In terms of positive attitudes, these were interactions with existing users (vapers), health benefits, a perception that vaping was seen as socially acceptable and the behavioural and sensory similarities to tobacco smoking. More negative attitudes were influenced by personal previous e-cigarette experiences, concerns about addiction, concerns relating to possible health risks of using e-cigarettes and lack of social acceptability. Interviews also explored the acceptability of the e-cigarette starter kit intervention. Overall, it was perceived to be acceptable with participants, and they were happy to source and purchase their own supplies of e-liquid after the initial period. Interviewees differed in their choice and experience of using different e-liquid flavours, and there were some instances of technical difficulties in using the device, particularly in the first few weeks. Participants have relatively dichotomous views on flavour preferences, for or against tobacco flavours. Additional file 19 provides further details of these findings with illustrative quotes.

### E-cigarette use in the control group

Two control participants declined to sign the commitment form, and one of these went on to use an e-cigarette during the study. In total, eight participants in the control group (20%, 95% CI 11–35%) reported using an e-cigarette at some point during the study (against instructions), with one reporting usage at all post-randomisation visits (see Additional file 20).

### Methods of smoking cessation (non-e-cigarette)

A variety of smoking cessation techniques were used by the participants (see Additional file 21). Forty percent of the control group reported that they made contact with the Newcastle Stop Smoking service, compared to none in the intervention group. Four of the eight participants (50%) who used an e-cigarette in the control group also reported making contact with the Newcastle Stop Smoking service.

### Biological samples

All the required biological samples were successfully collected at visit 1 (baseline) and visit 6 (6 months). Five percent of samples could not be collected at visit 5 (3 months) because there had been insufficient healing time following the periodontal therapy (see Additional file 22).

### Smoking and oral health outcome data

Overall, excellent data completeness was achieved across the outcome measures used in this study for those attending for follow-up visits. Most measures achieved 100% completeness at all time points (see Additional file 22). Overall, those in the intervention group appeared, descriptively, to have improved smoking cessation outcomes, although the 95% CIs were wide (see Table 4). Changes from baseline to 6 months for the oral health outcome measures were very similar in both groups (see Table 5). However, results from this pilot RCT should be interpreted cautiously given it was not designed to detect differences between groups and has not been analysed in a way that would account for any potential confounding factors [31, 32].

**Table 4:** Summary of smoking outcome measure data

Outcome	Control			Intervention		
	*n*	Baseline rate	Rate at follow-up (95% CI)	*n*	Baseline rate	Rate at follow-up (95% CI)
4-week quitter (eCO or SC/SA verified)	40	NA	5% (1 to 17%)	40	NA	28% (16 to 43%)
6-month quitter (RS6)	40	NA	5% (1 to 17%)	40	NA	15% (7 to 29%)
	*n*	Baseline mean (SD)	Mean change from baseline to 6 months (SD; 95% CI)	*n*	Baseline mean (SD)	Mean change from baseline to 6 months (SD; 95% CI)
eCO (ppm)	29	17.1 (10.4)	− 5.8 (12.3; − 10.5 to − 1.1)	29	22.0 (12.8)	− 12.0 (11.0; − 16.2 to − 7.9)
FTND	29	4.6 (2.5)	− 1.6 (2.1; − 2.4 to − 0.8)	29	4.6 (1.6)	− 1.9 (2.0; − 2.7 to − 1.2)
MPSS	29	22.8 (7.5)	− 2.8 (8.3; − 6.0 to 0.3)	29	21.8 (4.9)	− 2.8 (8.8; − 6.1 to 0.6)
SC (ng/ml)	29	277.2 (131.5)	− 37.1 (133.4; − 90.0 to 15.7)	29	326.3 (145.5)	− 62.2 (132.3; − 112.5 to − 11.8)
SA (ng/ml)	29	0.8 (0.8)	0.5 (2.3; − 0.5 to 1.4)	29	1.2 (1.3)	− 0.4 (1.2; − 0.9 to 0.0)

In line with recommendations for smoking cessation trials [4], participants with missing smoking outcome data (e.g. those not attending for review) were considered as continuing smokers or to have resumed smoking. Hence, the denominator for the 4-week and 6-month quitter outcome is the baseline number of participants (*n* = 40). For continuous variables, missing data were not imputed

*eCO* expired air carbon monoxide, *RS6* Russell standard 6-month quitter, *FTND* Fagerstroms test of nicotine dependence, *MPSS* Mood and Physical Symptoms Scale, *SC* salivary cotinine, *SA* salivary anabasine, *NA* not applicable

**Table 5:** Summary oral health outcome data

	Control (*n* = 29)			Intervention (*n* = 29)		
	*n*	Baseline mean (SD)	Mean change from baseline to 6 months (SD; 95% CI)	*n*	Baseline mean (SD)	Mean change from baseline to 6 months (SD; 95% CI)
Mean PPD (mm), mean (SD; 95% CI)	29	4.0 (0.8)	− 0.7 (0.5; − 0.9 to − 0.5)	29	3.8 (0.7)	− 0.8 (0.6; − 1.0 to − 0.6)
Percentage of sites with PPD ≥ 5 mm, mean (SD; 95% CI)	29	39.0 (20.8)	− 19.3 (13.0; − 24.2 to − 14.3)	29	35.1 (16.2)	− 21.5 (13.7; − 26.7 to − 16.3)
% BOP score, mean (SD; 95% CI)	29	25.2 (18.5)	− 11.1 (13.5; − 16.3 to − 6.0)	29	17.0 (12.6)	− 7.0 (13.6; (− 12.1 to − 1.8)
CODS, mean (SD; 95% CI)	29	3.7 (1.3)	− 0.7 (1.6; − 1.3 to − 0.1)	29	3.8 (0.9)	− 0.3 (1.3; − 0.8 to 0.2)
OHQoL-UK, mean (SD; 95% CI)	29	43.1 (6.7)	8.2 (15.1; 2.4 to 14.0)	29	43.4 (7.7)	9.6 (13.2; 4.6 to 14.6)

Missing periodontal data due to participant loss to follow-up were not imputed. For teeth that were lost or extracted during the study period, a ‘last observation carried forward’ approach was used for the periodontal indices where possible

*PPD* pocket probing depths, *BOP* bleeding on probing, *CODS* clinical oral dryness score, *OHQoL-UK* UK Oral Health-related Quality of Life measure

### Definitive study sample size calculation

There are two important outcomes of the e-cigarette intervention under investigation: smoking abstinence rates and oral/periodontal health. Therefore, we propose that the future definitive study should have co-primary outcomes and be powered accordingly.

In order to detect an 8% difference in 6-month smoking abstinence rates between intervention arms, with a control group rate of 7% (90% power, 5% significance level, two-sided test), 337 participants will be required per arm, 674 in total [33] (see Additional file 23 for further justifications and explanations). Given that any randomised participants who are lost to follow-up will be included in the intention to treat (ITT) analysis of smoking abstinence as smokers at 6 months, this sample size has not been adjusted for attrition. However, the consent rate amongst those eligible in the pilot RCT was 67% (95% CI 58–75%). Given that the definitive study will be conducted in multiple centres and the pilot RCT was conducted in a single centre, it would be prudent to use the lower bound of this 95% CI, and on this basis, the future definitive study would need to approach 1162 potentially eligible patients in order to consent and randomise 674. Sample sizes calculated for two potential oral/periodontal health outcome measures (mean PPD and percentage of sites with PPD ≥ 5 mm) are less than the sample size calculated for smoking abstinence (see Additional file 23 for complete calculations).

The sample size calculations were performed using the *proc power twosamplemeans and twosamplefreq* procedures in SAS version 9.4 of the SAS System for Windows 7, copyright© 2012 SAS Institute Inc.

## Discussion

### Main findings

This feasibility study with embedded pilot RCT successfully recruited 80 smokers with periodontitis of whom 58 completed the study, yielding a 73% retention rate, just below the expected 75% rate. Recruitment source affected several aspects with participants from PIC sites having the lowest conversion rates (from being identified to attending and consenting) and retention rates. The vast majority of the participants who did not finish the study failed to attend a visit and were then uncontactable, usually dropping out after the initial periodontal therapy (which was completed at visit 3). Future trial designs should consider whether the number and duration of the study visits can be reduced, and the setting of the research study changed (e.g. more visits in primary care), in order to minimise participant attrition.

The e-cigarette intervention was well received, with 90% of those in the intervention group using it at the quit date and over half using it for the duration of the study. Findings from the process evaluation also suggested that the e-cigarette intervention was acceptable to participants. Several participants chose to diverge from the recommended brand of e-liquid, and an interesting observation is that these individuals made up almost all of the quitters in the intervention group, potentially suggesting that they were particularly determined to quit smoking and engaged more broadly with the process to the extent of exploring the use of other e-liquids. Flexibility and choice may well be important determinants of quit success rates and should be considered when planning future trials. Furthermore, one in five participants in the control group used an e-cigarette, against instructions, an important variable to consider when designing future research, indicating the necessity of a pragmatic research design.

Outcome measures were successfully completed in clinic, but the weekly smoking questionnaire had poor completion rates. The pilot RCT results suggest that the e-cigarette intervention may have the potential to improve smoking cessation outcomes with a greater mean eCO reduction over the 6 months (control group: 6 ppm; intervention group: 12 ppm) and increased rates of eCO-verified continuous 6-month abstinence (control group: 5%; intervention group: 15%), although this needs confirming in an appropriately powered definitive trial. There was almost no difference on average in the change from baseline to 6 months in oral health outcome measures between the groups although the 95% CIs were wide due to the small sample sizes and relatively large between-participant variation in these measures.

The periodontal treatment was successfully delivered to all participants, usually over two visits. However, in a small number of participants (*n* = 3), due to clinical need or patient anxiety, the treatment was delivered over three or four visits. For three participants, we were unable to collect oral health indices at the 3-month visit (visit 5) as there had been insufficient healing time following the completion of the initial treatment to justify recording the periodontal indices. A proposal for future research is to drop collection of research data at the 3-month time point (although a visit at around 3 months may still be needed for supportive periodontal therapy as part of usual care).

### Relationship to previous research

Previous research [34, 35] in this field has rarely provided in-depth descriptions of participant recruitment, although 6-month attrition rates are comparable to those reported in this study.

The eligibility criteria (medical conditions) used in the current study, although more specifically defined than previous research [36–38], did not appear to adversely affect eligibility rates (based on the limited data we have from the NDH periodontal new patient clinic in which only one out of 391 patients was ineligible on medical grounds), and similar criteria could be used in future studies.

The minimum number of teeth required to be eligible for this study was reduced from 20 to 16 part way through the study (after 7 months’ recruitment). We found that, due to the severe nature of periodontitis in some smokers, many of the potential participants had suffered from tooth loss previously and had less than 20 teeth remaining. We reviewed the reason for using 20 teeth as a cut-off in previous studies and found this to be arbitrary. We decided to revise our lower limit to 16 teeth which represents half of the dentition of a normal adult, giving a fair representation of the disease profile and enough teeth for the periodontal measures to be useful and for samples to be collected.

The control group in the current study received SCA as part of usual care, achieving a 5% quit rate (RS6). This is similar to the rates discussed in a Cochrane systematic review of brief advice interventions delivered by physicians, which concluded that the brief advice intervention could increase 6-month quit rates to 5% from the 3% unassisted rate [39]. Another Cochrane systematic review focusing on smoking cessation within the dental setting concluded that quit rates could be increased to 7% [40]. It was interesting to note that both of the RS6 quitters in the control group in our study used a self-purchased e-cigarette (against instructions) as part of their quit attempt. The intervention group in the current study achieved a 15% quit rate (RS6) which is similar to previous research (7.3–12%) [37, 38] and comparable to the longer term quit rates seen in specialist stop smoking services [41, 42].

In our study, there was contamination of the control group, with 20% of participants in the control group using an e-cigarette at some point. Previous studies [37, 38] have not reported on this, which has likely become more of an issue with the widespread availability and popularity of e-cigarettes in recent years. Future research should utilise a highly pragmatic design, accepting a level of e-cigarette use by participants in the control group.

There were 56 AEs mainly associated with the sequelae of severe periodontitis, e.g. toothache, dentine hypersensitivity, tooth loss and abscesses. Direct comparison with previous research is difficult as AEs have rarely been reported. Five of the AEs reported in the intervention group could have been associated with e-cigarette use or smoking cessation (mouth ulceration and intra-oral soft tissue soreness). The Cochrane review on e-cigarettes [9] found that the most frequently reported adverse events were mouth and throat irritation.

### Strengths and limitations

#### Strengths

To the best of our knowledge, this is the first RCT of e-cigarettes in the dental setting or focusing on oral health. This study was feasibility in scale, with 58 participants completing it. However, this compares favourably to the previously conducted prospective studies in this field, which reported completion numbers of 26 (12-month follow-up) [34] and 63 (6-month follow-up) [35]. The feasibility study and pilot RCT complied with reporting guidelines; the CONSORT checklist for pilot and feasibility studies [13] has been used, and the study complies with all the relevant checklist items. Similarly, the interventions provided in this study were reported in detail using the Template for Intervention Description and Replication (TiDieR) checklist, enhancing transparency and reproducibility [43]. Our findings will allow for a well-designed and efficient definitive study in order to answer a research question evaluating the effectiveness, cost-effectiveness and safety of an e-cigarette intervention for smoking cessation delivered alongside periodontal therapy and if this leads to improved periodontal outcomes.

#### Limitations

The contamination rate of the control group (using e-cigarettes, against instructions) was an important finding of the current study. On the one hand, this adds to the reasons why these pilot RCT results should be interpreted cautiously, but on the other, it is an important feasibility outcome that will shape the design of a future definitive study.

The current study was conducted in a single specialist DCRF within a secondary care environment (NDH), in which the chief investigator and principal investigator were based. This allowed for dedicated experienced research teams to conduct high-quality research, but applicability to primary care may be reduced compared to a study conducted in a primary care environment. Future studies should consider this in their design. Additionally, the SCA was delivered by a single clinician in this pilot RCT, unlike a future trial which would have multiple operators.

The control group in the current study appeared to have more severe periodontitis at baseline than the intervention group. Ideally, the groups should have balanced disease profiles, and hence, a future definitive study should stratify for periodontitis severity.

Blinding was not possible for the collection of the smoking outcome measures, mainly for practical reasons, e.g. staffing limitations. West et al. [18] discuss the concern that lack of blinding may lead to differential efforts being devoted to contacting subjects in different treatment groups, and they recommend that follow-up rates should be reported by group. We reported detailed follow-up rates by group and found that equal numbers of participants (29 in each group) completed the study. Those participants lost to follow-up appeared to have higher eCO and FTND readings and more severe periodontitis. It is noteworthy that similar studies have also not employed blinding [37, 44].

A common challenge with all e-cigarette research is the rapidly moving pace of the field, particularly with regard to product development and use. The product we used in our study, although still available for sale at the end of the trial, had largely been superseded. The rapid changes in popularity and usage of e-cigarettes also potentially make the applicability of the findings challenging. In order for the findings of the current study to remain of optimal relevance, it is important that a definitive study is instigated rapidly.

### Implications for future research

Overall, this feasibility study with embedded pilot RCT demonstrated that the offer of an e-cigarette starter kit by a dentist to smokers with periodontitis was feasible and acceptable. Its evaluation within this context was possible, and there are several design implications for a future definitive study. These include expected recruitment and retention rates; conducting part of the research in primary care; eligibility criteria in relation to numbers of teeth; not including willingness to quit as an inclusion criterion; study design to be highly pragmatic (broad inclusion criteria, conducted in primary dental care and the e-cigarette intervention having a range of flavour choices, including tobacco and non-tobacco flavours); stratification based on periodontitis severity; not collecting research data at the 3-month time point; reducing the number of outcome measures collected; not using the weekly smoking questionnaire (or collecting such data less frequently); having co-primary outcomes (smoking abstinence rates and a measure of oral/periodontal health); and a sample size based upon the pilot RCT data.

## Conclusions

It was feasible and acceptable to provide an e-cigarette intervention for smoking cessation, for patients with periodontitis, within the dental setting. The results suggest that the e-cigarette intervention may improve smoking quit rates. The findings of this study will inform the design and sample size of a future definitive study.

## Additional files

Additional file 1: CONSORT checklist. Completed CONSORT checklist. (DOCX 21 kb)

Additional file 2: Protocol amendments. Chronological listing of all protocol amendments with reasons. (DOCX 12 kb)

Additional file 3: Smoking cessation advice TiDieR checklist. A TiDieR checklist for the smoking cessation advice intervention. References: [8, 39]. (DOCX 17 kb)

Additional file 4: E-cigarette starter kit contents. Detailed description of the e-cigarette starter kit contents. (DOCX 12 kb)

Additional file 5: E-cigarette intervention TiDieR checklist. A TiDieR checklist for the e-cigarette intervention. (DOCX 13 kb)

Additional file 6: Oral hygiene instruction TiDieR checklist. A TiDieR checklist for the oral hygiene instruction intervention. (DOCX 14 kb)

Additional file 7: Outcome measures collection details. Descriptions of the collection methods for the outcome measures. References: [16–21, 23, 45, 46]. (DOCX 29 kb)

Additional file 8: Schedule of events. (DOCX 15 kb)

Additional file 9: Statistical analysis plan. (PDF 980 kb)

Additional file 10: Recruitment source of study participants. Detailed breakdowns of the participant recruitment sources. (DOCX 12 kb)

Additional file 11: Eligibility outcomes. Eligibility outcomes from one of the recruitment sources (periodontal new patient clinic). (DOCX 12 kb)

Additional file 12: Participant follow-up by randomisation group. Detailed follow-up data by randomisation group, including compliance with target visit window. (DOCX 13 kb)

Additional file 13: Baseline characteristics of those lost to follow-up. Baseline characteristics of those lost to follow up by randomisation group. (DOCX 21 kb)

Additional file 14: Compliance with attending follow-up visits. Summary participant compliance with attending follow-up visits, including by recruitment source. (DOCX 13 kb)

Additional file 15: Compliance with attending follow-up visits. Summary participant compliance with attending follow-up visits, including by recruitment source. (DOCX 27 kb)

Additional file 16: E-liquid flavour and strength selections. Detailed breakdown of the e-liquid flavour and strength selections. (DOCX 12 kb)

Additional file 17: Use of non-recommended e-liquid or device. Details of those participants who used non-recommended e-liquid or devices during the study. (DOCX 17 kb)

Additional file 18: Compliance with e-cigarette usage (intervention group). Compliance of the intervention group with e-cigarette usage. (DOCX 12 kb)

Additional file 19: Qualitative process evaluation. Further details of the qualitative process evaluation with illustrative quotes. (DOCX 28 kb)

Additional file 20: E-cigarette use in the control group. Details of those participants in the control group who used e-cigarettes. (DOCX 12 kb)

Additional file 21: Methods of smoking cessation used by randomisation group. Description of the methods of smoking cessation methods used by the participants. (DOCX 12 kb)

Additional file 22: Data completeness. Details of data completeness for the outcome measures. (DOCX 15 kb)

Additional file 23: Sample size calculation and pooled standard deviations of outcome measures. Sample size calculation and pooled standard deviations of periodontal outcome measures. References: [33, 36–38, 40, 47–56]. (DOCX 30 kb)

## Abbreviations

AE: Adverse event
BOP: Bleeding on probing
CAL: Clinical attachment level
CI: Confidence interval
CODS: Clinical oral dryness score
CONSORT: Consolidating Standards of Reporting Trials
DCRF: Dental clinical research facility
eCO: Expired air carbon monoxide
FTA: Failed to attend
FTND: Fagerstrom Test of Nicotine Dependence
GCF: Gingival crevicular fluid
ISRCTN: International Standard Randomised Controlled Trial Number
ITT: Intention to treat
LQ: Lower quartile
MGI: Modified Gingival Index
MPSS: Mood and Physical Symptoms Scale
NCSCT: National Centre for Smoking Cessation and Training
NDH: Newcastle Dental Hospital
NHS: National Health Service
NICE: National Institute of Clinical Excellence
NIHR: National Institute for Health Research
NRT: Nicotine replacement therapy
OHI: Oral hygiene instruction
OHQoL-UK: UK Oral Health-related Quality of Life measure
PC: Periodontal new patient clinic
PESA: Periodontal epithelial surface area
PI: Plaque index
PIC: Participant identifying centre
PIS: Participant information sheet
PISA: Periodontal inflamed surface area
PPD: Pocket probing depth
RCT: Randomised controlled trial
RS6: Russell Standard 6-month quitter
SA: Salivary anabasine
SAE: Serious adverse event
SC: Salivary cotinine
SCA: Smoking cessation advice
SD: Standard deviation
TiDieR: Template for Intervention Description and Replication
UQ: Upper quartile
