# Supplementary material for: A feasibility study with embedded pilot randomised controlled trial and process evaluation of electronic cigarettes for smoking cessation in patients with periodontitis
Source: Pilot Feasibility Stud. 2019 Jun 4;5:74. doi: 10.1186/s40814-019-0451-4 (PMC6547559; doi:10.1186/s40814-019-0451-4)
Supplement: Supplementary file 2 — Protocol amendments. Chronological listing of all protocol amendments with reasons. (DOCX 12 kb) [file 40814_2019_451_MOESM2_ESM.docx]

**Additional file 2. Protocol amendments with reasons**

| **Amendment** | **Details** | **Approved Date** |
| --- | --- | --- |
| Protocol changes | Non-substantial changes were made to the protocol. Change in the way periodontitis was classified and several minor administrative changes. The classification changes comprised the removal of the BOP criterion as it was proving to be an unreliable index to use in smokers (smoke affects the periodontal vasculature making this hard to interpret). Additionally the term ‘sites’ was used instead of ‘teeth’ in order to aid recruitment to this feasibility study. | 04/11/16 |
| Addition of PICs | Submitted as a ‘substantial amendment’ but downgraded by the HRA to a non-substantial amendment (Category B). | 09/11/2016 |
| Protocol changes and use of promotional materials | Non-substantial changes were made to the protocol. The minimum number of teeth required was reduced from 20 to 16 (16 teeth represents 50% of the dentition of a normal adult).  Promotional materials were developed to enhance recruitment at existing recruitment centres. | 09/05/2017 |
| Extension of study end date | Non-substantial amendment to extend the recruitment period and study end date by 4 months.  Original recruitment end date: 20/09/2017.  Amended recruitment end date: 20/01/2018  Original study end date: 31/03/2018  Amended study end date: 31/07/2018 | 27/07/2017 |
