# Supplementary material for: A feasibility study with embedded pilot randomised controlled trial and process evaluation of electronic cigarettes for smoking cessation in patients with periodontitis
Source: Pilot Feasibility Stud. 2019 Jun 4;5:74. doi: 10.1186/s40814-019-0451-4 (PMC6547559; doi:10.1186/s40814-019-0451-4)
Supplement: Supplementary file 3 — Smoking cessation advice TiDieR checklist. A TiDieR checklist for the smoking cessation advice intervention. References: [8, 39]. (DOCX 17 kb) [file 40814_2019_451_MOESM3_ESM.docx]

**Additional file 3. TiDieR checklist: Smoking cessation advice**

| **No.** | **Item** | **Definition** |
| --- | --- | --- |
| 1 | Brief Name | Smoking Cessation Advice (SCA) |
| 2 | Why | A Very Brief Advice intervention, within a medical setting, has been shown to have a significant increase in the rate of quitting (RR 1.66) [1]. Oral health care professionals are in an opportunistic position to deliver a smoking cessation intervention. They can provide advice to quit on medical grounds with very powerful patient specific prompts (e.g. radiographs). Specifically for patients with periodontitis, stopping smoking prior to the delivery of their periodontal intervention will lead to significant, visually obvious and relatively rapid improvements. |
| 3 | What (materials) | If the patient had a panoramic radiograph this was used as a prompt to demonstrate any periodontal disease diagnosis, bone loss and likely impact of smoking on the mouth. If no radiographs were available then other prompts such as tooth staining and bad breath were used as personal prompts. |
| 4 | What (procedure) | The SCA is a short behavioural based intervention based around three domains:   - ASK: Ask and record the smoking status (current smokers, ex-smoker, non-smoker)? - ADVISE: Advise on the likely impact of smoking on the mouth, specifically periodontitis (using patient specific prompts where appropriate e.g. panoramic radiograph). Advise on the best way to quitting (the best way of stopping smoking is with a combination of medication and specialist support). - ACT: Act on patient’s response. Build confidence, give information and refer. Patients are up to four times more likely to quit successfully with support.   A referral was available to the local stop smoking services. A suggested quit date of visit 2 (initial visit of periodontal therapy) was suggested. |
| 5 | Who provided | A dentist provided the SCA. All those providing SCA had completed the NCSCT e-learning module ‘Very Brief Advice on Smoking’ [2]. |
| 6 | How | The SCA was delivered at an individual level, by the dentist, integrated as part of a dental visit. |
| 7 | Where | Dental Surgery, Dental Clinical Research Facility, Newcastle Dental Hospital. |
| 8 | When and how much | The SCA was specifically delivered during study visit 1 (the dental visit prior to the commencement of the periodontal therapy). During this visit the periodontal diagnosis was discussed with the patient, oral hygiene instruction given and the SCA provided. The duration of the SCA was meant to be between 2-5 minutes depending on the response of the participant. The SCA was reinforced at each subsequent dental visit, dependent on the individual participant. |
| 9 | Tailoring | The duration of the SCA was dependent on the engagement of the individual participant.  Participants received supportive advice, at follow up visits, tailored to their level of engagement. As a minimum a 30 second SCA was delivered at each dental visit. As indicated above, prompts were tailored to patient circumstances. |
| 10 | Modifications | NA |
| 11 | How well (Planned) | The SCA training was delivered by a national organisation. Dentists also received training on discussing the effects of smoking on oral health as part of their undergraduate degrees.  The SCA delivered during the first study visit was audio-recorded and a sample checked for implementation fidelity against the SCA flow diagram. |
| 12 | How well (Actual) | A sample of 10 random audio-recordings were checked by a research dental nurse. The average duration was 3 minutes and 12 seconds (ranging from 1 minutes and 10 seconds to 4 minutes and 53 seconds). All the sample contained the three elements of the SCA intervention (Ask, Advise, Act). |

1. Stead LF, Buitrago D, Preciado N, Sanchez G, Hartmann-Boyce J, Lancaster T. Physician advice for smoking cessation. The Cochrane database of systematic reviews. 2013:Cd000165.

2. National Centre for Smoking Cessation and Training. Very Brief Advice training module. 2012. <http://www.ncsct.co.uk/publication_very-brief-advice.php>. Accessed 13/12/2018.
