# Supplementary material for: A feasibility study with embedded pilot randomised controlled trial and process evaluation of electronic cigarettes for smoking cessation in patients with periodontitis
Source: Pilot Feasibility Stud. 2019 Jun 4;5:74. doi: 10.1186/s40814-019-0451-4 (PMC6547559; doi:10.1186/s40814-019-0451-4)
Supplement: Supplementary file 4 — E-cigarette starter kit contents. Detailed description of the e-cigarette starter kit contents. (DOCX 12 kb) [file 40814_2019_451_MOESM4_ESM.docx]

Additional file 4. E-cigarette starter kit contents

| Item | Quantity | Details |
| --- | --- | --- |
| Vype eTank clearomizer (tank) | 2 |  |
| 650mAh battery | 2 |  |
| USB charging cable | 1 |  |
| UK plug | 1 |  |
| Manufacturer’s users’ guide | 1 |  |
| 10ml e-liquid | 2 | Flavour options: Blended Tobacco, Crisp Mint, Dark Cherry and Vpure (flavourless)*.  Nicotine strength concentrations: 0mg/ml, 6mg/ml, 12mg/ml, 18mg/ml. |

Manufacturer details: The Vype product was produced by Nicoventure Trading Limited (Blackburn, UK) who are a subsidiary of British American Tobacco PLC (BAT, London, UK). The products for this study were procured through our NHS hospital pharmacy either through a NHS wholesaler (AAH Pharmaceuticals, Alliance Healthcare) or directly from the manufacturer (plugs).

*The e-liquid flavours will be referred to as tobacco, mint, cherry and flavourless respectively throughout the rest of this thesis.
