# Supplementary material for: A feasibility study with embedded pilot randomised controlled trial and process evaluation of electronic cigarettes for smoking cessation in patients with periodontitis
Source: Pilot Feasibility Stud. 2019 Jun 4;5:74. doi: 10.1186/s40814-019-0451-4 (PMC6547559; doi:10.1186/s40814-019-0451-4)
Supplement: Supplementary file 5 — E-cigarette intervention TiDieR checklist. A TiDieR checklist for the e-cigarette intervention. (DOCX 13 kb) [file 40814_2019_451_MOESM5_ESM.docx]

Additional file 5. TiDieR checklist: e-cigarette intervention

| **No.** | **Item** | **Definition** |
| --- | --- | --- |
| 1 | Brief Name | E-cigarette intervention |
| 2 | Why | E-cigarettes have seen a significant rise in popularity in recent years and there is a growing body of evidence that they are an attractive and effective smoking cessation/ harm reduction tool. E-cigarettes are easy for dentists to recommend and/or provide. |
| 3 | What (materials) | The participants were provided with a second generation (tank) e-cigarette. A starter kit was provided as detailed in Additional file 4. Participants had a choice of four flavour and nicotine concentrations. An information sheet was provided which provided information on setting up the e-cigarette, and where to purchase further e-liquids and tanks. |
| 4 | What (procedure) | The dentist provided the e-cigarette starter kit and e-liquid to each participant in the intervention arm of the study. They practically demonstrated the e-cigarette set up with each participant. They talked through the information sheets and answered any questions. |
| 5 | Who provided | A dentist provided the e-cigarette and training. |
| 6 | How | The e-cigarette training was delivered as a conversation with the e-cigarette as a prompt. |
| 7 | Where | Dental Surgery, Dental Clinical Research Facility (DCRF), Newcastle Dental Hospital (NDH). |
| 8 | When and how much | The e-cigarette intervention was delivered directly following the SCA intervention and was expected to be 10-15 minutes in duration. |
| 9 | Tailoring | The e-cigarette intervention was the same for all participants, with patient choice in respect of flavour and concentration of e-liquid respected. Participants were able to request further support at subsequent appointments as required. |
| 10 | Modifications | NA |
| 11 | How well (Planned) | The dentist providing the e-cigarette intervention followed the information sheet as a prompt and had a discussion guide. The e-cigarette intervention was audio-recorded and a sample checked for implementation fidelity. |
| 12 | How well (Actual) | A sample of 10 random audio-recordings were checked against the 22 items in the discussion guide by a research dental nurse. The average duration was 9 minutes and 29 seconds. Nineteen items were present 100% of the time. Item 3 (‘You are still free to use any of the other ways to stop smoking on top of this’) was absent on one occasion, although this was likely to have been delivered at another non-recorded time point. Items 15 (introducing the users guide) was absent on three occasions, although this was implied by the subsequent use of the users guide. Item 16 (highlighting the manufacturer’s users’ guide at the back of the box) was absent on three occasions. |
