# Supplementary material for: A feasibility study with embedded pilot randomised controlled trial and process evaluation of electronic cigarettes for smoking cessation in patients with periodontitis
Source: Pilot Feasibility Stud. 2019 Jun 4;5:74. doi: 10.1186/s40814-019-0451-4 (PMC6547559; doi:10.1186/s40814-019-0451-4)
Supplement: Supplementary file 6 — Oral hygiene instruction TiDieR checklist. A TiDieR checklist for the oral hygiene instruction intervention. (DOCX 14 kb) [file 40814_2019_451_MOESM6_ESM.docx]

**Additional file 6. TiDieR checklist: oral hygiene instruction**

| **No.** | **Item** | **Definition** |
| --- | --- | --- |
| 1 | Brief Name | Oral Hygiene Instruction (OHI) |
| 2 | Why | Obtaining a satisfactory level of oral hygiene is an important factor in the success of periodontal interventions. |
| 3 | What (materials) | As appropriate:   - Inter-dental cleaning aids: dental floss, inter-dental brushes - Single tufted brush - Manual toothbrush (demo only) - Powered toothbrush (demo only) - Dental demonstration model |
| 4 | What (procedure) | The dentist/hygienist presented information on caries and/or gingivitis/periodontitis; oral hygiene instruction was given based on plaque scores. The individual’s oral status was reviewed at subsequent visits. |
| 5 | Who provided | A dentist or hygienist. |
| 6 | How | The OHI was delivered as a face-to-face conversation at an individual level. |
| 7 | Where | Dental Surgery, Dental Clinical Research Facility, Newcastle Dental Hospital. |
| 8 | When and how much | The OHI was delivered during one of the initial visits. The OHI duration was 5-10 minutes.  At subsequent visits further OHI was provided as appropriate, often integrated as part of the periodontal therapy. |
| 9 | Tailoring | The OHI followed the same structure for all participants and was tailored according to the participant’s existing level of oral hygiene, level of oral hygiene knowledge and level of engagement. |
| 10 | Modifications | NA |
| 11 | How well (Planned) | The dentist/hygienist who provided the OHI were experienced practitioners. |
| 12 | How well (Actual) | NA |
