# Supplementary material for: A feasibility study with embedded pilot randomised controlled trial and process evaluation of electronic cigarettes for smoking cessation in patients with periodontitis
Source: Pilot Feasibility Stud. 2019 Jun 4;5:74. doi: 10.1186/s40814-019-0451-4 (PMC6547559; doi:10.1186/s40814-019-0451-4)
Supplement: Supplementary file 7 — Outcome measures collection details. Descriptions of the collection methods for the outcome measures. References: [16–21, 23, 45, 46]. (DOCX 29 kb) [file 40814_2019_451_MOESM7_ESM.docx]

**Additional file 7. Outcome measure collection details**

## *Self-reported tobacco and e-cigarettes use*

Participants were asked to verbally report (to the research dentist [Richard Holliday]) their tobacco and e-cigarette use at visits 1, 2, 4, 5 and 6. For tobacco, details on the quantity (number of cigarettes or weight of loose tobacco per day) and type of tobacco (factory-made, hand-rolled) were recorded. For e-cigarettes, the number of days on which e-cigarettes were used, as well as product details, were recorded.

## Cumulative tobacco and e-cigarette use

Participants were asked to complete a weekly questionnaire which asked them to report their average cigarettes/day over the last 7 days. The questionnaire was made available in several mediums to accommodate the participant’s preference: short message service (SMS) text message with an embedded link to a mobile web page, email with embedded link, telephone call or a paper version.

## Expired air carbon monoxide

A calibrated carbon monoxide monitor (piCO SMOKERLYZER, Bedfont Scientific Ltd, Maidstone, UK) was used to measure eCO at visits 1, 2, 4, 5 and 6. A single reading was taken by a non-blinded member of the research team. A reading of 10 parts per million (ppm) or above signified that the participant had smoked tobacco in the preceding 24 hours [1].

## Salivary cotinine and salivary anabasine

Cotinine is a metabolite of nicotine and SC is a biomarker of nicotine exposure which gives high readings in those using tobacco, e-cigarettes or other nicotine replacement products. SA is an alkaloid with a very similar chemical structure to nicotine; SA is found in tobacco but not NRT or e-cigarettes. Anabasine can be used to confirm if a participant using NRT/e-cigarettes had also obtained nicotine from tobacco.

Saliva samples were obtained at least one hour following the last consumption of food, drink or medication by the participant and at least one hour following the last episode of oral hygiene (toothbrushing, flossing, mouthrinses etc). Samples were collected at any time during the working day. Participants were seated in the dental chair, without distraction, noise, or conversation. A Salivette® (Sarstedt, Nümbrecht, Germany) was labelled with a sample identifier number and date. Participants were advised to remove the stopper, whilst holding the suspended insert and to place the swab under the tongue by tipping the Salivette® close to the mouth. Participants were not allowed to touch the swab. The swab was left in place until ‘soggy’ before transferring directly from the mouth to the suspended insert. The stopper was replaced and the Salivette® immediately placed on ice, transferred to the laboratory and placed in a -80^o^C freezer. The saliva was analysed for concentrations of cotinine and anabasine. This analysis was conducted at an external commercial laboratory (ABS Laboratories,Welwyn Garden City, UK). Samples were stored in -80^o^C freezer within the Cell and Molecular Biosciences Laboratory (Newcastle University) until transfer.

## Fagerstrom test for nicotine dependence

This six-item questionnaire is designed to assess the degree of dependence among smokers coming to a smoking cessation clinic [2]. It produces a score between 0-10 with higher scores representing more dependence (see Appendix N). The FTND was completed at visits 1, 2, 4, 5 and 6.

## Mood and physical symptoms scale

This 12-item questionnaire assesses cigarette withdrawal symptoms [3]. The ratings can be analysed individually or totalled together to give composite scores. Scores were combined to give four scores: MPSS(Mood [M]) [items 1-7], MPSS(Cravings [C]) [items 8 and 9], MPSS(Physical [P]) [items 10-12] and MPSS(total) [items 1-12] [4] (see Appendix O).

## Smoking abstinence measures

Smoking abstinence was reported in six different categories. In increasing strictness these comprised: self-reported quitter, eCO-verified self-reported quitter, SC/SA-verified self-reported quitter, eCO- and SC/SA-verified self-reported quitter, Russell Standard 6-month quitter based upon eCO (RS6-eCO) and the Russell Standard 6-month quitter based upon eCO and salivary analysis (RS6-S).

## Pocket probing depths

A single trained and calibrated hygienist, blinded to group allocation, collected the PPDs using a manual University of North Carolina (UNC)-15 periodontal probe to record the probing depths to the nearest millimetre. Probing depth was defined as the distance from the probe tip (assumed to be at the base of the pocket) to the free gingival margin. This was recorded at six sites per tooth at visits 1, 5 and 6.

## Modified gingival index

A gingival index based on the Lobene Modified Gingival Index [5] was used to rate the gingival inflammation on a scale of zero to four. This index was assessed by the blinded research hygienist. Table 3.8 detail the parameters of the scale. This index was recorded at six sites per tooth at visit 1, 5 and 6.

## Plaque index

The plaque index of Silness and Loe [6] was employed to measure plaque (without disclosing), rating it on a scale of zero to three. This index was assessed by the blinded research hygienist. This was recorded at six sites per tooth at visit 1, 5 and 6. Table 3.9 details the parameters of the scale.

## Clinical attachment loss

Gingival recession was defined as the distance from the free gingival margin to the cemento-enamel junction. It was recorded to the nearest mm using a manual UNC-15 periodontal probe. Gingival recession was indicated as a positive number and gingival overgrowth was recorded as zero. CAL was calculated by adding the gingival recession and PPD measurements. This measurement was collected by the blinded research hygienist, at visit 1, 5 and 6.

## Bleeding on probing

Following probing, each site was assessed for bleeding on probing. If bleeding occurred within 30 seconds of probing, a score of one was assigned for the site, otherwise, a score of zero was assigned. This measurement was collected by the blinded research hygienist, recorded at six sites per tooth at visit 1, 5 and 6.

## Clinical oral dryness score

Oral dryness (xerostomia) was measured using a ten-item scale as described by Osailan *et al.* [7], giving scores from zero to ten. This index was collected by the blinded research hygienist, at visit 1, 5 and 6. Table 3.10 details the ten items on the scale (each item is assigned a score of one if present).

## Oral health quality of life assessment

The OHQoL-UK questionnaire [8] was used to measure oral health-related quality of life at visit 1 and 6. The 16 items allow responses in either a positive or negative (bidirectional) manner to a series of statements about the effect of oral health on specific aspects of respondents’ daily lives (see Appendix P). The responses range from “very bad” (score 1) to “very good” (score 5). Responses are then summed to give a total score. The lower the score the poorer the OHQoL. McGrath and Bedi (9) reported this questionnaire to have good validity and reliability for assessing the impact of oral health on life quality.

## Modified gingival index [5]

| **Score** | **Description** |
| --- | --- |
| 0 | Absence of inflammation |
| 1 | Mild inflammation; slight change in colour, little change in texture of any portion of but not the entire margin or papillary gingival unit |
| 2 | Mild inflammation; but involving entire margin or papillary unit |
| 3 | Moderate inflammation; glazing, redness, oedema and/or hypertrophy of margin or papillary unit |
| 4 | Severe inflammation; marked redness, oedema and/or hypertrophy of marginal or papillary gingival unit, spontaneous bleeding, congestion, or ulceration |

Plaque index [6]

| **Score** | **Description** |
| --- | --- |
| 0 | No plaque |
| 1 | A thin film of plaque at the gingival margin which may be seen only after running the probe along the tooth surface |
| 2 | Moderate accumulation of plaque deposits which can be seen with the naked eye |
| 3 | Extensive accumulation of plaque deposits |

Clinical oral dryness score [7]

| **Item** | **Description** |
| --- | --- |
| 1 | Mirror sticks to buccal mucosa |
| 2 | Mirror sticks to tongue |
| 3 | Frothy saliva |
| 4 | No saliva pooling in floor of mouth |
| 5 | Tongue shows loss of papillae |
| 6 | Altered/smooth gingival architecture |
| 7 | Glassy appearance of other oral mucosa, especially palate |
| 8 | Tongue lobulated/fissured |
| 9 | Active or recently restored (last 6 months) cervical caries (>2 teeth) |
| 10 | Debris on palate (excluding under dentures) |

1. West R, Hajek P, Stead L, Stapleton J. Outcome criteria in smoking cessation trials: proposal for a common standard. Addiction. 2005;100:299-303.

2. Heatherton TF, Kozlowski LT, Frecker RC, Fagerstrom KO. The Fagerstrom Test for Nicotine Dependence: a revision of the Fagerstrom Tolerance Questionnaire. Br J Addict. 1991;86:1119-27.

3. West R, Hajek P. Evaluation of the mood and physical symptoms scale (MPSS) to assess cigarette withdrawal. Psychopharmacology (Berl). 2004;177:195-9.

4. National Centre for Smoking Cessation and Training. Mood and Physical Symptoms Scale (MPSS). 2012 <http://www.ncsct.co.uk/usr/pub/Mood%20and%20physical%20symptoms%20scale%20(MPSS).pdf>. Accessed 11/08/2018.

5. Lobene RR, Weatherford T, Ross NM, Lamm RA, Menaker L. A modified gingival index for use in clinical trials. Clinical Preventative Dentistry. 1986;8:3-6.

6. Silness J, Loe H. Periodontal disease in pregnancy. II. Correlation between oral hygiene and periodontal condition. Acta Odontol Scand. 1964;22:121-35.

7. Osailan SM, Pramanik R, Shirlaw P, Proctor GB, Challacombe SJ. Clinical assessment of oral dryness: development of a scoring system related to salivary flow and mucosal wetness. Oral surgery, oral medicine, oral pathology and oral radiology. 2012;114:597-603.

8. McGrath C, Bedi R. Understanding the value of oral health to people in Britain--importance to life quality. Community Dent Health. 2002;19:211-4.

9. McGrath C, Bedi R. An evaluation of a new measure of oral health related quality of life--OHQoL-UK(W). Community Dent Health. 2001;18:138-43.
