# Supplementary material for: A feasibility study with embedded pilot randomised controlled trial and process evaluation of electronic cigarettes for smoking cessation in patients with periodontitis
Source: Pilot Feasibility Stud. 2019 Jun 4;5:74. doi: 10.1186/s40814-019-0451-4 (PMC6547559; doi:10.1186/s40814-019-0451-4)
Supplement: Supplementary file 8 — Schedule of events. (DOCX 15 kb) [file 40814_2019_451_MOESM8_ESM.docx]

**Additional file 8. Schedule of events**

| Event | Visit 1 (baseline) | | Visit 2  (quit date) | | Visit 3 | | Visit 4  (4 weeks) | Visit 5  (3 months) | Visit 6  (6 months) | | |  |
| --- | --- | --- | --- | --- | --- | --- | --- | --- | --- | --- | --- | --- |
| Randomisation | X |  | |  | |  | |  | |  | |  |
|  |  |  | |  | |  | |  | |  | |  |
| **Periodontal outcome measures** | | | |  | |  | |  | |  | |  |
| No. teeth | X |  | |  | |  | | X | | X | |  |
| PPD | X |  | |  | |  | | X | | X | |  |
| MGI | X |  | |  | |  | | X | | X | |  |
| PI | X |  | |  | |  | | X | | X | |  |
| CAL | X |  | |  | |  | | X | | X | |  |
| BOP | X |  | |  | |  | | X | | X | |  |
| Microbiological | X |  | |  | |  | | X | | X | |  |
| Inflammatory biomakers | X |  | |  | |  | | X | | X | |  |
| OHQoL-UK | X |  | |  | |  | |  | | X | |  |
| CODS | X |  | |  | |  | | X | | X | |  |
|  |  |  | |  | |  | |  | | |  | |
| **Smoking outcome measures** | | | |  | |  | |  | |  | |  |
| FTND | X | X | |  | | X | |  | | X | |  |
| MPSS | X | X | |  | | X | |  | | X | |  |
| eCO | X | X | |  | | X | |  | | X | |  |
| SC | X | X | |  | | X | |  | | X | |  |
| SA | X | X | |  | | X | |  | | X | |  |
| Self-reported smoking status | X | X | |  | | X | | X | | X | |  |

Visit 2 was designated as the target quit date and was arranged after discussion with the participant with the recommendation that it was ideally within 4 weeks of visit 1. Visit 2 was designated as week 0. Periodontal therapy was delivered during visit 2 and 3. Visit 3 was recommended to be 1-7 days after visit 2. Participants were consented and randomised at visit 1, with randomisation taking place prior to collection of baseline data.
