# Supplementary material for: A feasibility study with embedded pilot randomised controlled trial and process evaluation of electronic cigarettes for smoking cessation in patients with periodontitis
Source: Pilot Feasibility Stud. 2019 Jun 4;5:74. doi: 10.1186/s40814-019-0451-4 (PMC6547559; doi:10.1186/s40814-019-0451-4)
Supplement: Supplementary file 9 — Statistical analysis plan. (PDF 980 kb) [file 40814_2019_451_MOESM9_ESM.pdf]

A mixed methods feasibility study of electronic cigarette use by patients with periodontitis.

STATISTICAL ANALYSIS PLAN

Author: Richard Holliday
Last modified: 5th June 2018

1. Study details

Chief Investigator:	Dr Richard Holliday (RH)
Principal Investigator:	Professor Philip Preshaw
Co – Investigator:	Professor Elaine McColl
Co – Investigator (supervising statistician):	Ms Vicky Ryan (VR)
Co – Investigator:	Professor Falko Sniehotta
Co – Investigator:	Professor Linda Bauld
Co – Investigator:	Dr Suzanne McDonald
Co – Investigator:	Dr Nicholas Jakubovics
Senior Trial Manager:	Jared Thornton
ISRCTN Number:	ISRCTN 17731903
REC Reference:	16/NE/0219
Sponsor:	The Newcastle upon Tyne Hospitals NHS Foundation Trust
Sponsor Protocol Number:	1.3
Funder:	National Institute for Health Research (DRF-2015-08-077)

Study objectives: The primary objectives of this feasibility study are to establish if the e-cigarette intervention and associated trial procedures are deliverable and acceptable from the perspectives of a range of stake-holders, in particular patients and health professionals. To this end, a key focus of the study will be on rates of patient eligibility, recruitment, randomisation and retention and the yield and quality of data.

Study design: Randomised, assessor-blind, controlled single centre feasibility study

Study intervention: Usual Care (Very Brief Advice on Smoking and Oral Health) or Usual Care + Electronic Cigarette Provision and Training. (1:1 ratio)

Study site:	Newcastle Dental Hospital
Sample size required:	80
Study duration (recruitment):	15 months

2. Protocol approval and amendment dates

Table 2.1: Protocol approvals and amendment dates

<table>
<tr><th>Protocol version</th><th>Amendment</th><th>Details</th><th>Approved Date</th></tr>
<tr><td>1.0, dated 24/08/2016</td><td>Original submission</td><td></td><td>REC – 03/08/2016</td></tr>
<tr><td>1.1, dated 24/08/2016</td><td>Protocol changes</td><td>Non-substantial changes were made to the protocol following REC approval. This was to confirm who would have access to data and update the sponsor contact details.</td><td>HRA- 02/09/2016

Notified REC- 06/09/2016</td></tr>
<tr><td>1.2, dated 13/10/2016</td><td>Protocol changes</td><td>Non-substantial changes were made to the protocol. Change in the way Periodontitis is classified and several minor administrative changes.</td><td>HRA 4/11/16</td></tr>
<tr><td>1.2, dated 13/10/2016</td><td>Addition of PICs</td><td>Submitted as a ‘substantial amendment’ but downgraded to a non-substantial amendment (Category B).</td><td>HRA 4/11/16

Sponsor sign off- 9/11/2016</td></tr>
<tr><td>1.3, dated 06/04/2017</td><td>Protocol changes and use of promotional materials</td><td>Non-substantial changes were made to the protocol. The minimum number of teeth required was reduced from 20 to 16 (16 teeth represented 50% of the dentition of a normal adult).
Promotional materials were developed to enhance recruitment as existing recruitment centres.</td><td>HRA 09/05/2017</td></tr>
<tr><td>1.3, dated 06/04/2017</td><td>Extension of study end date</td><td>Non-substantial amendment to extend the recruitment period and study end date by 4 months.
<table><tr><td>Original recruitment end date:</td><td>20/09/2017</td></tr><tr><td>Amended recruitment end date:</td><td>20/01/2018</td></tr><tr><td></td><td></td></tr><tr><td>Original study end date:</td><td>31/03/2018</td></tr></table></td><td>Sponsor sign off- 21/07/2017.

HRA approval- 27/07/2017.</td></tr>
</table>

		Amended study end date: 31/07/2018	

3. Signing off sheet

Review of Analysis Plan

The following analysis plan for the feasibility study of e-cigarettes in periodontitis study has been reviewed by the following personnel. Any amendments to the plan should be attached to this document and signed by all relevant personnel.

Trial Statistician: Vicky Ryan

Date: 05.06.18

Chief Investigator: Richard Holliday

Date: 05/06/2018

4. Introduction

The trial protocol describes the method of data collection and the main features of the analysis. This document describes, in more detail, the proposed strategy for the statistical analysis and presentation of data collected for this trial, guiding the final analyses.

Both the ICH Guidance on Statistical Principles for Clinical Trials (ICH E9) and the Revised CONSORT Statement for Reporting Randomized Trials recommend that all analyses should be planned and outlined in a statistical analysis plan prior to the unblinding of the data so as to avoid any post hoc decisions which may affect the interpretation of the statistical analyses. The CONSORT statement also recommends that when writing research papers authors should specify whether analyses were planned or suggested by the data – planned analyses have greater credibility and are in line with Good Clinical Practice.

The final trial data will be collected by the RH. These will be stored in SPSS format.

5. Recruitment

- This study aimed to randomise 80 patients.

- The recruitment was initially expected to be over a 12-month period and projected patient accrual was 7 patients/month. The recruitment period was then extended by 4 months.

- Recruitment opened on 20th September 2016 and closed to recruitment on 7th December 2017. Eighty patients were randomised. The last patient to enter the study was randomised on 7th December 2017.

Figure 6.1: Recruitment flow chart

Number of new (periodontal) patients seen on periodontal new patient (PP) clinic, ***n=***

Potentially eligible patients identified by other routes, ***n=* :**
Other new patient clinics: **n=**
Dental Emergency Clinic (DEC): **n=**
PICs: **n=**
StR**: n=**
Student clinic: **n=**

Excluded at new patient clinic (based on study inclusion/exclusion criteria), ***n=***

Reasons:

Reason	n (%)
<20 natural teeth	
Non-smoker	
Smoker but smokes <10 factory-made cigarettes/day	
Using an e-cigarette (3 or more days use in the last 30)	
Periodontitis does not meet criteria	
Pregnant/ nursing	
Periodontal treatment (other than S/P) in last 6 months	
Significant medical history	
Currently undergoing extensive dental, orthodontic, implant or peri-implant treatment	
Declined participation in research study	

Number of eligible/potentially eligible patients, ***n=***

(n% of patients attending PP clinic)

Total number of eligible/ potentially eligible patients, **n=**

Eligible/potentially eligible, but did not take part in the study, ***n=*** (% of eligible/potentially eligible)

Reason:
- Childcare commitments (PP): n=
- Unable to contact (DEC): n=
- FTA (DEC): n=
- Not interested (DEC): n=
- Can't commit (DEC): n=
- Work commitments (DEC): n=
- Can't commit- family issues (PIC): n=
- Can't commit (PIC): n=
- FTA (PIC): n=
- Unable to contact (PIC): n=
- Work commitments (PIC): n=
- FTA (PP): n=

Ineligible **n=**

Reasons: Started vaping (PIC): n=

Number of patients consented and randomised, ***n*** **=**
(n% of eligible/potentially eligible)

Origin:
PP clinic: **n=**
Other new patient clinics: **n=**
Dental Emergency Clinic (DEC): **n=**
PICs: **n=**
StR**: n=**
Student clinic: **n=**

Figure 6.2: Plot of cumulative number of patients randomised by month: actual and predicted.

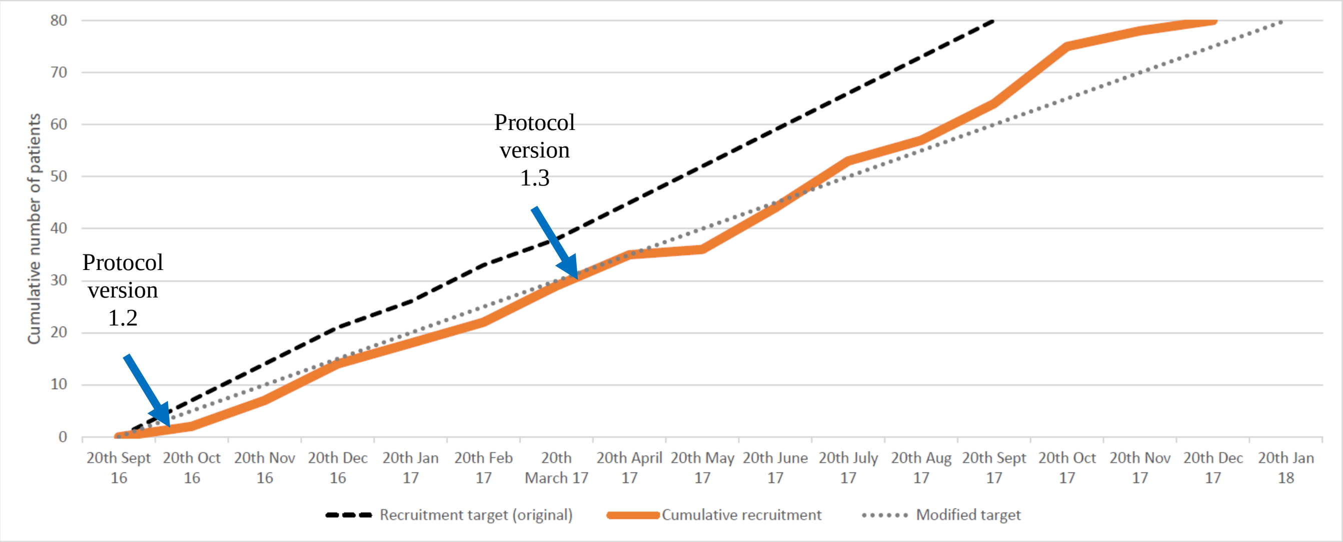

6. Randomisation

Randomisation was through a secure password protected web-based system administered centrally by the Newcastle Clinical Trials Unit (NCTU). Participants were randomised to usual care or the e-cigarette intervention in a 1:1 ratio, using random permuted blocks of size x to y to ensure concealment of allocation.

Xx participants were randomised to the control group and yy participants were randomised to the intervention group.

Treatment arm allocation as recorded in the randomisation log will be checked against actual treatment received as recorded in the CRF and master SPSS (using visit 1 unblinded CRF). Any inconsistencies will be reported, however, analysis will be by intention to treat, i.e. including all randomised participants and retaining participants in their randomised treatment group.

7. Ineligible Participants

Ineligible participants are classed as those randomised participants who are found to subsequently not adhere to the eligibility criteria of the trial (Protocol Section 3.2). The number of known ineligible participants and reasons for ineligibility will be reported. However, the analysis will be by intention to treat, i.e. including all randomised participants and retaining participants in their randomised treatment groups.

Table x: Summary of ineligible participants by randomised treatment group

**Criteria**	**Number (%) of participants**		
	Control group	Intervention group	Total
<18 years of age *at consent*			
<16 natural teeth protocol 1.0/1.1, or <20 natural teeth protocol 1.2*			
TOTAL			

Xx participants had a baseline number of teeth <20 and therefore wouldn't have been eligible for the study with protocol version 1.2.

8. Baseline participant characteristics

Demographic and clinical baseline characteristics at baseline will be compared across treatment groups descriptively. For categorical variables (e.g. gender) the number and percentage in each group will be reported and for continuous variables the mean, standard deviation (sd) or median, IQR/range, as appropriate. Descriptive statistics will be tabulated by treatment group and overall.

No significance testing will be carried out due to the randomised nature of the study.(1-4)

Table 8.1: Baseline assessments, by treatment allocation group (n=80)

**Categorical Baseline Characteristic**<br>n (%)	Control group<br>*n*=xx	Intervention group<br>*n*=yy	Total<br>*n*=80
**Sex**			
Female	xx (xx%)	xx (xx%)	xx (xx%)
Male	xx (xx%)	xx (xx%)	xx (xx%)
**Ethnicity**			
White (British, Irish, other White)	xx (xx%)	xx (xx%)	xx (xx%)
Mixed (White & Black Caribbean, White & Black African, White & Asian, other Mixed)	x (xx%)	x (xx%)	x (xx%)
Asian or Asian British (Indian, Pakistani, Bangladeshi, other Asian)	x (xx%)	x (xx%)	x (xx%)
Black or Black British (Caribbean, African, other Black)	x (xx%)	x (xx%)	x (xx%)
Chinese or other ethnic group	x (xx%)	x (xx%)	x (xx%)
Not Stated	x (xx%)	x (xx%)	x (xx%)
**Occupation**			
Working in a routine or manual occupation	x (xx%)	x (xx%)	x (xx%)
Working in an intermediate occupation	x (xx%)	x (xx%)	x (xx%)
Working in a managerial or professional occupation	x (xx%)	x (xx%)	x (xx%)
Unemployed / not working for a year or more	x (xx%)	x (xx%)	x (xx%)

Full time student		x (xx%)		x (xx%)	x (xx%)
Retired		x (xx%)		x (xx%)	x (xx%)
Sick / Disabled / Unable to return to work		x (xx%)		x (xx%)	x (xx%)
Home carer (unpaid)		x (xx%)		x (xx%)	x (xx%)
None of these		x (xx%)		x (xx%)	x (xx%)
**Continuous Baseline Characteristic**<br>Min<br>Median (LQ-UQ)<br>Mean (SD)<br>Max		Control group<br>*n*=xx		Intervention group<br>*n*=yy	Total<br>*n*=80
Age (years)		xx<br>xx (xx-xx)<br>xx (xx)<br>xx		xx<br>xx (xx-xx)<br>xx (xx)<br>xx	xx<br>xx (xx-xx)<br>xx (xx)<br>xx
Number of cigarettes/day (any)		xx<br>xx (xx-xx)<br>xx (xx)<br>xx		xx<br>xx (xx-xx)<br>xx (xx)<br>xx	xx<br>xx (xx-xx)<br>xx (xx)<br>xx
Number of factory cigarettes/day	n=	xx<br>xx (xx-xx)<br>xx (xx)<br>xx	n=	xx<br>xx (xx-xx)<br>xx (xx)<br>xx	xx<br>xx (xx-xx)<br>xx (xx)<br>xx
Number of hand-rolled cigarettes/day	n=	xx<br>xx (xx-xx)<br>xx (xx)<br>xx	n=	xx<br>xx (xx-xx)<br>xx (xx)<br>xx	xx<br>xx (xx-xx)<br>xx (xx)<br>xx
Age started smoking		xx<br>xx (xx-xx)<br>xx (xx)<br>xx		xx<br>xx (xx-xx)<br>xx (xx)<br>xx	xx<br>xx (xx-xx)<br>xx (xx)<br>xx
Expired air Carbon Monoxide (eCO) reading		xx		xx	xx

[Reading of >10ppm signifies a tobacco smoker]	xx (xx-xx)<br>xx (xx)<br>xx	xx (xx-xx)<br>xx (xx)<br>xx	xx (xx-xx)<br>xx (xx)<br>xx

**Clinical Examination**<br>Min<br>Median (LQ-UQ)<br>Mean (SD)<br>Max	Control group<br>*n*=xx		Intervention group<br>*n*=yy		Total<br>*n*=80
Number of teeth (excluding 3rd molars)	n=	xx<br>xx (xx-xx)<br>xx (xx)<br>xx	n=	xx<br>xx (xx-xx)<br>xx (xx)<br>xx	xx<br>xx (xx-xx)<br>xx (xx)<br>xx
Plaque score<br>[possible range 0-3, 0= no plaque, 3= extensive plaque]	n=	xx<br>xx (xx-xx)<br>xx (xx)<br>xx	n=	xx<br>xx (xx-xx)<br>xx (xx)<br>xx	xx<br>xx (xx-xx)<br>xx (xx)<br>xx
%BOP score<br>[Bleeding On Probing]	n=	xx<br>xx (xx-xx)<br>xx (xx)<br>xx	n=	xx<br>xx (xx-xx)<br>xx (xx)<br>xx	xx<br>xx (xx-xx)<br>xx (xx)<br>xx
Mean MGI<br>[Modified Gingival Index, possible range 0-4, 0= no inflammation, 4= severe inflammation]	n=	xx<br>xx (xx-xx)<br>xx (xx)<br>xx	n=	xx<br>xx (xx-xx)<br>xx (xx)<br>xx	xx<br>xx (xx-xx)<br>xx (xx)<br>xx
Mean Probing Pocket Depth (PPD) [mm]	n=	xx<br>xx (xx-xx)<br>xx (xx)<br>xx	n=	xx<br>xx (xx-xx)<br>xx (xx)<br>xx	xx<br>xx (xx-xx)<br>xx (xx)<br>xx
Mean PPD [mm] of those sites with a baseline probing depth of ≥ 5mm	n=	xx<br>xx (xx-xx)<br>xx (xx)<br>xx	n=	xx<br>xx (xx-xx)<br>xx (xx)<br>xx	xx<br>xx (xx-xx)<br>xx (xx)<br>xx

Mean PPD [mm] of those sites with a baseline probing depth of ≥ 6mm	n=	xx<br>xx (xx-xx)<br>xx (xx)<br>xx	n=	xx<br>xx (xx-xx)<br>xx (xx)<br>xx	xx<br>xx (xx-xx)<br>xx (xx)<br>xx
Mean PPD [mm] of those sites with a baseline probing depth of ≥ 7mm	n=	xx<br>xx (xx-xx)<br>xx (xx)<br>xx	n=	xx<br>xx (xx-xx)<br>xx (xx)<br>xx	xx<br>xx (xx-xx)<br>xx (xx)<br>xx
Mean PPD [mm] of those sites with a baseline probing depth of ≤ 4mm	n=	xx<br>xx (xx-xx)<br>xx (xx)<br>xx	n=	xx<br>xx (xx-xx)<br>xx (xx)<br>xx	xx<br>xx (xx-xx)<br>xx (xx)<br>xx
Mean CAL [mm]<br>[Clinical Attachment Level]	n=	xx<br>xx (xx-xx)<br>xx (xx)<br>xx	n=	xx<br>xx (xx-xx)<br>xx (xx)<br>xx	xx<br>xx (xx-xx)<br>xx (xx)<br>xx
PESA [mm^2]<br>[Periodontal Epithelial Surface Area]	n=	xx<br>xx (xx-xx)<br>xx (xx)<br>xx	n=	xx<br>xx (xx-xx)<br>xx (xx)<br>xx	xx<br>xx (xx-xx)<br>xx (xx)<br>xx
PISA [mm^2]<br>[Periodontal Inflammed Surface Area]	n=	xx<br>xx (xx-xx)<br>xx (xx)<br>xx	n=	xx<br>xx (xx-xx)<br>xx (xx)<br>xx	xx<br>xx (xx-xx)<br>xx (xx)<br>xx
No. of sites with probing depth ≥ 5mm	n=	xx<br>xx (xx-xx)<br>xx (xx)<br>xx	n=	xx<br>xx (xx-xx)<br>xx (xx)<br>xx	xx<br>xx (xx-xx)<br>xx (xx)<br>xx
% of sites with probing depth ≥ 5mm	n=	xx<br>xx (xx-xx)<br>xx (xx)<br>xx	n=	xx<br>xx (xx-xx)<br>xx (xx)<br>xx	xx<br>xx (xx-xx)<br>xx (xx)<br>xx
No. of sites with probing depth ≤ 4mm	n=	xx<br>xx (xx-xx)<br>xx (xx)<br>xx	n=	xx<br>xx (xx-xx)<br>xx (xx)<br>xx	xx<br>xx (xx-xx)<br>xx (xx)<br>xx
% of sites with probing depth ≤ 4mm	n=	xx	n=	xx	xx

		xx (xx-xx)<br>xx (xx)<br>xx		xx (xx-xx)<br>xx (xx)<br>xx	xx (xx-xx)<br>xx (xx)<br>xx
Clinical Oral Dryness Score<br>[Possible range: 0-10, 10= worse dryness]	n=	xx<br>xx (xx-xx)<br>xx (xx)<br>xx	n=	xx<br>xx (xx-xx)<br>xx (xx)<br>xx	xx<br>xx (xx-xx)<br>xx (xx)<br>xx

Degree of precision: An appropriate approach will be taken for each outcome usually to one decimal place.

Table 8.2: Baseline patient completed questionnaires, by treatment allocation group.

**Baseline Questionnaire**<br>Min<br>Median (LQ-UQ)<br>Mean (SD)<br>Max	Control<br>n= xx	Intervention<br>n= yy	Total<br>n= 80
Fagerstrom Test for Nicotine Dependence [(A)]	xx<br>xx (xx-xx)<br>xx (xx)<br>xx	xx<br>xx (xx-xx)<br>xx (xx)<br>xx	xx<br>xx (xx-xx)<br>xx (xx)<br>xx
Mood and Physical Symptoms Scale[(B)]	xx<br>xx (xx-xx)<br>xx (xx)<br>xx	xx<br>xx (xx-xx)<br>xx (xx)<br>xx	xx<br>xx (xx-xx)<br>xx (xx)<br>xx
Oral Health Quality of Life Assessment (OHQoL-UK)[(C)]	xx<br>xx (xx-xx)<br>xx (xx)<br>xx	xx<br>xx (xx-xx)<br>xx (xx)<br>xx	xx<br>xx (xx-xx)<br>xx (xx)<br>xx

A. Fagerstrom Test for Nicotine Dependence. [6 items, possible range: 1-10, higher score= more intense dependence]
B. Mood and Physical Symptoms Scale [12 items, possible range: 10-60, higher score = worse cigarette withdrawal symptoms]
C. Oral Health Quality of Life Assessment [16 items, possible range: 16- 80, lower score = poorer OHRQoL]

9. Follow-up

Participants are scheduled to return for their study assessments at the following time points:

- Visit 4: 4- weeks (minus 3 days or plus 14 days)#
- Visit 5: 3-months (minus 15 days or plus 28 days)*
- Visit 6: 6-months (minus 15 days or plus 28 days) #*

These study window periods are based on the Russel Standards for smoking cessation studies.
*In the CRF the study visit windows for visit 5 and 6 are specified as +/-15 days. Within the SAP a window of minus 15 days or plus 28 days will be used (in line with Russell Standards). Figure 9.1 presents the number of participants in both window sizes.

Participants could withdraw from the study for any reason at any time and no further data or tissue would be collected or any other research procedures carried out on or in relation to the participant.

Participants who failed to attend a scheduled visit and were unreachable were deemed lost to follow-up.

9.1. Withdrawals and loss to follow-up:

There were x withdrawals or loss to follow-up, with y from the intervention group and z from the control group.

Table 9.1: Chronological list of withdrawals and loss to follow-up

Participant No.	Study Arm	Date of randomisation	Withdrawal date/ date of last visit	Study visits completed?	Reason

Table 9.2: Summary of patient follow-up

		Control n=xx	Intervention n=yy	Total n=80
Visit 1 Randomisation		n (%)	n (%)	n (%)
Visit 4 4- weeks (minus 3 days or plus 14 days)	Complied with study window	n (%)	n (%)	n (%)
	Out with study window	n (%)	n (%)	n (%)
Visit 5 3- months (minus 15 days or plus 28 days)	Complied with study window	n (%)	n (%)	n (%)
	Out with study window	n (%)	n (%)	n (%)
Visit 6 6- months (minus 15 days or plus 28 days)	Complied with study window	n (%)	n (%)	n (%)
	Out with study window	n (%)	n (%)	n (%)

Figure 9.1: Compliance with study window at visit 4, 5 and 6.

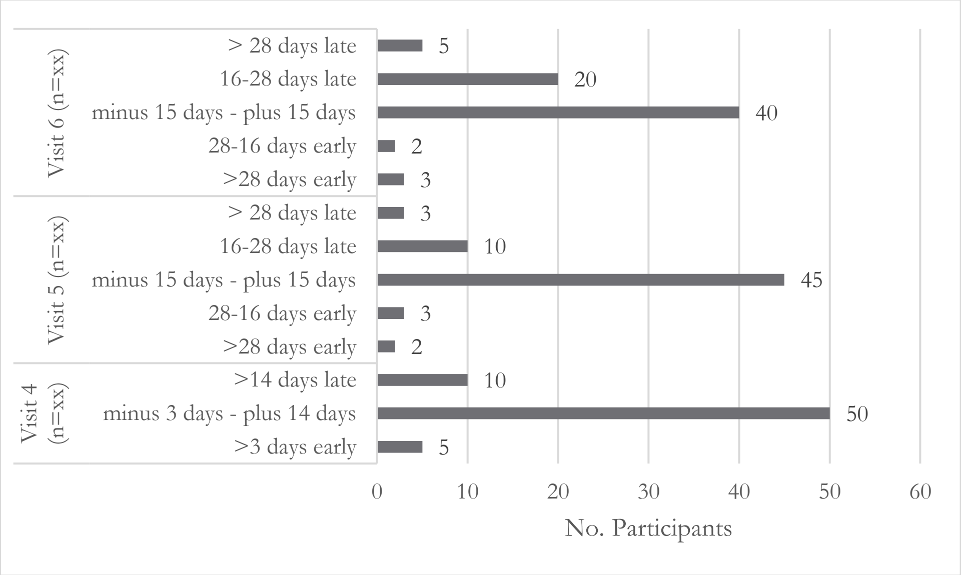

10. Safety data

There have been xx SAEs reported in total.

Table 10.1: Chronological listing of SAEs:

Study ID	Treatment allocation	Date of initial report	SAE Description	Onset Date	Severity	SAE reason	Outcome of SAE

Table 10.2: Summary of Adverse Events (AE)**

There have been xx AE's in zz participants.

AE	No. AE in control group (no. patients)	No. AE in intervention group (no. patients)
Toothache	N (n)	N (n)
Dentine hypersensitivity	N (n)	N (n)
Tooth loss	N (n)	N (n)
Mouth ulceration	N (n)	N (n)
Fractured filling	N (n)	N (n)
Worsening of medical condition	N (n)	N (n)

** A detailed chronological listing of the AEs is available in the appendix

11. Adherence to protocol

Table 11.1: Summary of protocol deviations##

Reason	No. deviations in control group	No. deviations in intervention group
Study visits outside specified window		
Use of e-cigarette whilst in the control group.		NA
Not able to collect periodontal indices at visit 5 due to periodontal therapy being incomplete.		
xxx		

A detailed chronological listing of the protocol deviations is available in the appendix

Participant compliance

This study was intended to be a pragmatic design and hence there was not excessive participant follow up with regards to compliance. Participant compliance will be determined by attendance at the review visits (visit 4,5,6).

Table 11.2: Participant compliance- attendance at review visits

Compliance level	Randomisation group [n (%)]		
	Control group *n*=xx	Intervention group *n*=yy	Total *n*=80
All reviews	n (%)	n (%)	n (%)
2/3 reviews	n (%)	n (%)	n (%)
1/3 reviews	n (%)	n (%)	n (%)
0/3 reviews	n (%)	n (%)	n (%)

Table 11.2: Data completeness

Data element	Visit	Consented for procedure	Completed procedure/ data collected	Data completeness	Comment
Pocket Probing Depths	Baseline	xx	xx	%	
	Visit 5	xx	Xx	%	
	Visit 6	xx	xx	%	
Gingival Index	Baseline	xx	xx	%	
	Visit 5	xx	Xx	%	
	Visit 6	xx	xx	%	
Plaque Index	Baseline	xx	xx	%	
	Visit 5	xx	Xx	%	
	Visit 6	xx	xx	%	
Bleeding on Probing	Baseline	xx	xx	%	
	Visit 5	xx	Xx	%	
	Visit 6	xx	xx	%	
Clinical Attachment Loss	Baseline	xx	xx	%	
	Visit 5	xx	Xx	%	
	Visit 6	xx	xx	%	
Clinical Oral Dryness Score	Baseline	xx	xx	%	
	Visit 5	xx	Xx	%	
	Visit 6	xx	xx	%	
Expired air Carbon Monoxide	Baseline	xx	xx	%	
	Visit 2	xx	Xx	%	
	Visit 4	xx	xx	%	
	Visit 5	xx	Xx	%	
	Visit 6	xx	xx	%	
Saliva sample	Baseline	xx	xx	%	

	Visit 2	xx	Xx	%	
	Visit 4	xx	xx	%	
	Visit 5	xx	Xx	%	
	Visit 6	xx	xx	%	
Subgingival plaque sample	Baseline	xx	xx	%	
	Visit 2	xx	Xx	%	
	Visit 4	xx	xx	%	
	Visit 5	xx	Xx	%	
	Visit 6	xx	xx	%	
Gingival crevicular fluid sample	Baseline	xx	xx	%	
	Visit 5	xx	Xx	%	
	Visit 6	xx	xx	%	
Fagerstrom Test for Nicotine Dependence	Baseline	xx	xx	%	
	Visit 2	xx	Xx	%	
	Visit 4	xx	xx	%	
	Visit 5	xx	Xx	%	
	Visit 6	xx	xx	%	
Mood and Physical Symptoms Scale	Baseline	xx	xx	%	
	Visit 2	xx	Xx	%	
	Visit 4	xx	xx	%	
	Visit 5	xx	Xx	%	
	Visit 6	xx	xx	%	
Oral Health Quality of Life Questionnaire	Baseline	xx	xx	%	
	Visit 6	xx	xx	%	

Compliance with weekly diary

Participant compliance with the weekly data collection will be determined by the proportion of completed entries compared to the number of weeks the participant was in the study.

Table 11.3: Compliance with weekly diary

	Control group<br>*n*=xx	Intervention group<br>*n*=yy	Total<br>*n*=80
Min<br>Median (LQ-UQ)<br>Mean (SD)<br>Max	xx<br>xx (xx-xx)<br>xx (xx)<br>xx	xx<br>xx (xx-xx)<br>xx (xx)<br>xx	xx<br>xx (xx-xx)<br>xx (xx)<br>xx

Table 11.4: Compliance with EC usage (intervention group only)

	Proportion of participants in intervention group (n=yy) (% [n])
Accepted EC starter kit	% (n)
Using the EC at visit 2 (baseline)	% (n)
Using the EC at visit 4 (4w)	% (n)
Using the EC at visit 5 (3m)	% (n)
Using the EC at visit 6 (6m)	% (n)

Table 11.5: E-liquid flavour choice

Each participant chose two e-liquids giving 10 selections. There were four flavour options: tobacco, mint, cherry and flavourless.

	Proportion of participants in intervention group (n=yy) (% [n])
Tobacco only	% (n)
Cherry only	% (n)
Mint only	% (n)
Mint & Cherry	% (n)
Tobacco & Cherry	% (n)
Tobacco & Mint	% (n)
Flavourless only	% (n)
Flavourless & Tobacco	% (n)
Flavourless & Mint	% (n)
Flavourless & Cherry	% (n)

Table 11.6: E-liquid strength (nicotine) choice

Each participant chose two e-liquids giving xx selections. There were four options: 0, 6, 12, 18 mg/ml.

Nicotine choices (mg/ml)		Proportion of participants in intervention group (n=yy) (% [n])
0	0	% (n)
0	6	% (n)
0	12	% (n)
0	18	% (n)
6	6	% (n)
6	12	% (n)
6	18	% (n)
12	12	% (n)
12	18	% (n)
18	18	% (n)

12 Definition of analysis groups

Statistical analyses will be based on the intention to treat principle with analysis groups based on the groups allocated at randomisation and all randomised patients being included in the analysis. The number of participants who did not receive the intervention to which they were randomised will be reported.

Figure 12.1: Consort Flow Diagram

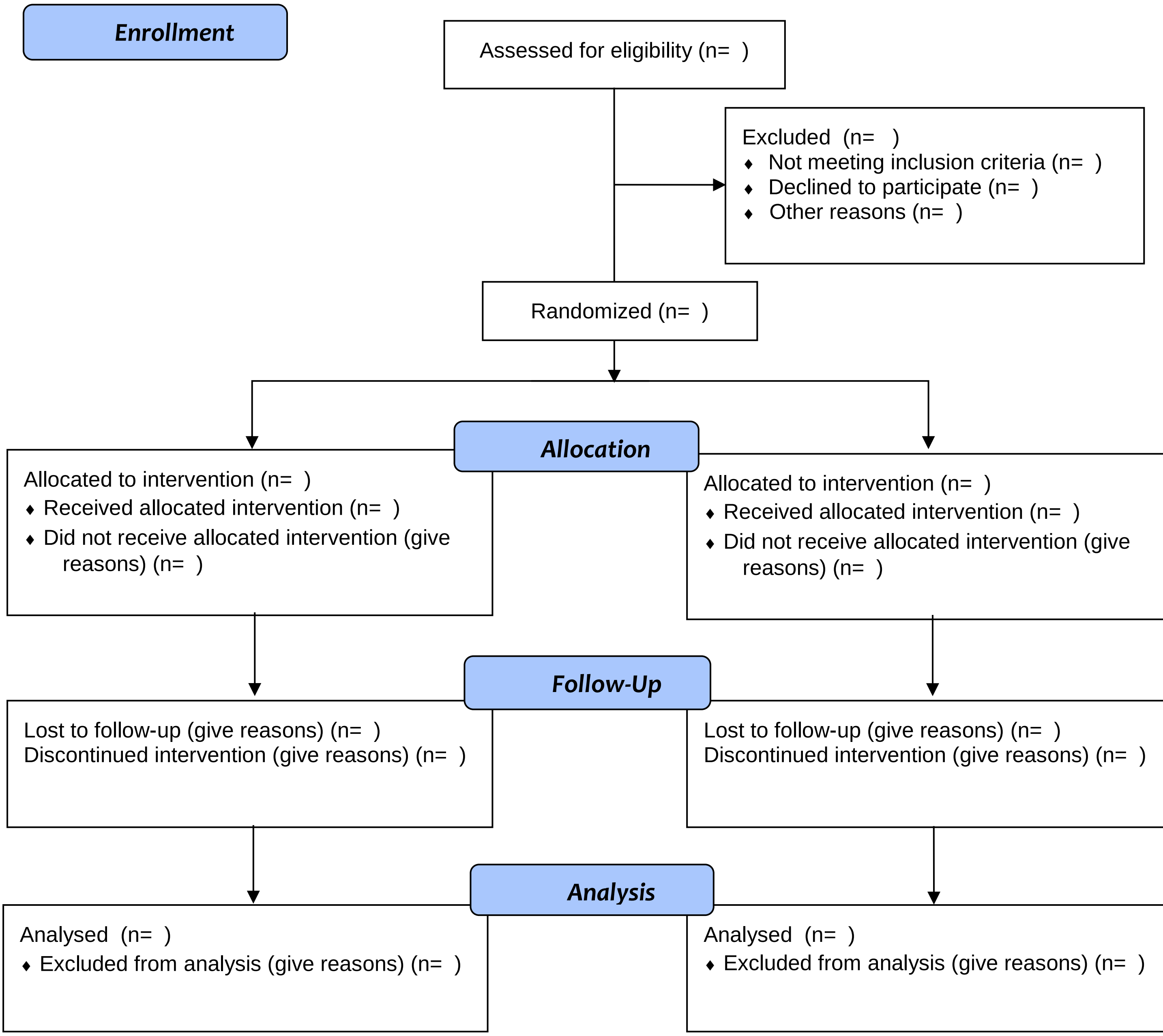

13 Missing data

Smoking outcome data

Those not attending for review visits when smoking outcome data was collected (4w, 3m, 6m) will be considered as continuing smokers or to have relapsed in line with standard research practice (Russell Standard(5) and Cochrane Tobacco Addiction Group(6)). Therefore, the outcomes 'self-reported quitters of burnt tobacco' and 'Russell Standard 6-month quitter (RS6)' will record missing data as non-quitters. For continuous data (eCO, SC, SA) missing data will not be imputed.

Periodontal data

Missing periodontal data due to participant loss to follow-up will not be imputed.

Teeth lost during the study

Teeth that are planned to be removed as part of the initial treatment plan should not be included in any analysis and periodontal indices should not be collected.

Teeth that are lost during the study period (i.e. their loss was not in the initial treatment plan) prior to visit 5 should be recorded as missing data.

Teeth that are lost during the study period (i.e. their loss was not in the initial treatment plan) after visit 5 should follow a 'last observation carried forwards' approach for the periodontal indices. In this circumstance, when a tooth is lost between visit 5 and 6, then the data should be carried forwards from visit 5. This is important because several of the oral health outcome measures for this study are 'summary' whole mouth measures calculated on all teeth present. If a diseased tooth is lost which has high indices, the loss of that tooth could substantially effect the summary whole mouth outcome measures giving the appearance of reduction of disease severity.

Data was carried forwards between visit 5 and 6 for xx participants on yy teeth. The mean (SD) of the carried forwards PPD data was xx(xx).

Table 13.1: Summary of PPD data carried forwards between visit 5 and 6.

	Control group *n*=xx	Intervention group *n*=yy	Total *n*=80
Mean (SD) PPD of data carried forwards between visit 5 and 6	xx (xx)	xx (xx)	xx (xx)

Questionnaires

For missing questionnaire data, published guidelines will be followed for validated questionnaires and otherwise generic rules (such as 'rule of halves') which are recommended in the literature will be described and employed. (7-9)

<u>OHQoLUK</u>
Patients who had not responded to ≥10% of the items in OHQoLUK questionnaire were eliminated and recorded as missing data. For patients who had <10% missing responses, the answers to the missing items were derived using group mean score imputation for each item in order to calculate the individual domain scores and the summary scores as reported in the literature(10-12).

<u>FTND</u>
There are no published guidelines for this questionnaire on how to manage incomplete data. Generic rules will be used.

<u>MPSS</u>
There are no published guidelines for this questionnaire on how to manage incomplete data. Generic rules will be used.

For all questionnaires the completeness of the data will be described by randomised treatment group and overall, at baseline and follow-up visits:

- Missing items per questionnaire (e.g. 95% of OHQoLUKs were complete, 4% had one missing item, etc…)

- Distribution of item responses (% of respondents with missing data by item)

Table 13.2 Distribution (% of respondents) of number of missing questionnaire items

**Questionnaire**	**No. missing items**	**Baseline n (%)**	**Visit 2 n (%)**	**Visit 4 n (%)**	**Visit 5 n (%)**	**Visit 6 n (%)**
OHQoLUK	0	nn (xx%)				nn (xx%)
	1	nn (xx%)				nn (xx%)
	2	nn (xx%)				nn (xx%)
	3	nn (xx%)				nn (xx%)
	4	nn (xx%)				nn (xx%)
	5	nn (xx%)				nn (xx%)
	6	nn (xx%)				nn (xx%)
	7	nn (xx%)				nn (xx%)
	8	nn (xx%)				nn (xx%)
	9	nn (xx%)				nn (xx%)
	10	nn (xx%)				nn (xx%)
	11	nn (xx%)				nn (xx%)
	12	nn (xx%)				nn (xx%)
	13	nn (xx%)				nn (xx%)
	14	nn (xx%)				nn (xx%)
	15	nn (xx%)				nn (xx%)
	16	nn (xx%)				nn (xx%)
FTND	0	nn (xx%)	nn (xx%)	nn (xx%)	nn (xx%)	nn (xx%)
	1	nn (xx%)	nn (xx%)	nn (xx%)	nn (xx%)	nn (xx%)
	2	nn (xx%)	nn (xx%)	nn (xx%)	nn (xx%)	nn (xx%)
	3	nn (xx%)	nn (xx%)	nn (xx%)	nn (xx%)	nn (xx%)
	4	nn (xx%)	nn (xx%)	nn (xx%)	nn (xx%)	nn (xx%)
	5	nn (xx%)	nn (xx%)	nn (xx%)	nn (xx%)	nn (xx%)
	6	nn (xx%)	nn (xx%)	nn (xx%)	nn (xx%)	nn (xx%)
MPSS	0	nn (xx%)	nn (xx%)	nn (xx%)	nn (xx%)	nn (xx%)
	1	nn (xx%)	nn (xx%)	nn (xx%)	nn (xx%)	nn (xx%)
	2	nn (xx%)	nn (xx%)	nn (xx%)	nn (xx%)	nn (xx%)
	3	nn (xx%)	nn (xx%)	nn (xx%)	nn (xx%)	nn (xx%)
	4	nn (xx%)	nn (xx%)	nn (xx%)	nn (xx%)	nn (xx%)
	5	nn (xx%)	nn (xx%)	nn (xx%)	nn (xx%)	nn (xx%)
	6	nn (xx%)	nn (xx%)	nn (xx%)	nn (xx%)	nn (xx%)
	7	nn (xx%)	nn (xx%)	nn (xx%)	nn (xx%)	nn (xx%)
	8	nn (xx%)	nn (xx%)	nn (xx%)	nn (xx%)	nn (xx%)
	9	nn (xx%)	nn (xx%)	nn (xx%)	nn (xx%)	nn (xx%)
	10	nn (xx%)	nn (xx%)	nn (xx%)	nn (xx%)	nn (xx%)
	11	nn (xx%)	nn (xx%)	nn (xx%)	nn (xx%)	nn (xx%)
	12	nn (xx%)	nn (xx%)	nn (xx%)	nn (xx%)	nn (xx%)

Table 13.3 Distribution (% of respondents) of missing items in questionnaires

Questionnaire	Variable	Missing response				
		Baseline n (%)	Visit 2 n (%)	Visit 4 n (%)	Visit 5 n (%)	Visit 6 n (%)
OHQoLUK	Eating or enjoyment of food	nn (xx%)				nn (xx%)
	Appearance	nn (xx%)				nn (xx%)
	Speech	nn (xx%)				nn (xx%)
	General Health	nn (xx%)				nn (xx%)
	Ability to relax or sleep	nn (xx%)				nn (xx%)
	Social life	nn (xx%)				nn (xx%)
	Romantic relationships	nn (xx%)				nn (xx%)
	Smiling or laughing	nn (xx%)				nn (xx%)
	Confidence	nn (xx%)				nn (xx%)
	Carefree manner	nn (xx%)				nn (xx%)
	Mood	nn (xx%)				nn (xx%)
	Work or ability to do your usual jobs	nn (xx%)				nn (xx%)
	Finances	nn (xx%)				nn (xx%)
	Personality	nn (xx%)				nn (xx%)
	Comfort	nn (xx%)				nn (xx%)
	Breath odour	nn (xx%)				nn (xx%)
FTND	Smoke after waking?	nn (xx%)	nn (xx%)	nn (xx%)	nn (xx%)	nn (xx%)
	Refrain from smoking?	nn (xx%)	nn (xx%)	nn (xx%)	nn (xx%)	nn (xx%)
	Which cig give up?	nn (xx%)	nn (xx%)	nn (xx%)	nn (xx%)	nn (xx%)
	How many smoke/day?	nn (xx%)	nn (xx%)	nn (xx%)	nn (xx%)	nn (xx%)
	Morning smoking?	nn (xx%)	nn (xx%)	nn (xx%)	nn (xx%)	nn (xx%)
	Smoke when sick?	nn (xx%)	nn (xx%)	nn (xx%)	nn (xx%)	nn (xx%)
MPSS	Depressed	nn (xx%)	nn (xx%)	nn (xx%)	nn (xx%)	nn (xx%)
	Anxious	nn (xx%)	nn (xx%)	nn (xx%)	nn (xx%)	nn (xx%)

	Irritable	nn (xx%)	nn (xx%)	nn (xx%)	nn (xx%)	nn (xx%)
	Restless	nn (xx%)	nn (xx%)	nn (xx%)	nn (xx%)	nn (xx%)
	Hungry	nn (xx%)	nn (xx%)	nn (xx%)	nn (xx%)	nn (xx%)
	Poor concentration	nn (xx%)	nn (xx%)	nn (xx%)	nn (xx%)	nn (xx%)
	Poor sleep	nn (xx%)	nn (xx%)	nn (xx%)	nn (xx%)	nn (xx%)
	Urge to smoke?	nn (xx%)	nn (xx%)	nn (xx%)	nn (xx%)	nn (xx%)
	Strength of urges?	nn (xx%)	nn (xx%)	nn (xx%)	nn (xx%)	nn (xx%)
	Sore in mouth	nn (xx%)	nn (xx%)	nn (xx%)	nn (xx%)	nn (xx%)
	Constipation	nn (xx%)	nn (xx%)	nn (xx%)	nn (xx%)	nn (xx%)
	Cough/ sore throat	nn (xx%)	nn (xx%)	nn (xx%)	nn (xx%)	nn (xx%)

14 Analysis of outcome measures

In accordance with recommendations for the analysis of feasibility studies (13, 14) (where a formal power calculation is not carried out) the data analyses will be descriptive and statistical comparisons between the randomised groups will not be undertaken.

For the feasibility outcome measures all proportions/rates will be calculated as defined and reported with 95% confidence intervals.

Summaries of the change from baseline to 3m and baseline to 6m for the study assessment outcome measures will be reported as mean (sd) or median (range), depending on the distribution of the data, which will be assessed by examining the data graphically.

Table 14.1: Feasibility outcomes

Trial objective	Assessment method	Outcome
To estimate the eligibility rates among our patient population.	Based on recruitment flow chart	Eligibility rate from Professor Preshaw's new patient clinic.<br><br>(Unable to make any estimates beyond this.)
To assess patients' willingness to enter the trial	Based on eligibility data.	Participants consented as a proportion of those eligible.
To estimate the recruitment rate; can 80 eligible patients be recruited in a 12-month period?	Bases on recruitment data	Unable to recruit within 12 months but successfully completed within 15 months. Average recruitment rate was 5.3/month.
To ascertain if any participation biases exist.	*Not possible to determine this with the data collected.*	-
To ascertain the retention rate of the participants for 6-month follow-up data?	Based on compliance data- 'Table 11.2: participant compliance'.	See Table 11.2
To ascertain the randomised group contamination rates (i.e. the extent of cross-over between the two arms of the trial).	Based on protocol deviations data. See 'Table 11.1: Summary of protocol deviations'	xx% of those in control group who used an e-cigarette.
To test a weekly smoking status data collection method.	Based on compliance with weekly diary data. See 'Table 11.3: Compliance with weekly diary'	See Table 11.3
To compare descriptively novel and traditional periodontal outcome measures. (Novel: PISA, PESA. Traditional: PPDs.)	Based on 'Table 13.2: Oral health outcome measures'	See Table 13.2
To estimate the standard deviation of the periodontal outcome	Based on 'Table 13.2: Oral health outcome measures'	See Table 13.2

measures to input to the sample size calculation for future definitive trials.		
To complete exploratory analyses of the distribution of the microbiome.	Exploratory presence and proportions of bacterial species present will be quantified.	NA
To complete exploratory analyses of the distributional properties of the inflammatory biomarkers.	Based on 'Table 13.2: Oral health outcome measures'	See Table 13.2
To ascertain participant compliance when provided with an EC.	Based on 'Table 11.4: Compliance with EC usage (intervention group only)'	See Table 11.4
To describe tobacco smoking and EC usage.	Based on 'Table 13.3: Smoking related outcome measures' and 'Table 11.4: Compliance with EC usage (intervention group only)'	See Table 11.4 and 13.3.
To ascertain participant behaviour regarding the use of the EC: straight nicotine replacement or nicotine cessation device?	Qualitative TDF interviews	NA
To complete a *Qualitative Process Evaluation* to establish the views of participants on the provision of e-cigarettes and to finalise the exact characteristics of an EC intervention for the future definitive study for this patient group.	Qualitative TDF interviews, staff diary and focus group.	NA

Table 14.2: Oral health outcome measures

Outcome<br><br>Min<br>Median (LQ-UQ)<br>Mean (SD)<br>Max<br>95% CI<br><br>*Unless otherwise detailed*	n	Control Group, n=							n	Intervention Group, n=						
		Baseline (all)	Baseline of those reaching 3-month visit	Baseline of those reaching 6-month visit	Quit date	4-weeks	3-month	6-month		Baseline	Baseline of those reaching 3-month visit	Baseline of those reaching 6-month visit	Quit date	4-weeks	3-month	6-month
Number of teeth (excluding 3rd molars)		xx<br>xx (xx-xx)<br>xx (xx)<br>xx<br>xx-xx	xx<br>xx (xx-xx)<br>xx (xx)<br>xx<br>xx-xx	xx<br>xx (xx-xx)<br>xx (xx)<br>xx<br>xx-xx			xx<br>xx (xx-xx)<br>xx (xx)<br>xx<br>xx-xx	xx<br>xx (xx-xx)<br>xx (xx)<br>xx<br>xx-xx		xx<br>xx (xx-xx)<br>xx (xx)<br>xx<br>xx-xx	xx<br>xx (xx-xx)<br>xx (xx)<br>xx<br>xx-xx	xx<br>xx (xx-xx)<br>xx (xx)<br>xx<br>xx-xx			xx<br>xx (xx-xx)<br>xx (xx)<br>xx<br>xx-xx	xx<br>xx (xx-xx)<br>xx (xx)<br>xx<br>xx-xx
Mean Pocket Probing Depth (PPD) [mm]		xx<br>xx (xx-xx)<br>xx (xx)<br>xx<br>xx-xx	xx<br>xx (xx-xx)<br>xx (xx)<br>xx<br>xx-xx	xx<br>xx (xx-xx)<br>xx (xx)<br>xx<br>xx-xx			xx<br>xx (xx-xx)<br>xx (xx)<br>xx<br>xx-xx	xx<br>xx (xx-xx)<br>xx (xx)<br>xx<br>xx-xx		xx<br>xx (xx-xx)<br>xx (xx)<br>xx<br>xx-xx	xx<br>xx (xx-xx)<br>xx (xx)<br>xx<br>xx-xx	xx<br>xx (xx-xx)<br>xx (xx)<br>xx<br>xx-xx			xx<br>xx (xx-xx)<br>xx (xx)<br>xx<br>xx-xx	xx<br>xx (xx-xx)<br>xx (xx)<br>xx<br>xx-xx
Mean PPD [mm] of those sites with a baseline probing depth of ≥ 5mm		xx<br>xx (xx-xx)<br>xx (xx)<br>xx<br>xx-xx	xx<br>xx (xx-xx)<br>xx (xx)<br>xx<br>xx-xx	xx<br>xx (xx-xx)<br>xx (xx)<br>xx<br>xx-xx			xx<br>xx (xx-xx)<br>xx (xx)	xx<br>xx (xx-xx)<br>xx (xx)		xx<br>xx (xx-xx)<br>xx (xx)<br>xx<br>xx-xx	xx<br>xx (xx-xx)<br>xx (xx)<br>xx<br>xx-xx	xx<br>xx (xx-xx)<br>xx (xx)<br>xx<br>xx-xx			xx<br>xx (xx-xx)<br>xx (xx)	xx<br>xx (xx-xx)<br>xx (xx)

							xx<br>xx-xx	xx<br>xx-xx							xx<br>xx-xx	xx<br>xx-xx
Mean PPD [mm] of those sites with a baseline probing depth of ≥ 6mm		xx<br>xx (xx-xx)<br>xx (xx)<br>xx<br>xx-xx	xx<br>xx (xx-xx)<br>xx (xx)<br>xx<br>xx-xx	xx<br>xx (xx-xx)<br>xx (xx)<br>xx<br>xx-xx			xx<br>xx (xx-xx)<br>xx (xx)<br>xx<br>xx-xx	xx<br>xx (xx-xx)<br>xx (xx)<br>xx<br>xx-xx		xx<br>xx (xx-xx)<br>xx (xx)<br>xx<br>xx-xx	xx<br>xx (xx-xx)<br>xx (xx)<br>xx<br>xx-xx	xx<br>xx (xx-xx)<br>xx (xx)<br>xx<br>xx-xx			xx<br>xx (xx-xx)<br>xx (xx)<br>xx<br>xx-xx	xx<br>xx (xx-xx)<br>xx (xx)<br>xx<br>xx-xx
Mean PPD [mm] of those sites with a baseline probing depth of ≥ 7mm		xx<br>xx (xx-xx)<br>xx (xx)<br>xx<br>xx-xx	xx<br>xx (xx-xx)<br>xx (xx)<br>xx<br>xx-xx	xx<br>xx (xx-xx)<br>xx (xx)<br>xx<br>xx-xx			xx<br>xx (xx-xx)<br>xx (xx)<br>xx<br>xx-xx	xx<br>xx (xx-xx)<br>xx (xx)<br>xx<br>xx-xx		xx<br>xx (xx-xx)<br>xx (xx)<br>xx<br>xx-xx	xx<br>xx (xx-xx)<br>xx (xx)<br>xx<br>xx-xx	xx<br>xx (xx-xx)<br>xx (xx)<br>xx<br>xx-xx			xx<br>xx (xx-xx)<br>xx (xx)<br>xx<br>xx-xx	xx<br>xx (xx-xx)<br>xx (xx)<br>xx<br>xx-xx
Mean PPD [mm] of those sites with a baseline probing depth of ≤ 4mm		xx<br>xx (xx-xx)<br>xx (xx)<br>xx<br>xx-xx	xx<br>xx (xx-xx)<br>xx (xx)<br>xx<br>xx-xx	xx<br>xx (xx-xx)<br>xx (xx)<br>xx<br>xx-xx			xx<br>xx (xx-xx)<br>xx (xx)<br>xx<br>xx-xx	xx<br>xx (xx-xx)<br>xx (xx)<br>xx<br>xx-xx		xx<br>xx (xx-xx)<br>xx (xx)<br>xx<br>xx-xx	xx<br>xx (xx-xx)<br>xx (xx)<br>xx<br>xx-xx	xx<br>xx (xx-xx)<br>xx (xx)<br>xx<br>xx-xx			xx<br>xx (xx-xx)<br>xx (xx)<br>xx<br>xx-xx	xx<br>xx (xx-xx)<br>xx (xx)<br>xx<br>xx-xx
Proportion of sites with PPD>4mm (% [n=x])		% [n=x]	% [n=x]	% [n=x]			% [n=x]	% [n=x]		% [n=x]	% [n=x]	% [n=x]			% [n=x]	% [n=x]
Proportion of sites with PPD>6mm		% [n=x]	% [n=x]	% [n=x]			% [n=x]	% [n=x]		% [n=x]	% [n=x]	% [n=x]			% [n=x]	% [n=x]
Proportion of sites improving by >2mm.							% [n=x]	% [n=x]							% [n=x]	% [n=x]

Proportion of deep sites (baseline PPD >6mm) improving by >2mm.							%<br>[n=x]	%<br>[n=x]							%<br>[n=x]	%<br>[n=x]
Mean MGI [Modified Gingival Index]		xx<br>xx (xx-<br>xx)<br>xx (xx)<br>xx<br>xx-xx	xx<br>xx (xx-<br>xx)<br>xx (xx)<br>xx<br>xx-xx	xx<br>xx (xx-<br>xx)<br>xx (xx)<br>xx<br>xx-xx			xx<br>xx<br>(xx-<br>xx)<br>xx<br>(xx)<br>xx<br>xx-xx	xx<br>xx<br>(xx-<br>xx)<br>xx<br>(xx)<br>xx<br>xx-xx		xx<br>xx (xx-<br>xx)<br>xx (xx)<br>xx<br>xx-xx	xx<br>xx (xx-<br>xx)<br>xx (xx)<br>xx<br>xx-xx	xx<br>xx (xx-<br>xx)<br>xx (xx)<br>xx<br>xx-xx			xx<br>xx<br>(xx-<br>xx)<br>xx<br>(xx)<br>xx<br>xx-xx	xx<br>xx<br>(xx-<br>xx)<br>xx<br>(xx)<br>xx<br>xx-xx
Plaque score		xx<br>xx (xx-<br>xx)<br>xx (xx)<br>xx<br>xx-xx	xx<br>xx (xx-<br>xx)<br>xx (xx)<br>xx<br>xx-xx	xx<br>xx (xx-<br>xx)<br>xx (xx)<br>xx<br>xx-xx			xx<br>xx<br>(xx-<br>xx)<br>xx<br>(xx)<br>xx<br>xx-xx	xx<br>xx<br>(xx-<br>xx)<br>xx<br>(xx)<br>xx<br>xx-xx		xx<br>xx (xx-<br>xx)<br>xx (xx)<br>xx<br>xx-xx	xx<br>xx (xx-<br>xx)<br>xx (xx)<br>xx<br>xx-xx	xx<br>xx (xx-<br>xx)<br>xx (xx)<br>xx<br>xx-xx			xx<br>xx<br>(xx-<br>xx)<br>xx<br>(xx)<br>xx<br>xx-xx	xx<br>xx<br>(xx-<br>xx)<br>xx<br>(xx)<br>xx<br>xx-xx
Mean CAL [mm] [Clinical Attachment Level]		xx<br>xx (xx-<br>xx)<br>xx (xx)<br>xx<br>xx-xx	xx<br>xx (xx-<br>xx)<br>xx (xx)<br>xx<br>xx-xx	xx<br>xx (xx-<br>xx)<br>xx (xx)<br>xx<br>xx-xx			xx<br>xx<br>(xx-<br>xx)<br>xx<br>(xx)<br>xx<br>xx-xx	xx<br>xx<br>(xx-<br>xx)<br>xx<br>(xx)<br>xx<br>xx-xx		xx<br>xx (xx-<br>xx)<br>xx (xx)<br>xx<br>xx-xx	xx<br>xx (xx-<br>xx)<br>xx (xx)<br>xx<br>xx-xx	xx<br>xx (xx-<br>xx)<br>xx (xx)<br>xx<br>xx-xx			xx<br>xx<br>(xx-<br>xx)<br>xx<br>(xx)<br>xx<br>xx-xx	xx<br>xx<br>(xx-<br>xx)<br>xx<br>(xx)<br>xx<br>xx-xx
% BOP score [Bleeding On Probing]		xx<br>xx (xx-<br>xx)<br>xx (xx)<br>xx<br>xx-xx	xx<br>xx (xx-<br>xx)<br>xx (xx)<br>xx<br>xx-xx	xx<br>xx (xx-<br>xx)<br>xx (xx)<br>xx<br>xx-xx			xx<br>xx<br>(xx-<br>xx)<br>xx<br>(xx)<br>xx<br>xx-xx	xx<br>xx<br>(xx-<br>xx)<br>xx<br>(xx)<br>xx<br>xx-xx		xx<br>xx (xx-<br>xx)<br>xx (xx)<br>xx<br>xx-xx	xx<br>xx (xx-<br>xx)<br>xx (xx)<br>xx<br>xx-xx	xx<br>xx (xx-<br>xx)<br>xx (xx)<br>xx<br>xx-xx			xx<br>xx<br>(xx-<br>xx)<br>xx<br>(xx)<br>xx<br>xx-xx	xx<br>xx<br>(xx-<br>xx)<br>xx<br>(xx)<br>xx<br>xx-xx

Clinical Oral Dryness Score		xx<br>xx (xx-<br>xx)<br>xx (xx)<br>xx<br>xx-xx	xx<br>xx (xx-<br>xx)<br>xx (xx)<br>xx<br>xx-xx	xx<br>xx (xx-<br>xx)<br>xx (xx)<br>xx<br>xx-xx			xx<br>xx<br>(xx-<br>xx)<br>xx<br>(xx)<br>xx<br>xx-xx	xx<br>xx<br>(xx-<br>xx)<br>xx<br>(xx)<br>xx<br>xx-xx		xx<br>xx (xx-<br>xx)<br>xx (xx)<br>xx<br>xx-xx	xx<br>xx (xx-<br>xx)<br>xx (xx)<br>xx<br>xx-xx	xx<br>xx (xx-<br>xx)<br>xx (xx)<br>xx<br>xx-xx			xx<br>xx<br>(xx-<br>xx)<br>xx<br>(xx)<br>xx<br>xx-xx	xx<br>xx<br>(xx-<br>xx)<br>xx<br>(xx)<br>xx<br>xx-xx
PESA [mm^2] [Periodontal Epithelial Surface Area]		xx<br>xx (xx-<br>xx)<br>xx (xx)<br>xx<br>xx-xx	xx<br>xx (xx-<br>xx)<br>xx (xx)<br>xx<br>xx-xx	xx<br>xx (xx-<br>xx)<br>xx (xx)<br>xx<br>xx-xx			xx<br>xx<br>(xx-<br>xx)<br>xx<br>(xx)<br>xx<br>xx-xx	xx<br>xx<br>(xx-<br>xx)<br>xx<br>(xx)<br>xx<br>xx-xx		xx<br>xx (xx-<br>xx)<br>xx (xx)<br>xx<br>xx-xx	xx<br>xx (xx-<br>xx)<br>xx (xx)<br>xx<br>xx-xx	xx<br>xx (xx-<br>xx)<br>xx (xx)<br>xx<br>xx-xx			xx<br>xx<br>(xx-<br>xx)<br>xx<br>(xx)<br>xx<br>xx-xx	xx<br>xx<br>(xx-<br>xx)<br>xx<br>(xx)<br>xx<br>xx-xx
PISA [mm^2] [Periodontal Inflammed Surface Area]		xx<br>xx (xx-<br>xx)<br>xx (xx)<br>xx<br>xx-xx	xx<br>xx (xx-<br>xx)<br>xx (xx)<br>xx<br>xx-xx	xx<br>xx (xx-<br>xx)<br>xx (xx)<br>xx<br>xx-xx			xx<br>xx<br>(xx-<br>xx)<br>xx<br>(xx)<br>xx<br>xx-xx	xx<br>xx<br>(xx-<br>xx)<br>xx<br>(xx)<br>xx<br>xx-xx		xx<br>xx (xx-<br>xx)<br>xx (xx)<br>xx<br>xx-xx	xx<br>xx (xx-<br>xx)<br>xx (xx)<br>xx<br>xx-xx	xx<br>xx (xx-<br>xx)<br>xx (xx)<br>xx<br>xx-xx			xx<br>xx<br>(xx-<br>xx)<br>xx<br>(xx)<br>xx<br>xx-xx	xx<br>xx<br>(xx-<br>xx)<br>xx<br>(xx)<br>xx<br>xx-xx
OHQoL-UK		xx<br>xx (xx-<br>xx)<br>xx (xx)<br>xx<br>xx-xx	xx<br>xx (xx-<br>xx)<br>xx (xx)<br>xx<br>xx-xx	xx<br>xx (xx-<br>xx)<br>xx (xx)<br>xx<br>xx-xx				xx<br>xx<br>(xx-<br>xx)<br>xx<br>(xx)<br>xx<br>xx-xx		xx<br>xx (xx-<br>xx)<br>xx (xx)<br>xx<br>xx-xx	xx<br>xx (xx-<br>xx)<br>xx (xx)<br>xx<br>xx-xx	xx<br>xx (xx-<br>xx)<br>xx (xx)<br>xx<br>xx-xx				xx<br>xx<br>(xx-<br>xx)<br>xx<br>(xx)<br>xx<br>xx-xx
Interleukin (IL)-1β		xx<br>xx (xx-<br>xx)<br>xx (xx)<br>xx	xx<br>xx (xx-<br>xx)<br>xx (xx)<br>xx	xx<br>xx (xx-<br>xx)<br>xx (xx)<br>xx			xx<br>xx<br>(xx-<br>xx)	xx<br>xx<br>(xx-<br>xx)		xx<br>xx (xx-<br>xx)<br>xx (xx)<br>xx	xx<br>xx (xx-<br>xx)<br>xx (xx)<br>xx	xx<br>xx (xx-<br>xx)<br>xx (xx)<br>xx			xx<br>xx<br>(xx-<br>xx)	xx<br>xx<br>(xx-<br>xx)

		xx-xx	xx-xx	xx-xx			xx<br>(xx)<br>xx<br>xx-xx	xx<br>(xx)<br>xx<br>xx-xx		xx-xx	xx-xx	xx-xx			xx<br>(xx)<br>xx<br>xx-xx	xx<br>(xx)<br>xx<br>xx-xx
Matrix metalloproteinase 8 (MMP-8)		xx<br>xx (xx-xx)<br>xx (xx)<br>xx<br>xx-xx	xx<br>xx (xx-xx)<br>xx (xx)<br>xx<br>xx-xx	xx<br>xx (xx-xx)<br>xx (xx)<br>xx<br>xx-xx			xx<br>xx<br>(xx-xx)<br>xx<br>(xx)<br>xx<br>xx-xx	xx<br>xx<br>(xx-xx)<br>xx<br>(xx)<br>xx<br>xx-xx		xx<br>xx (xx-xx)<br>xx (xx)<br>xx<br>xx-xx	xx<br>xx (xx-xx)<br>xx (xx)<br>xx<br>xx-xx	xx<br>xx (xx-xx)<br>xx (xx)<br>xx<br>xx-xx			xx<br>xx<br>(xx-xx)<br>xx<br>(xx)<br>xx<br>xx-xx	xx<br>xx<br>(xx-xx)<br>xx<br>(xx)<br>xx<br>xx-xx
Microbiological outcome measure	Exploratory analysis of the presence and proportions of bacterial species present will be quantified.															

Table 14.3: Smoking related outcome measures

Outcome<br><br>Min<br>Median (LQ-UQ)<br>Mean (SD)<br>Max<br>95% CI<br><br>*Unless otherwise detailed*	n	Control Group, n=								n	Intervention Group, n=							
		Baseline (all)	Baseline of those reaching 4-week visit	Baseline of those reaching 3-month visit	Baseline of those reaching 6-month visit	Quit date	4-weeks	3-month	6-month		Baseline	Baseline of those reaching 4-week visit	Baseline of those reaching 3-month visit	Baseline of those reaching 6-month visit	Quit date	4-weeks	3-month	6-month
FTND		xx<br>xx (xx-xx)<br>xx (xx)<br>xx<br>xx-xx	xx<br>xx (xx-xx)<br>xx (xx)<br>xx<br>xx-xx	xx<br>xx (xx-xx)<br>xx (xx)<br>xx<br>xx-xx	xx<br>xx (xx-xx)<br>xx (xx)<br>xx<br>xx-xx	xx<br>xx (xx-xx)<br>xx (xx)<br>xx<br>xx-xx	xx<br>xx (xx-xx)<br>xx (xx)<br>xx<br>xx-xx	xx<br>xx (xx-xx)<br>xx (xx)<br>xx<br>xx-xx	xx<br>xx (xx-xx)<br>xx (xx)<br>xx<br>xx-xx		xx<br>xx (xx-xx)<br>xx (xx)<br>xx<br>xx-xx	xx<br>xx (xx-xx)<br>xx (xx)<br>xx<br>xx-xx	xx<br>xx (xx-xx)<br>xx (xx)<br>xx<br>xx-xx	xx<br>xx (xx-xx)<br>xx (xx)<br>xx<br>xx-xx	xx<br>xx (xx-xx)<br>xx (xx)<br>xx<br>xx-xx	xx<br>xx (xx-xx)<br>xx (xx)<br>xx<br>xx-xx	xx<br>xx (xx-xx)<br>xx (xx)<br>xx<br>xx-xx	xx<br>xx (xx-xx)<br>xx (xx)<br>xx<br>xx-xx
MPPS		xx<br>xx (xx-xx)<br>xx (xx)<br>xx<br>xx-xx	xx<br>xx (xx-xx)<br>xx (xx)<br>xx<br>xx-xx	xx<br>xx (xx-xx)<br>xx (xx)<br>xx<br>xx-xx	xx<br>xx (xx-xx)<br>xx (xx)<br>xx<br>xx-xx	xx<br>xx (xx-xx)<br>xx (xx)<br>xx<br>xx-xx	xx<br>xx (xx-xx)<br>xx (xx)<br>xx<br>xx-xx	xx<br>xx (xx-xx)<br>xx (xx)<br>xx<br>xx-xx	xx<br>xx (xx-xx)<br>xx (xx)<br>xx<br>xx-xx		xx<br>xx (xx-xx)<br>xx (xx)<br>xx<br>xx-xx	xx<br>xx (xx-xx)<br>xx (xx)<br>xx<br>xx-xx	xx<br>xx (xx-xx)<br>xx (xx)<br>xx<br>xx-xx	xx<br>xx (xx-xx)<br>xx (xx)<br>xx<br>xx-xx	xx<br>xx (xx-xx)<br>xx (xx)<br>xx<br>xx-xx	xx<br>xx (xx-xx)<br>xx (xx)<br>xx<br>xx-xx	xx<br>xx (xx-xx)<br>xx (xx)<br>xx<br>xx-xx	xx<br>xx (xx-xx)<br>xx (xx)<br>xx<br>xx-xx
MPPS (M)		xx	xx	xx	xx	xx	xx	xx	xx		xx	xx	xx	xx	xx	xx	xx	xx

		xx (xx-xx)<br>xx (xx)<br>xx<br>xx-xx	xx (xx-xx)<br>xx (xx)<br>xx<br>xx-xx	xx (xx-xx)<br>xx (xx)<br>xx<br>xx-xx	xx (xx-xx)<br>xx (xx)<br>xx<br>xx-xx	xx (xx-xx)<br>xx (xx)<br>xx<br>xx-xx	xx (xx-xx)<br>xx (xx)<br>xx<br>xx-xx	xx (xx-xx)<br>xx (xx)<br>xx<br>xx-xx	xx (xx-xx)<br>xx (xx)<br>xx<br>xx-xx		xx (xx-xx)<br>xx (xx)<br>xx<br>xx-xx	xx (xx-xx)<br>xx (xx)<br>xx<br>xx-xx	xx (xx-xx)<br>xx (xx)<br>xx<br>xx-xx	xx (xx-xx)<br>xx (xx)<br>xx<br>xx-xx	xx (xx-xx)<br>xx (xx)<br>xx<br>xx-xx	xx (xx-xx)<br>xx (xx)<br>xx<br>xx-xx	xx (xx-xx)<br>xx (xx)<br>xx<br>xx-xx	xx (xx-xx)<br>xx (xx)<br>xx<br>xx-xx
MPSS (C)		xx<br>xx (xx-xx)<br>xx (xx)<br>xx<br>xx-xx	xx<br>xx (xx-xx)<br>xx (xx)<br>xx<br>xx-xx	xx<br>xx (xx-xx)<br>xx (xx)<br>xx<br>xx-xx	xx<br>xx (xx-xx)<br>xx (xx)<br>xx<br>xx-xx	xx<br>xx (xx-xx)<br>xx (xx)<br>xx<br>xx-xx	xx<br>xx (xx-xx)<br>xx (xx)<br>xx<br>xx-xx	xx<br>xx (xx-xx)<br>xx (xx)<br>xx<br>xx-xx	xx<br>xx (xx-xx)<br>xx (xx)<br>xx<br>xx-xx		xx<br>xx (xx-xx)<br>xx (xx)<br>xx<br>xx-xx	xx<br>xx (xx-xx)<br>xx (xx)<br>xx<br>xx-xx	xx<br>xx (xx-xx)<br>xx (xx)<br>xx<br>xx-xx	xx<br>xx (xx-xx)<br>xx (xx)<br>xx<br>xx-xx	xx<br>xx (xx-xx)<br>xx (xx)<br>xx<br>xx-xx	xx<br>xx (xx-xx)<br>xx (xx)<br>xx<br>xx-xx	xx<br>xx (xx-xx)<br>xx (xx)<br>xx<br>xx-xx	xx<br>xx (xx-xx)<br>xx (xx)<br>xx<br>xx-xx
MPSS (P)		xx<br>xx (xx-xx)<br>xx (xx)<br>xx<br>xx-xx	xx<br>xx (xx-xx)<br>xx (xx)<br>xx<br>xx-xx	xx<br>xx (xx-xx)<br>xx (xx)<br>xx<br>xx-xx	xx<br>xx (xx-xx)<br>xx (xx)<br>xx<br>xx-xx	xx<br>xx (xx-xx)<br>xx (xx)<br>xx<br>xx-xx	xx<br>xx (xx-xx)<br>xx (xx)<br>xx<br>xx-xx	xx<br>xx (xx-xx)<br>xx (xx)<br>xx<br>xx-xx	xx<br>xx (xx-xx)<br>xx (xx)<br>xx<br>xx-xx		xx<br>xx (xx-xx)<br>xx (xx)<br>xx<br>xx-xx	xx<br>xx (xx-xx)<br>xx (xx)<br>xx<br>xx-xx	xx<br>xx (xx-xx)<br>xx (xx)<br>xx<br>xx-xx	xx<br>xx (xx-xx)<br>xx (xx)<br>xx<br>xx-xx	xx<br>xx (xx-xx)<br>xx (xx)<br>xx<br>xx-xx	xx<br>xx (xx-xx)<br>xx (xx)<br>xx<br>xx-xx	xx<br>xx (xx-xx)<br>xx (xx)<br>xx<br>xx-xx	xx<br>xx (xx-xx)<br>xx (xx)<br>xx<br>xx-xx
eCO		xx<br>xx (xx-xx)<br>xx (xx)<br>xx<br>xx-xx	xx<br>xx (xx-xx)<br>xx (xx)<br>xx<br>xx-xx	xx<br>xx (xx-xx)<br>xx (xx)<br>xx<br>xx-xx	xx<br>xx (xx-xx)<br>xx (xx)<br>xx<br>xx-xx	xx<br>xx (xx-xx)<br>xx (xx)<br>xx<br>xx-xx	xx<br>xx (xx-xx)<br>xx (xx)<br>xx<br>xx-xx	xx<br>xx (xx-xx)<br>xx (xx)<br>xx<br>xx-xx	xx<br>xx (xx-xx)<br>xx (xx)<br>xx<br>xx-xx		xx<br>xx (xx-xx)<br>xx (xx)<br>xx<br>xx-xx	xx<br>xx (xx-xx)<br>xx (xx)<br>xx<br>xx-xx	xx<br>xx (xx-xx)<br>xx (xx)<br>xx<br>xx-xx	xx<br>xx (xx-xx)<br>xx (xx)<br>xx<br>xx-xx	xx<br>xx (xx-xx)<br>xx (xx)<br>xx<br>xx-xx	xx<br>xx (xx-xx)<br>xx (xx)<br>xx<br>xx-xx	xx<br>xx (xx-xx)<br>xx (xx)<br>xx<br>xx-xx	xx<br>xx (xx-xx)<br>xx (xx)<br>xx<br>xx-xx
Salivary Cotinine		xx	xx	xx	xx	xx	xx	xx	xx		xx	xx	xx	xx	xx	xx	xx	xx

(SC)<br>[ng/ml]		xx (xx-xx)<br>xx (xx)<br>xx<br>xx-xx	xx (xx-xx)<br>xx (xx)<br>xx<br>xx-xx	xx (xx-xx)<br>xx (xx)<br>xx<br>xx-xx	xx (xx-xx)<br>xx (xx)<br>xx<br>xx-xx	xx (xx-xx)<br>xx (xx)<br>xx<br>xx-xx	xx (xx-xx)<br>xx (xx)<br>xx<br>xx-xx	xx (xx-xx)<br>xx (xx)<br>xx<br>xx-xx	xx (xx-xx)<br>xx (xx)<br>xx<br>xx-xx		xx (xx-xx)<br>xx (xx)<br>xx<br>xx-xx	xx (xx-xx)<br>xx (xx)<br>xx<br>xx-xx	xx (xx-xx)<br>xx (xx)<br>xx<br>xx-xx	xx (xx-xx)<br>xx (xx)<br>xx<br>xx-xx	xx (xx-xx)<br>xx (xx)<br>xx<br>xx-xx	xx (xx-xx)<br>xx (xx)<br>xx<br>xx-xx	xx (xx-xx)<br>xx (xx)<br>xx<br>xx-xx	xx (xx-xx)<br>xx (xx)<br>xx<br>xx-xx
SC: proportion of participants with readings below 15ng/ml.[a]		%<br>[n=x]	%<br>[n=x]	%<br>[n=x]	%<br>[n=x]	%<br>[n=x]	%<br>[n=x]	%<br>[n=x]	%<br>[n=x]		%<br>[n=x]	%<br>[n=x]	%<br>[n=x]	%<br>[n=x]	%<br>[n=x]	%<br>[n=x]	%<br>[n=x]	%<br>[n=x]
Salivary Anabasine (SA)		xx<br>xx (xx-xx)<br>xx (xx)<br>xx<br>xx-xx	xx<br>xx (xx-xx)<br>xx (xx)<br>xx<br>xx-xx	xx<br>xx (xx-xx)<br>xx (xx)<br>xx<br>xx-xx	xx<br>xx (xx-xx)<br>xx (xx)<br>xx<br>xx-xx	xx<br>xx (xx-xx)<br>xx (xx)<br>xx<br>xx-xx	xx<br>xx (xx-xx)<br>xx (xx)<br>xx<br>xx-xx	xx<br>xx (xx-xx)<br>xx (xx)<br>xx<br>xx-xx	xx<br>xx (xx-xx)<br>xx (xx)<br>xx<br>xx-xx		xx<br>xx (xx-xx)<br>xx (xx)<br>xx<br>xx-xx	xx<br>xx (xx-xx)<br>xx (xx)<br>xx<br>xx-xx	xx<br>xx (xx-xx)<br>xx (xx)<br>xx<br>xx-xx	xx<br>xx (xx-xx)<br>xx (xx)<br>xx<br>xx-xx	xx<br>xx (xx-xx)<br>xx (xx)<br>xx<br>xx-xx	xx<br>xx (xx-xx)<br>xx (xx)<br>xx<br>xx-xx	xx<br>xx (xx-xx)<br>xx (xx)<br>xx<br>xx-xx	xx<br>xx (xx-xx)<br>xx (xx)<br>xx<br>xx-xx
SA: Proportion of participants with readings below 0.1ng/ml.[b]		%<br>[n=x]	%<br>[n=x]	%<br>[n=x]	%<br>[n=x]	%<br>[n=x]	%<br>[n=x]	%<br>[n=x]	%<br>[n=x]		%<br>[n=x]	%<br>[n=x]	%<br>[n=x]	%<br>[n=x]	%<br>[n=x]	%<br>[n=x]	%<br>[n=x]	%<br>[n=x]
Self-reported quitters of						%<br>[n=x]	%<br>[n=x]	%<br>[n=x]	%<br>[n=x]						%<br>[n=x]	%<br>[n=x]	%<br>[n=x]	%<br>[n=x]

burnt tobacco[c]																		
eCO verified self-reported quitter						% [n=x]	% [n=x]	% [n=x]	% [n=x]						% [n=x]	% [n=x]	% [n=x]	% [n=x]
SC/SA verified self-reported quitter						% [n=x]	% [n=x]	% [n=x]	% [n=x]						% [n=x]	% [n=x]	% [n=x]	% [n=x]
eCO and SC/SA verified self-reported quitter						% [n=x]	% [n=x]	% [n=x]	% [n=x]						% [n=x]	% [n=x]	% [n=x]	% [n=x]
Russell Standard 6-month quitter (RS6)[d]									% [n=x]									% [n=x]
Cumulative self-reported burnt tobacco use									xx									xx
Cumulative self-reported EC use (No. of days using EC)									xx									xx

a- Salivary Cotinine measurements of below 15ng/ml signifies a non-user of burnt tobacco or nicotine products.
b- Salivary Anabasine measurements of below 0.1ng/ml signifies a non-user of burnt tobacco [when participant is using NRT/e-cigarettes]
c- This measure will cover the following Russell Standard time points: SR4WQ (self-reported 4-week quitter) and SR6MQ (self-reported 6-month quitter)
d- There are five criteria: 1) six-months follow-up from quit date, 2) self-reported smoking abstinence over whole follow-up period allowing up to five cigarettes in total, 3) biochemical validation of abstinence, 4) 'intention-to-treat' analysis with participants counted as smokers if smoking status can't be determined [unless died or moved to an untraceable address], 5) following up 'protocol violators' and using true smoking status in analysis. [N.B. The Russell Standard has a sixth criteria, blind follow-up, that was not possible in this study due to the limitations of a doctoral research project].

No interpretation will be made of these change data summaries nor of any apparent change within allocation groups given the fact that this cannot be meaningfully interpreted without further analysis.(15, 16)

Plots of outcome measures

Box and spaghetti plots will be created for each of the following, by randomised group:

- Mean PPD (mm)
- Proportion of sites with PPD>4mm
- Proportion of sites with PPD>6mm
- Proportion of sites improving by >2mm.
- Proportion of deep sites (baseline PPD >6mm) improving by >2mm.
- MGI
- PI
- CAL
- BOP
- CODS
- PESA
- PISA
- OHQoL-UK
- FTND
- MPPS
- eCO
- Salivary Cotinine (SC)
- SC: proportion of participants with readings below 15ng/ml.
- Salivary Anabasine (SA)
- SA: Proportion of participants with readings below <0.1ng/ml.
- Interleukin (IL)- 1β
- Matrix metalloproteinase 8 (MMP-8)
- Self-reported quitters of burnt tobacco
- eCO verified self-reported quitter
- SC/SA verified self-reported quitter
- eCO and SC/SA verified self-reported quitter
- Russell Standard 6-month quitter (RS6)

Figure 14.1: Example plot: Mean PPD (mm)

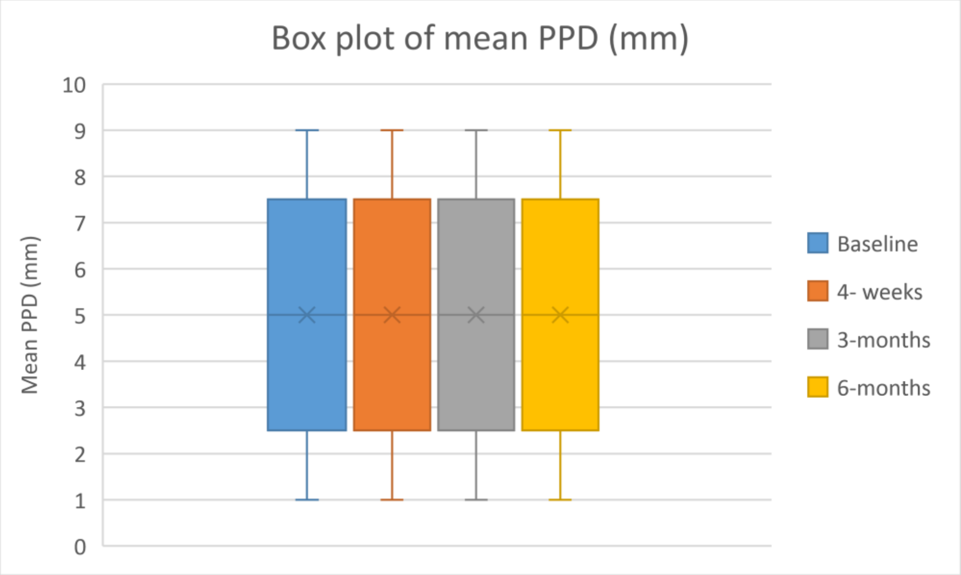

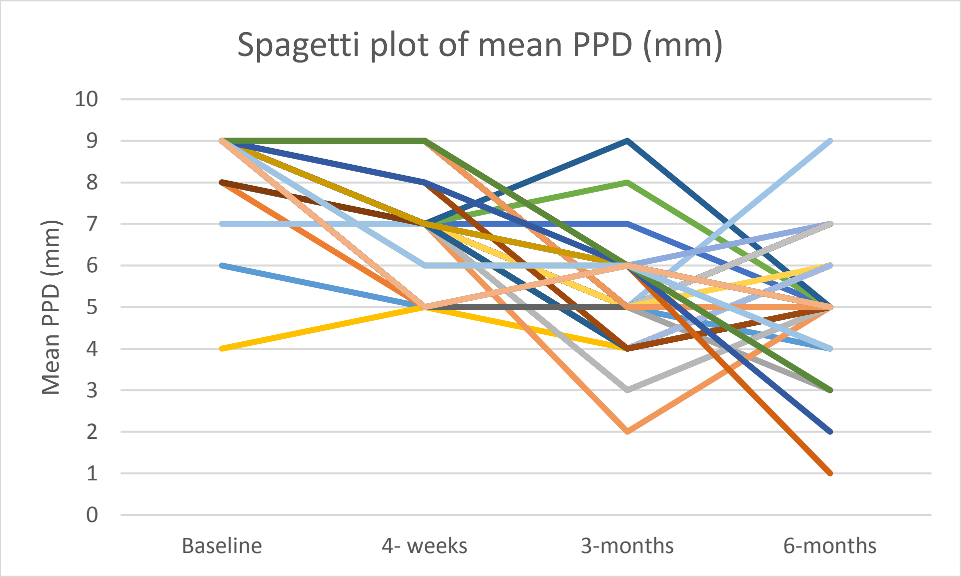

Table 14.4: Change in outcome measures between visits

Outcome<br><br>Min<br>Median (LQ-UQ)<br>Mean (SD)<br>Max<br>95% CI<br><br>*Unless otherwise detailed*	Control Group, n=			Intervention Group, n=		
	Change from baseline to 4-weeks	Change from baseline to 3-months	Change from baseline to 6-months	Change from baseline to 4-weeks	Change from baseline to 3-months	Change from baseline to 6-months
Mean PPD [mm]						
Mean PPD [mm] of those sites with a baseline probing depth of ≥ 5mm						
Mean PPD [mm] of those sites with a baseline probing depth of ≥ 6mm						
Mean PPD [mm] of those sites with a baseline probing depth of ≥ 7mm						
Mean PPD [mm] of those sites with a baseline probing depth of ≤ 4mm						
Proportion of sites with PPD>4mm (% [n=x])						
Proportion of sites with PPD>6mm						
Proportion of sites improving by >2mm.						
Proportion of deep sites (baseline PPD >6mm) improving by >2mm.						
Mean MGI [Modified Gingival Index]						
Plaque score						
Mean CAL [mm] [Clinical Attachment Level]						
% BOP score						

[Bleeding On Probing]						
Clinical Oral Dryness Score						
PESA [mm^2]<br>[Periodontal Epithelial Surface Area]						
PISA [mm^2]<br>[Periodontal Inflammed Surface Area]						
OHQoL-UK						
FTND						
MPPS						
MPPS (M)						
MPSS (C)						
MPSS (P)						
eCO						
Salivary Cotinine (SC)						
SC: proportion of participants with readings below 15ng/ml.						
Salivary Anabasine (SA)						
SA: Proportion of participants with readings below <0.1ng/ml.						
Interleukin (IL)- 1β						
Matrix metalloproteinase 8 (MMP-8)						

15 Treatment comparisons

The study has not been designed to make treatment comparisons or draw inferences and as such no formal statistical testing will be performed. However, for smoking abstinence at 4-weeks and 6-months, a comparison of the difference in the percentage achieving this between treatment groups will be reported with a 95% confidence interval for the difference.

16 References

1. Moher D, Hopewell S, Schulz KF, Montori V, Gøtzsche PC, Devereaux PJ, et al. CONSORT 2010 Explanation and Elaboration: updated guidelines for reporting parallel group randomised trials. BMJ. 2010;340.

2. Altman DG. Comparability of Randomised Groups. Journal of the Royal Statistical Society Series D (The Statistician). 1985;34(1):125-36.

3. Roberts C, Torgerson DJ. Baseline imbalance in randomised controlled trials. BMJ. 1999;319(7203):185.

4. Eldridge SM, Chan CL, Campbell MJ, Bond CM, Hopewell S, Thabane L, et al. CONSORT 2010 statement: extension to randomised pilot and feasibility trials. BMJ. 2016;355:i5239.

5. West R, Hajek P, Stead L, Stapleton J. Outcome criteria in smoking cessation trials: proposal for a common standard. Addiction. 2005;100(3):299-303.

6. Hartmann-Boyce J, McRobbie H, Bullen C, Begh R, Stead LF, Hajek P. Electronic cigarettes for smoking cessation. The Cochrane database of systematic reviews. 2016;9:Cd010216.

7. Peyre H, Leplege A, Coste J. Missing data methods for dealing with missing items in quality of life questionnaires. A comparison by simulation of personal mean score, full information maximum likelihood, multiple imputation, and hot deck techniques applied to the SF-36 in the French 2003 decennial health survey. Qual Life Res. 2011;20(2):287-300.

8. Fayers PM, Curran D, Machin D. Incomplete quality of life data in randomized trials: missing items. Stat Med. 1998;17(5-7):679-96.

9. Fairclough DL, Cella DF. Functional Assessment of Cancer Therapy (FACT-G): non-response to individual questions. Qual Life Res. 1996;5(3):321-9.

10. Irani FC, Wassall RR, Preshaw PM. Impact of periodontal status on oral health-related quality of life in patients with and without type 2 diabetes. J Dent. 2015;43(5):506-11.

11. Durham J, Fraser HM, McCracken GI, Stone KM, John MT, Preshaw PM. Impact of periodontitis on oral health-related quality of life. J Dent. 2013;41(4):370-6.

12. Steele JG, Sanders AE, Slade GD, Allen PF, Lahti S, Nuttall N, et al. How do age and tooth loss affect oral health impacts and quality of life? A study comparing two national samples. Community Dent Oral Epidemiol. 2004;32(2):107-14.

13. Lancaster GA, Dodd S, Williamson PR. Design and analysis of pilot studies: recommendations for good practice. J Eval Clin Pract. 2004;10(2):307-12.

14. Thabane L, Ma J, Chu R, Cheng J, Ismaila A, Rios LP, et al. A tutorial on pilot studies: the what, why and how. BMC Med Res Methodol. 2010;10:1.

15. Bland JM, Altman DG. Comparisons within randomised groups can be very misleading. BMJ. 2011;342.

16. Vickers AJ, Altman DG. Analysing controlled trials with baseline and follow up measurements. BMJ. 2001;323(7321):1123.

17 Appendices

Table 17.1: Chronological listing of protocol deviations

Participant Study ID	Treatment allocation	Date	Reason

Table 17.2: Listing of Adverse Events (AE) by participant number

Sequence No.	Study ID	Study Arm	Adverse Event	Start Date dd/mm/yyyy	End Date dd/mm/yyyy	Serious*	Severity*	Relationship to Study*	Action Taken*	Other Action Comment	Outcome*	Outcome Comment
