# Supplementary material for: A feasibility study with embedded pilot randomised controlled trial and process evaluation of electronic cigarettes for smoking cessation in patients with periodontitis
Source: Pilot Feasibility Stud. 2019 Jun 4;5:74. doi: 10.1186/s40814-019-0451-4 (PMC6547559; doi:10.1186/s40814-019-0451-4)
Supplement: Supplementary file 10 — Recruitment source of study participants. Detailed breakdowns of the participant recruitment sources. (DOCX 12 kb) [file 40814_2019_451_MOESM10_ESM.docx]

Additional file 10. Recruitment source of study participants

| **Recruitment source** | **Potentially eligible participants [n (%)]** | **Consented and randomised [n (%)]** | **Consent rate**  **[% (95%CI)]** |
| --- | --- | --- | --- |
| Periodontal new patient clinic | 29 (24%) | 24 (30%) | 83% (65%-92%) |
| Other new patient clinics | 9 (8%) | 9 (11%) | 100% (70%-100%) |
| Dental Emergency Clinic (DEC) | 43 (36%) | 29 (36%) | 67% (52%-80%) |
| Participant Identification Centres (PICs) | 35 (29%) | 15 (19%) | 43% (67%-85%) |
| Other e.g. Undergraduate student clinic | 3 (3%) | 3 (4%) | 100% (44%-100%) |
| Total | 119 (100%) | 80 (100%) | 67% (58%-75%) |
