# Supplementary material for: A feasibility study with embedded pilot randomised controlled trial and process evaluation of electronic cigarettes for smoking cessation in patients with periodontitis
Source: Pilot Feasibility Stud. 2019 Jun 4;5:74. doi: 10.1186/s40814-019-0451-4 (PMC6547559; doi:10.1186/s40814-019-0451-4)
Supplement: Supplementary file 11 — Eligibility outcomes. Eligibility outcomes from one of the recruitment sources (periodontal new patient clinic). (DOCX 12 kb) [file 40814_2019_451_MOESM11_ESM.docx]

Additional file 11. Eligibility outcomes from the periodontal new patient clinic

| **Eligibility outcome** | **No. of patients** |
| --- | --- |
|  |  |
| Eligible | 29 (7.4%) |
| Ineligible | 362 (92.6%) |
|  |  |
| Reason for ineligibility: |  |
| Non-smoker | 334 (85.4%) |
| Smoker but smokes <10 factory-made cigarettes/day | 8 (2.0%) |
| Using an e-cigarette (3 or more days use in the last 30) | 8 (2.0%) |
| <16 natural teeth^#^ | 4 (1.0%) |
| Declined participation in research study* | 4 (1.0%) |
| Periodontitis does not meet criteria | 2 (0.5%) |
| Significant medical history | 1 (0.3%) |
| Currently undergoing extensive dental, orthodontic, implant or peri-implant treatment | 1 (0.3%) |
| Pregnant/nursing | 0 |
| Periodontal treatment (other than scale and polish) in last 6 months | 0 |

# This was 20 teeth for the first part of the study until the protocol change after seven months.

*Declined to take part in research study at the initial contact by the usual care team.
