# Supplementary material for: A feasibility study with embedded pilot randomised controlled trial and process evaluation of electronic cigarettes for smoking cessation in patients with periodontitis
Source: Pilot Feasibility Stud. 2019 Jun 4;5:74. doi: 10.1186/s40814-019-0451-4 (PMC6547559; doi:10.1186/s40814-019-0451-4)
Supplement: Supplementary file 12 — Participant follow-up by randomisation group. Detailed follow-up data by randomisation group, including compliance with target visit window. (DOCX 13 kb) [file 40814_2019_451_MOESM12_ESM.docx]

**Additional file 12. Participant follow-up by randomisation group**

|  | | **Control**  **n=40** | **Intervention**  **n=40** | **Total**  **n=80** |
| --- | --- | --- | --- | --- |
| Visit 1 (Baseline) | | 40 (100%) | 40 (100%) | 80 (100%) |
| Visit 4  (4 weeks [minus 3 days or plus 14 days]) | Complied with study window | 29 (73%) | 29 (73%) | 58 (73%) |
|  | Outwith study window | 6 (15%) | 3 (8%) | 9 (11%) |
|  | Did not attend | 5 (13%) | 8 (20%) | 13 (16%) |
| Visit 5  (3 months [minus 15 days or plus 28 days]) | Complied with study window | 28 (70%) | 31 (78%) | 59 (74%) |
|  | Outwith study window | 3 (8%) | 2 (5%) | 5 (6%) |
|  | Did not attend | 9 (23%) | 7 (18%) | 16 (20%) |
| Visit 6  (6 months [minus 15 days or plus 28 days]) | Complied with study window | 27 (68%) | 24 (60%) | 51 (64%) |
|  | Outwith study window | 2 (5%) | 5 (13%) | 7 (9%) |
|  | Did not attend | 11 (28%) | 11 (28%) | 1. 28%) |
