# Supplementary material for: A feasibility study with embedded pilot randomised controlled trial and process evaluation of electronic cigarettes for smoking cessation in patients with periodontitis
Source: Pilot Feasibility Stud. 2019 Jun 4;5:74. doi: 10.1186/s40814-019-0451-4 (PMC6547559; doi:10.1186/s40814-019-0451-4)
Supplement: Supplementary file 13 — Baseline characteristics of those lost to follow-up. Baseline characteristics of those lost to follow up by randomisation group. (DOCX 21 kb) [file 40814_2019_451_MOESM13_ESM.docx]

| **Baseline variable** | **All participants** | | | **Participant lost to follow-up** | | | **Remaining participants** | |
| --- | --- | --- | --- | --- | --- | --- | --- | --- |
|  | **Control group**  **n=40** | **Intervention group**  **n=40** | **Control group**  **n=11** | | **Intervention group**  **n=11** | **Control group**  **n=29** | | **Intervention group**  **n=29** |
| Sex, n (%) |  |  |  | |  |  | |  |
| Female | 20 (50%) | 22 (55%) | 6 (55%) | | 6 (55%) | 14 (48%) | | 16 (55%) |
| Male | 20 (50%) | 18 (45%) | 5 (46%) | | 5 (46%) | 15 (52%) | | 13 (45%) |
| Ethnicity, n (%) |  |  |  | |  |  | |  |
| White (British, Irish, other White) | 36 (90%) | 39 (97.5%) | 10 (91%) | | 11 (100%) | 26 (90%) | | 28 (97%) |
| Asian or Asian British (Indian, Pakistani, Bangladeshi, other Asian) | 4 (10%) | 1 (2.5%) | 1 (9%) | | 0 | 3 (10%) | | 1 (3%) |
| Occupation, n (%) |  |  |  | |  |  | |  |
| Working in a routine or manual occupation | 9 (22.5%) | 11 (27.5%) | 3 (27%) | | 6 (55%) | 6 (21%) | | 5 (17%) |
| Working in an intermediate occupation | 9 (22.5%) | 13 (32.5%) | 2 (18%) | | 1 (9%) | 7 (24%) | | 12 (17%) |
| Working in a managerial or professional occupation | 9 (22.5%) | 9 (22.5%) | 3 (27%) | | 1 (9%) | 9 (31%) | | 8 (28%) |
| Unemployed/not working for a year or more | 6 (15%) | 2 (5%) | 2 (18%) | | 1 (9%) | 3 (10%) | | 1 (3%) |
| Full time student | 0 | 1 (2.5%) | 0 | | 1 (9%) | 0 | | 0 |
| Retired | 1 (2.5%) | 4 (10%) | 0 | | 1 (9%) | 1 (3%) | | 3 (10%) |
| Sick/Disabled/Unable to return to work | 4 (10%) | 0 | 2 (18%) | | 0 | 2 (7%) | | 0 |
| Home carer (unpaid) | 2 (5%) | 0 | 1 (9%) | | 0 | 1 (3%) | | 0 |
| Age (years), mean (SD) | 44.6 (9.5) | 44.0 (11.8) | 47.7 (6.4) | | 37.1 (12.8) | 43.3 (10.3) | | 46.6 (10.5) |
| Number of cigarettes/day (any), mean (SD) | 17.5 (6.9) | 17.4 (6.4) | 18.4 (4.8) | | 21.4 (9.0) | 17.1 (7.6) | | 15.9 (4.4) |
| Number of factory cigarettes/day, mean (SD) | 16.6 (7.2), n=33 | 14.8 (4.4), n=26 | 18.2 (5.1), n=10 | | 15.8 (4.9), n=6 | 15.9 (7.9), n=23 | | 14.5 (4.2), n=20 |
| Number of hand-rolled cigarettes/day, mean (SD) | 19.0 (7.1), n=8 | 22.1 (7.0), n=14 | 20.0, n=1 | | 28.0 (8.4), n=5 | 18.9 (7.7), n=7 | | 18.9 (3.3), n=9 |
| Age started smoking, mean (SD) | 16.0 (2.8) | 15.3 (3.2) | 16.1 (2.7) | | 13.5 (2.1) | 16.0 (2.9) | | 16.0 (3.3) |
| eCO (ppm), mean (SD) | 18.1 (10.0) | 23.0 (12.2) | 20.6 (8.1) | | 25.5 (10.7) | 17.1 (10.4) | | 22.0 (12.8) |
| FTND, mean (SD) | 5.0 (2.4) | 5.0 (1.8) | 6.0 (2.1) | | 6.0 (2.1) | 4.6 (2.5) | | 4.6 (1.6) |
| MPSS, mean (SD) | 22.8 (7.0) | 22.8 (5.9) | 22.8 (5.6) | | 25.5 (7.6) | 22.8 (7.5) | | 21.8 (4.9) |
| SC (ng/ml), mean (SD) | 303.4 (128.3) | 342.6 (138.1) | 367.7 (97.6) | | 385.6 (110.9) | 277.2 (131.5) | | 326.3 (145.5) |
| SA (ng/ml), mean (SD) | 1.1 (1.4) | 1.2 (1.2) | 1.9 (2.1) | | 1.0 (0.5) | 0.8 (0.8) | | 1.2 (1.3) |
| Number of teeth (excluding 3^rd^ molars), mean (SD) | 24.0 (3.6) | 23.8 (4.0) | 21.1 (3.6) | | 23.6 (3.9) | 25.0 (3.0) | | 23.9 (4.1) |
| Mean PI, mean (SD) | 1.1(0.7) | 0.8 (0.6) | 1.4 (0.6) | | 1.0 (0.5) | 1.0 (0.6) | | 0.7 (0.7) |
| % BOP score, mean (SD) | 23.9 (18.3) | 16.5 (13.4) | 20.4 (18.3) | | 15.2 (15.7) | 25.2 (18.5) | | 17.0 (12.6) |
| Mean MGI, mean (SD) | 2.5 (0.5) | 2.5 (0.4) | 2.6 (0.4) | | 2.7 (0.4) | 2.5 (0.5) | | 2.3 (0.4) |
| Mean PPD (mm), mean (SD) | 4.1 (0.7) | 3.9 (0.7) | 4.4 (0.4) | | 4.1 (0.6) | 4.0 (0.8) | | 3.8 (0.7) |
| Mean CAL (mm), mean (SD) | 5.2 (1.4) | 5.1 (1.3) | 6.3 (1.2) | | 5.1 (1.2) | 4.8 (1.2) | | 5.1 (1.3) |
| PESA (mm^2^), mean (SD) | 2134.1 (666.7) | 2013.8 (644.4) | 1961.1 (547.7) | | 2071.1 (509.8) | 2200.0 (704.1) | | 1992.1 (695.5) |
| PISA (mm^2^), mean (SD) | 634.5 (629.9) | 386.7 (346.2) | 497.4 (590.8) | | 375.8 (415.9) | 686.6 (646.4) | | 390.8 (324.1) |
| No. of sites with PPD ≥5 mm, mean (SD) | 60.5 (30.9) | 54.3 (27.5) | 64.8 (23.8) | | 61.5 (26.7) | 58.9 (33.5) | | 51.5 (27.7) |
| % of sites with PPD ≥5 mm, mean (SD) | 42.2 (19.6) | 38.4 (18.1) | 50.4 (13.3) | | 47.1 (20.5) | 39.0 (20.8) | | 35.1 (16.2) |
| No. of sites with PPD ≤4 mm, mean (SD) | 83.3 (32.1) | 87.1 (30.3) | 61.7 (16.5) | | 73.8 (37.0) | 91.5 (32.9) | | 92.2 (26.3) |
| % of sites with PPD ≤4 mm, mean (SD) | 58.0 (19.4) | 60.8 (19.1) | 49.6 (13.3) | | 52.9 (20.5) | 61.1 (20.5) | | 63.8 (18.1) |
| CODS, mean (SD) | 4.0 (1.3) | 4.1 (1.0) | 4.6 (0.9) | | 4.8 (0.9) | 3.7 (1.3) | | 3.8 (0.9) |
| OHQoL-UK, mean (SD) | 42.7 (6.6) | 43.6 (8.7) | 41.5 (6.5) | | 44.1 (11.2) | 43.1 (6.7) | | 43.4 (7.7) |
