# Supplementary material for: A feasibility study with embedded pilot randomised controlled trial and process evaluation of electronic cigarettes for smoking cessation in patients with periodontitis
Source: Pilot Feasibility Stud. 2019 Jun 4;5:74. doi: 10.1186/s40814-019-0451-4 (PMC6547559; doi:10.1186/s40814-019-0451-4)
Supplement: Supplementary file 14 — Compliance with attending follow-up visits. Summary participant compliance with attending follow-up visits, including by recruitment source. (DOCX 13 kb) [file 40814_2019_451_MOESM14_ESM.docx]

Additional file 14. Compliance with attending visits

Summary of participant compliance with attending follow-up visits.

| **Compliance level** |  | **Randomisation group [n (%)]** | | |
| --- | --- | --- | --- | --- |
|  |  | **Control group**  **n=40** | **Intervention group**  **n=40** | **Total**  **n=80** |
| All reviews |  | 29 (73%) | 28 (70%) | 57 (71%) |
| 2/3 reviews |  | 2 (5%) | 3 (8%) | 5 (6%) |
| 1/3 reviews |  | 4 (10%) | 3 (8%) | 7 (9%) |
| 0/3 reviews |  | 5 (13%) | 6 (15%) | 11 (14%) |

**Participants attending 6-month visit by recruitment source.**

| **Recruitment source** | **No. of participants attending the 6-month visit** | **Percentage of participants attending the 6-month visit from those recruited from source** |
| --- | --- | --- |
| Periodontal new patient clinic | 20 | 83% |
| General restorative dentistry new patient clinic | 8 | 89% |
| Dental emergency clinic | 19 | 66% |
| Participant identification centre | 8 | 53% |
| Other | 3 | 100% |
| All | 58 | 73% |
