# Supplementary material for: A feasibility study with embedded pilot randomised controlled trial and process evaluation of electronic cigarettes for smoking cessation in patients with periodontitis
Source: Pilot Feasibility Stud. 2019 Jun 4;5:74. doi: 10.1186/s40814-019-0451-4 (PMC6547559; doi:10.1186/s40814-019-0451-4)
Supplement: Supplementary file 15 — Compliance with attending follow-up visits. Summary participant compliance with attending follow-up visits, including by recruitment source. (DOCX 27 kb) [file 40814_2019_451_MOESM15_ESM.docx]

**Additional file 15. Completion of weekly smoking questionnaire by study week**

3-month review

6-month review

Quit date

4-week review

Quit date and follow-up visits illustrated on figure. Note that week zero is the quit date (visit 2). Participants were provided with the weekly questionnaire at visit 1 and there was a variable duration between visits 1 and 2, although it was recommended this was not more than 4 weeks.
