# Supplementary material for: A feasibility study with embedded pilot randomised controlled trial and process evaluation of electronic cigarettes for smoking cessation in patients with periodontitis
Source: Pilot Feasibility Stud. 2019 Jun 4;5:74. doi: 10.1186/s40814-019-0451-4 (PMC6547559; doi:10.1186/s40814-019-0451-4)
Supplement: Supplementary file 16 — E-liquid flavour and strength selections. Detailed breakdown of the e-liquid flavour and strength selections. (DOCX 12 kb) [file 40814_2019_451_MOESM16_ESM.docx]

**Additional file 16. E-liquid flavour and strength selections**

**E-liquid flavour participant selection**

| **Flavour choice** | **Percentage of participants in intervention group (n=39) [% (n)]** |
| --- | --- |
| Tobacco only | 13% (5) |
| Cherry only | 10% (4) |
| Mint only | 21% (8) |
| Mint & Cherry | 15% (6) |
| Tobacco & Cherry | 5% (2) |
| Tobacco & Mint | 23% (9) |
| Flavourless only | 0 |
| Flavourless & Tobacco | 8% (3) |
| Flavourless & Mint | 3% (1) |
| Flavourless & Cherry | 3% (1) |

**E-liquid strength (nicotine) participant selection**

| **Nicotine choices (mg/ml)** | | **Percentage of participants in intervention group (n=39) [% (n)]** |
| --- | --- | --- |
| Choice 1 | Choice 2 |  |
| 0 | 0 | 0 |
| 0 | 6 | 0 |
| 0 | 12 | 0 |
| 0 | 18 | 0 |
| 6 | 6 | 0 |
| 6 | 12 | 5% (2) |
| 6 | 18 | 0 |
| 12 | 12 | 18% (7) |
| 12 | 18 | 54% (21) |
| 18 | 18 | 23% (9) |
