# Supplementary material for: A feasibility study with embedded pilot randomised controlled trial and process evaluation of electronic cigarettes for smoking cessation in patients with periodontitis
Source: Pilot Feasibility Stud. 2019 Jun 4;5:74. doi: 10.1186/s40814-019-0451-4 (PMC6547559; doi:10.1186/s40814-019-0451-4)
Supplement: Supplementary file 17 — Use of non-recommended e-liquid or device. Details of those participants who used non-recommended e-liquid or devices during the study. (DOCX 17 kb) [file 40814_2019_451_MOESM17_ESM.docx]

Additional file 17. Use of non-recommended e-liquid or device

Use of non-recommended e-liquids (intervention group)

| **Participant** | **E-liquid brand** | | | |  | **Smoking quit status** | |
| --- | --- | --- | --- | --- | --- | --- | --- |
|  | **Quit date** | **4 weeks** | **3 months** | **6 months** |  | **RS6-eCO** | **RS6-S** |
| 1001 | Recommended | Unknown | Unknown | # |  | Smoker | Smoker |
| 1003 | Recommended | TECC | Totally wicked | Totally wicked |  | Smoker | Smoker |
| 1013 | Recommended | Recommended | Recommended | Unknown |  | Quitter | Quitter |
| 1018 | Recommended | Recommended | Cirro | Cirro |  | Quitter | Quitter |
| 1030 | VIP | * | * | * |  | Smoker | Smoker |
| 1035 | Recommended | Recommended | Unknown | Vaporized |  | Smoker | Smoker |
| 1051 | Recommended | Vaporized | Vaporized | Recommended |  | Quitter | Smoker |
| 1054 | Unknown | Vape 888 | Vape 888 | Vape 888 |  | Quitter | Smoker |
| 1061 | Nicocig | Nicocig | Nicocig | # |  | Smoker | Smoker |
| 1068 | Recommended | Recommended | Unknown | * |  | Smoker | Smoker |
| 1075 | Edge | Edge | Edge | Recommended |  | Quitter | Quitter |
| Total using non-recommended brands (n) | 4 | 6 | 9 | 5 |  |  |  |

Data only presented for those participants in the intervention group who reported use of non-recommended e-liquids on at least one time point. *Did not attend visit. #Stopped using a e-cigarette.

**Use of different e-cigarette device (intervention group).**

| **Participant** | **E-liquid brand** | | | |  | **Smoking quit status** | |
| --- | --- | --- | --- | --- | --- | --- | --- |
|  | **Quit date** | **4 weeks** | **3 months** | **6 months** |  | **RS6-eCO** | **RS6-S** |
| 1003 | Recommended | TECC arc mini | TECC arc mini | TECC arc mini |  | Smoker | Smoker |
| 1035 | Recommended | Recommended | Unknown 3^rd^ generation | Unknown 3^rd^ generation |  | Smoker | Smoker |
| 1051 | Recommended | Recommended | PRISM T18E | PRISM T18E |  | Quitter | Smoker |
| 1054 | Recommended | Vype- other** | Vype-other** | Vype-other** |  | Quitter | Smoker |
| 1061 | Nicocig | Nicocig | Nicocig | # |  | Smoker | Smoker |
| 1068 | SMOCK | SMOCK | SMOCK | * |  | Smoker | Smoker |
| Total using different device (n) | 2 | 4 | 6 | 4 |  |  |  |

*Did not attend visit. #Stopped using a e-cigarette. **Participant lost their e-cigarette and tried to purchase an exact replacement of that provided in the study. The model used was no longer available and the participant purchase a newer model.
