# Supplementary material for: A feasibility study with embedded pilot randomised controlled trial and process evaluation of electronic cigarettes for smoking cessation in patients with periodontitis
Source: Pilot Feasibility Stud. 2019 Jun 4;5:74. doi: 10.1186/s40814-019-0451-4 (PMC6547559; doi:10.1186/s40814-019-0451-4)
Supplement: Supplementary file 18 — Compliance with e-cigarette usage (intervention group). Compliance of the intervention group with e-cigarette usage. (DOCX 12 kb) [file 40814_2019_451_MOESM18_ESM.docx]

Additional file 18. Compliance with e-cigarette usage (intervention group)

|  | **Participants using e-cigarette**  **[n (%, 95% CI)]** | **Participants not using e-cigarette**  **[n (%, 95% CI)]** | **Participants who did not attend the visit**  **[n (%, 95% CI)]** |
| --- | --- | --- | --- |
| Visit 2 (Quit date) | 36 (90%, 95% CI: 77%-96%) | 2 (5%, 95% CI: 1%-17%) | 2 (5%, 95% CI: 1%-17%) |
| Visit 4 (4 weeks) | 31 (78%, 95% CI: 63%-88%) | 1 (3%, 95% CI: 0%-13%) | 8 (20%, 95% CI: 11%-35%) |
| Visit 5 (3 months) | 28 (70%, 95% CI: 55%-82%) | 5 (13%, 95% CI: 5%-25%) | 7 (18%, 95% CI: 9%-32%) |
| Visit 6 (6 months) | 21 (53%, 95% CI: 38%-67%) | 8 (20%, 95% CI: 11%-35%) | 11 (28%, 95% CI: 16%-43%) |
