# Supplementary material for: A feasibility study with embedded pilot randomised controlled trial and process evaluation of electronic cigarettes for smoking cessation in patients with periodontitis
Source: Pilot Feasibility Stud. 2019 Jun 4;5:74. doi: 10.1186/s40814-019-0451-4 (PMC6547559; doi:10.1186/s40814-019-0451-4)
Supplement: Supplementary file 19 — Qualitative process evaluation. Further details of the qualitative process evaluation with illustrative quotes. (DOCX 28 kb) [file 40814_2019_451_MOESM19_ESM.docx]

### Additional file 19. Qualitative process evaluation

### Demographic data

Twenty-eight participants completed baseline interviews shortly after intervention delivery (6 to 48 days after intervention [mean: 25 days]). General views on e-cigarettes were explored with all twenty-eight participants. A more detailed exploration of acceptability issues was conducted for the fourteen participants who had been provided with an e-cigarette starter kit. These fourteen participants were aged 27-60 years (mean age: 44 years). Participants were heavy smokers, all smoking at least 10 cigarettes per day (median number of cigarettes per day: 15; mean eCO: 21ppm) with a moderate level of nicotine dependence (mean FTND: 5). In terms of employment, an almost equal numbers of participants were in professional, intermediate and routine/manual/unemployed categories. There were nine females and five males.

### Overview of themes

Smokers with periodontitis had a range of experiences and perceptions regarding e-cigarettes. Five over-arching themes emerged from the data: influence of other e-cigarette users; previous e-cigarette experience; concerns about addiction to e-cigarette/nicotine; health considerations; and social acceptability of e-cigarettes. When considering those who had been offered the e-cigarette starter kit, three additional themes emerged, comprising the benefit of behavioural similarities with smoking (hand-to-mouth, vapour, habit); the influence of e-cigarette flavours; and technical issues.

Direct quotes are provided and individual participant characteristics (gender, age, average number of cigarettes per day) are shown in brackets following each quote.

### Influence of other e-cigarette users

Many of the participants reported knowing existing users of e-cigarettes. These e-cigarette users almost always acted as a positive influence towards vaping through their conversations or actions.

…a lot of people I know, they seem, quite positive about it [vaping], and I haven’t had any sort of negative, feedback or anything from it so. I think they kind of like think, “Well it’s, probably a lot better than a lot of the, junk and stuff that’s actually in a normal cigarette. (Male, 45 years, 15 cigs/day)

In this case, the participant knew many e-cigarette users and he perceived them to be positive about vaping. He compared vaping to conventional smoking and felt that the e-cigarette users he knew thought it would be ‘a lot better’. Other participants reported similarly positive impressions by observations of people around them.

A lot of the girls at work, have been really successful on the e-cigarettes… about six or seven of the girls at work have stopped smoking with the e-cigarettes... and they’re supportive as well, the girls at work are dead supportive as well. (Female, 52 years, 15 cigs/day)

This participant had experienced many of her workplace friends successfully quitting smoking by using the e-cigarette. At the time of the interview this participant had quit smoking using an e-cigarette (provided in the study). The support of her vaping friends appeared to be an important influence on her continued use of the e-cigarette. Other participants were aware of the positive views of e-cigarette users but still sceptical themselves.

a lot of people say, “Well it’s a lot better than smoking”… the amount of people using them now it’s ridiculous, when you actually look around in like a smoking area outside where I work, it is quite crazy how, it’s kind of weird, so a lot of people must have a positive views of them as well you know. (Male, 27 years, 15 cigs/day)

This participant had noted the large number of e-cigarette users in his environment and presumed they had positive views on e-cigarettes. However, his choice of language (e.g. ridiculous and weird) suggests he is unconvinced himself and uncomfortable with the number of e-cigarette users.

### Previous e-cigarette experience

The eligibility criteria for the current study required participants to have not used an e-cigarette regularly, within the 30 days prior to enrolment. However, many had previous personal experience of e-cigarettes from an earlier time period. Over two-thirds (68%) reported prior use of an e-cigarette, of whom around a third (37%) had previously used an e-cigarette on a daily basis. This previous experience varied considerably from having used multiple devices in successful quit attempts (prior to relapse) to having a few puffs on friend’s devices. Overall, however, these participants’ previous experience of e-cigarettes was predominantly negative.

I did [previously use an e-cigarette], but it was when they first came out and it used to just leak all over the place in me [my] pocket, and the one that I’ve got off you is just brilliant because it doesn’t leak. And that’s why I gave up on, that [previously].” (Female, 52 years, 15 cigs/day)

As in this case, the negative previous experience was sometimes due to technical or practical issues. This participant found the previous device leaked and that caused her to stop using it. In other cases, the negative experiences were related to the sensations experienced by the participant.

Well I’ve only tried one, nearly took the back of me [my] throat out, so I just, [laughs]… didn’t bother. Cos I think I just had it on the wrong setting… Yeah. I couldn’t stop [coughing], I thought I was going to choke… It was horrible. (Female, 57 years, 20 cigs/day)

… it tasted really funny, you could taste like the, it sounds weird but you could actually taste like electricity in your mouth which was very weird. (Female, 45 years, 10 cigs/day)

These quotes illustrate typical examples of previous negative e-cigarette experiences described by participants. In the first quote the participant experienced an unpleasant sensation in her throat followed by a cough which she described as ‘horrible’. The second quote is more related to an unpleasant taste or sensation in the participant’s mouth. In other cases, participants went to the effort of sourcing and purchasing e-cigarettes but were put off using them for other reasons other than a negative experience of actually trying it.

I’ve bought two different types [of e-cigarette], I bought the ones with the batteries and I’ve bought the liquid ones… But I’ve been frightened to use them… I just didn’t have the confidence to use them, because you know when you see them on the television... and then there’s sort of like, the scare, things... (Female, 58 years, 15 cigs/day)

Concerns about the safety and health harms of e-cigarettes, as reported in the media, are cited by this participant to be reasons for her not using the e-cigarette she purchased.

### Concerns about addiction to e-cigarette/nicotine

Many participants reported concerns about addiction to either e-cigarettes or nicotine. Some expressed this as worries about becoming over-reliant on the e-cigarette with several participants describing a negative image of a ‘vaper’ with an e-cigarette constantly in their mouth.

…me [my] brother in law, he’s really addicted to his... the vapour pen, and me [my] wife says, she doesn’t want me addicted to that… He packed in [smoking] before and just had nothing, but he’s packed in this time, and his vapour pen’s never out of his mouth… I don’t want to be like, walking like around and like having just a vapour pen in my mouth all the time you know what I mean… I’ll wean myself off this... (Male, 40 years, 30 cigs/day)

In this quote, the participant describes a family member who he feels had become over-reliant on an e-cigarette. The participant has anxieties about his own use, based upon these concerns, and planned to reduce his e-cigarette use. Some of the participants disliked the idea of still being ‘addicted’ to something following switching (from tobacco cigarettes to e-cigarettes).

I know people who have tried e-cigarettes... and they’re ridiculous, they just smoke them [the] same as what they did, and they become addicted to them. So it’s like, to me, it feels like they’re just replacing one addiction for another... (Female, 49 years, 15 cigs/day)

This quote illustrates concerns about simply replacing one perceived addiction with another (e-cigarettes replacing tobacco cigarettes). Worries about a lack of control with e-cigarettes compared to tobacco cigarettes were also voiced.

...when I’m smoking [tobacco] cigarettes I know exactly what I’m getting. If I move to a vaper [e-cigarette], I don’t really know how much, how much of a hit I’m getting… [If I used tobacco cigarettes] I think it would be easier for me to do it [cut down], to measure what I’m having you know. And less, and less, and less. (Male, 44 years, 30 cigs/day)

Participants were comfortable with their tobacco smoking routine, often having done this for multiple decades. They voiced worries about the lack of control when moving to e-cigarettes, finding ‘dosing’ difficult to measure. In this case, the participant used this as a reason for continuing to use tobacco cigarettes as they felt they had more control for cutting down.

### Health considerations

The possible health implications of using e-cigarettes were regularly discussed by participants who expressed a range of opinions. Some participants believed the e-cigarettes to be less harmful to their health than tobacco cigarettes.

…it’s [vaping’s] like as if I’m having a cigarette but it’s not doing as much harm. (Male, 40 years, 30 cigs/day)

I feel better for it [switching to e-cigarettes]. I really do. I don’t feel like I smell… me [my] mouth’s a lot healthier, I’m not wheezing, I’m not coughing, on a morning, it’s, I just feel in myself, a lot healthier for packing in real cigarettes and going on to the e-cigarette. It’s a massive difference. (Female, 52 years, 15 cigs/day)

These quotes illustrate that some participants held the opinion that e-cigarettes were less harmful than tobacco cigarettes. In the first quote, this appears to be an opinion based on general knowledge whereas in the second quote, the view is based on, or reinforced by, personal experience of improved health after switching to e-cigarettes from tobacco cigarettes. Other participants, and their relatives, had negative views of the health harms from e-cigarettes, often citing media stories.

I did go on the e-cigarettes for a while… But then me Mam [mum], [fell] seriously ill, and I’ve actually been looking up this week about it and I think... from what they described, apart from the tumour she had Popcorn lung…Which is part of the e-cigarette… or so they think. (Female, 47 years, 15 cigs/day)

In this example, the participant had a family bereavement of a relative who was a life-long smoker and recent e-cigarette user. The participant has conducted her own research and cites popcorn lung as a health harm from e-cigarettes. Another participant also specifically mentioned popcorn lung in this context.

### Social acceptability

The participants expressed a range of perceptions about the social acceptability of e-cigarettes. Some of the participants felt they were socially acceptable with lots of other people using e-cigarettes around them.

I think at the moment they are [socially acceptable]. I think they’ve almost become a bit of a fashion trend. A bit trendy at the moment. (Male, 56 years, 20 cigs/day)

Although this participant feels e-cigarettes are socially acceptable, they suggest this might be transient and just a short lived trend. Many participants were comfortable using the e-cigarette around family, friends and in public.

…I don’t feel out of place when I’m using it, I don’t feel uncomfortable using it, walking through town or wherever I feel I need it you know. (Male, 27 years, 15 cigs/day)

Others went further and felt the e-cigarette was accepted by smoking culture.

Yeah, very acceptable because a lot of people… go outside to smoke a cigarette but a lot of people go outside to smoke their e-cig as well… So it’s just a case of just general people just standing outside, and I think because you see the smoke, you don’t actually think of it as an e-cig, you just think, yeah, you’re just fitting in. (Female, 45 years, 10 cigs/day)

This quote illustrates how a smoker has switched to e-cigarettes and feels their social interactions are unchanged. Many participants describe choosing to continue going outside when vaping as if they were still smoking.

...I’ve just slotted it [e-cigarette] in exactly what I would normally do. So, even if it is raining, and sometimes I think, “What are you doing stood outside having a cigarette on a morning, when it’s raining?” Like, “How stupid are you?” I’ve still done it, this morning, I just thought, “No, just go out, and just do it, doesn’t matter if you’re going to get wet”, because really, I could have it [e-cigarettes] in the house, but I don’t want to start that. (Female, 35 years, 10 cigs/day)

In this case, the participant is choosing to restrict her use of the e-cigarette to outside areas, even in her home environment. She talks about not wanting to get into a habit of using the e-cigarette inside her house. Other participants chose to use their e-cigarette outside to continue social interactions.

…when I go out socialising, me [my] friends go out for a cigarette, a lot of places you can’t do the e-cig inside, so I’ve been going out with them but just using that, and it’s psychologically [psychological], I’m still smoking with them but obviously not smoking [tobacco] cigarettes. (Female, 45 years, 10 cigs/day*)*

It was important to a number of participants to be able to continue their social routine when switching to e-cigarettes and hence they often chose to self-enforce smoking restrictions on their e-cigarette use. Others describe regulations forcing them to go outside in some environments.

[At work] Yeah well I still have to go outside with that [e-cigarette]. (Female, 60 years, 15 cigs/day)

Several participants perceived negative perceptions towards e-cigarettes and vapers. Some experienced negative comments personally, while others perceived negative attitudes more widely.

I suppose it’s as frowned upon as a cigarette because, people aren’t told how... what’s in an e-cigarette. And what’s coming out of an e-cigarette. It’s certainly, I’m sure, 90% or 95% of people, if you stood next to them with an e-cigarette [they] would shy away as they would with a normal cigarette... When I’m out socially I wouldn’t use it. (Male, 58 years, 15 cigs/day)

In this case, the participant describes the negative perceptions towards e-cigarettes they perceived by the general public and how it means they wouldn’t use an e-cigarette when out socially. Other participants were uncomfortable about how vaping looked.

I think if you see someone with a, e-cig, you just know that they used to be a smoker, it just doesn’t look attractive. I’ll be honest but I don’t think either [e-cigarettes or tobacco cigarettes], look attractive… I think they’re awful. (Female, 35 years, 10 cigs/day)

…some of the girls at work, ee, honest to God they’ve got these, chambers, they’re like... I’m like, “Oh my God, do you really need that? (Female, 52 years, 15 cigs/day)

In the first quote, the participant feels vaping, due to it’s similarly to tobacco smoking, is unattractive. The second quote focuses on the more complex devices of which a number of participants had negative opinions. One participant was particularly insightful about the changing social norms as the prevalence of e-cigarettes in the population increases. They felt embarrassment using e-cigarettes in previous quit attempts but this was reduced in the current quit attempt due to more widespread use.

…when I stopped the last time, for the three months, with it [e-cigarette], there wasn’t as many people using them... and I did feel, quite embarrassed, going out and using them… But now, there’s such an influx of people actually using them [e-cigarettes] it is quite strange, if you’re walking round with a cigarette now rather than the e-cigarette. (Female, 47 years, 15 cigs/day)

### Benefit of behavioural similarities

Many of the participants reflected that the e-cigarette provided a more fulfilling experience than simply supplying nicotine. Its similarity to tobacco smoking was perceived to be highly beneficial in terms of helping participants to quit.

It has helped, because, I don’t know whether you can appreciate this. It’s like having a pen in your hand. You need something to do with your hands. (Male, 58 years, 15 cigs/day)

The most commonly mentioned feature was the tactile sensation of holding something in their hands or the ‘hand-to-mouth’ action, as illustrated in the above quote. Other sensations such as vapour production or the ‘throat hit’ were important for several of the participants.

…and that’s where I find it good with the e-cigarette because it replaces that. And you can see the smoke blowing out, you get the throat hit, and it’s, it’s exactly, a replacement and it’s, aye, I’ve really, found it [quitting tobacco cigarettes] quite, easy, in a way. (Female, 52 years, 15 cigs/day)

The closeness of the actions and sensations of vaping compared to tobacco smoking were important for this participant and reportedly led to an easy quit attempt. The quote below illustrates the strength of feeling and deep consideration some participants had on this topic.

…you know the e-cigarettes, the good ones you can get now, I find I can get a good inhale and blow a lot of smoke out and that’s the type of sensation I like the most. I mean, if I sucked something in, and didn’t blow anything out, it doesn’t seem as though I had a cigarette. You see?... But although when I get the actual, feel this vapour or whatever it is, it’s that which is probably the psychological thing. You see because, just supposing they made a wonder tablet, and I took that tablet, mmm, made us feel like I didn’t feel like a smoker, in my head and that would still say, “I want a [breath in/out] this bit”, although this wonder tablet stopped that craving for the nicotine shall we say. I believe so, really do. Especially with being smoking for 40 years. It’s that sensation. (Male, 59 years, 30 cigs/day)

The behavioural similarities to tobacco smoking were vitally important attributes of the e-cigarette for many of our participants, especially those who have been tobacco smoking for most of their life, as in the above quote.

### Perceptions of the e-cigarette starter kit

Of the forty participants who were offered an e-cigarette starter kit as part of the feasibility RCT, thirty-nine accepted it (14 of these completed interviews). One participant declined the kit on the grounds that they did not intend to change their smoking behaviour. Generally, participants reported that they found the e-cigarette starter kit acceptable.

I thought that [the e-cigarette starter kit] was amazing. You know the quality of the e-cigarette that you’ve given me and the liquids and you’ve given me the, you know, the tutorial about how to use the e-cigarette. (Female, 47 years, 10 cigs/day)

Furthermore, participants felt the quantity of supplies were sufficient.

And it does give you a few weeks to get into the routine of it… And I really don’t see why, the NHS, whatever [or whoever], should have to pay after that… And when you think about it, the amount of money that that is, considering a packet of cigarettes, and how long it lasts, ‘cos they [e-cigarettes] do last a long time... (Female, 47 years, 15 cigs/day)

The starter kit was designed to last for 2-3 weeks to give the participants a reasonable trial period and allow them to source their own supplies. The participant in this quote reflects that it lasted long enough for them to get into the routine of using it. She also felt that morally the NHS (or other healthcare provider) shouldn’t need to supply any more than this and that the costs of supplies were relatively minimal. Participants reported finding it straightforward to source their own supplies either at pharmacists, corner shops or online.

I think, using it for a week you’re going to decide whether it’s going to be the right tool for you to stop smoking or not, so, I think two bottles is fair… Yeah, they’re all about aren’t they [e-liquids]. You’re right, they sell ‘em in Boots [pharmacists], they sell ‘em in McColl’s [newsagents], they sell ‘em, everywhere you want to go... (Male, 27 years, 15 cigs/day)

Participants were provided with two batteries and a number reflected on the usefulness of this.

It’s [using the e-cigarette] all about organisation. It’s like I’ve got one battery on charge and I’m using the other one. You know, so when I’ve finished that one, that one can go straight on. So you haven’t got that time that you haven’t got an E-Cigarette, that would be the time that you would pick up a [tobacco] cigarette. (Female, 52 years, 15 cigs/day)

This participant also felt the provision of two batteries was essential in preventing relapse to tobacco smoking by ensuring that the e-cigarette was always charged when she needed to use it.

### Influence of flavour

Participants were provided with a choice of four e-liquid flavours: tobacco, mint, cherry and unflavoured. Participants had relatively dichotomous views on flavour preference. The first group held strong preferences against tobacco flavours, believing this might be too similar to tobacco smoking.

I don’t know, ‘cos I feel like if you’re going to quit [tobacco smoking] you might as well not have cigarette flavour because it’s defeating the point, to me anyway. So and I love cherry flavoured stuff so, cherry and minty [is what I used]. (Female, 31 years, 30 cigs/day)

This quote illustrates a participant who preferred the non-tobacco flavours and felt using a tobacco flavored e-cigarette would undermine the quit attempt. As in this case, several participants had a preference for mint flavours.

I think just with the mint… it’s a fresh, it’s nice and fresh, on your breath, and that. It’s just, it’s a pleasant taste. (Female, 52 years, 15 cigs/day)

Mint or menthol flavours were seen as refreshing and spoken about positively by a number of participants. The second group held the view that the e-liquid flavour should be similar to their smoking experience so chose the tobacco flavour.

I think I chose the tobacco [flavour e-liquid] because I was a little bit frightened that, it would have been too much of a leap from going from no cigarettes to pure mint… But to be honest, the thought now, of going back to tobacco [e-liquid], after using the mint [e-liquid], I didn’t want to go back to the tobacco [e-liquid], I didn’t want to use the tobacco one, I just want the mint one ‘cos I feel as though it’s like, fresh… And I didn’t, I don’t want that stale taste in me [my] mouth again. (Female, 52 years, 15 cigs/day)

Trying to maintain the similarity to tobacco smoking by using tobacco flavoured e-liquid was seen as important by this participant. They then transitioned to mint flavour after using the e-cigarette for a while and subsequently felt that they would be unlikely to go back to the tobacco flavour.

I went for the normal mixture like the tobacco one and the menthol one as well… I quite like the idea of mirroring the flavours. I think some of these, you know, oh, sweet bubble gum, is ridiculous, because if I want bubble gum, I go and buy bubble gum… I think it should be a, similar flavour, shall we say. (Male, 56 years, 20 cigs/day)

Again this quote illustrates the importance some participants placed on ‘mirroring’ the flavours they were familiar with when tobacco smoking, which also included menthol cigarettes in this case. This participant spoke about the tobacco and menthol flavours as ‘normal’ and reflected that the wider range of fruity/sweet flavours were ‘ridiculous’ which was a common theme in several interviews. However, this was not universal, with some participants keen to try all flavours. Conversely, one participant was keen to avoid any flavour and chose the unflavoured e-liquid.

…‘cos I want to get away from... the tobacco taste. But with the fruit one, like I say, me [my] chest [harsh on chest]. But then I wanted something sweet afterwards… So that’s defeated the object for me. ‘Cos I don’t want to put loads more weight on, so I think, you know, if I don’t want anything sweet... (Female, 40 years, 25 cigs/day)

This participant cited several reasons for her choice of the unflavoured e-liquid. She wanted to avoid the tobacco taste, to break away from smoking. She had a previous unpleasant experience of the fruit flavours, which she had perceived to be harsh on her chest and made her want to eat something sweet after use, causing concerns about weight gain.

### Technical issues

A small number of our participants reported technical issues with the e-cigarettes provided in the study.

I took my e-cigarette to work on Saturday... and for some reason the damn thing’ll not work… I don’t know if it’s broken... because, when you inhale it… You’re supposed to press the button, wait until it, thingies… But then you’re getting, your lips is getting covered in oil. (Female, 60 years, 15 cigs/day)

This participant experienced problems with one of the tanks provided as part of the starter kit, having e-liquid leak onto her lips. This was an isolated incident but did highlight that operating the device was not always straightforward for all participants, including those who had never used an e-cigarette prior to agreeing to join the study.

Assistance with operating devices was sometimes obtained from family or friends if technical issues arose.

My son’s 22 and he was, he’s been very helpful, as in, you know, showing me what to do with the e-cigarette and making sure it was charged and that I’ve got enough liquid, and how to put the liquid in, just reminding me again how to do it… I’d arranged everything around… [going] to the caravan, to enable me to quit. I had got up that morning, thought “Right, OK, this is the day I’m going to do it”, and then tried to use the E-Cigarette, [but it] didn’t work… and I hadn’t took [taken] the battery charger with me… Or the charging USB. So then, I rang my son, he said. “Don’t worry, when you come back, just do it when you come back”. Anyway, when I went back, he tried it and said you haven’t clicked it five times before it sort of starts and I was like, “Oh, right, OK”, and he said, “Well, start tomorrow”. Which I did do, yeah. (Female, 47 years, 10 cigs/day)

The quote highlights the importance of the participant’s son in helping her overcome initial issues with using the e-cigarette. Despite being provided with instructions on operating the device by the researcher, a number of participants struggled with the automatic safety lock which requires the button to be pressed 3 times in close succession to deactivate.
