# Supplementary material for: A feasibility study with embedded pilot randomised controlled trial and process evaluation of electronic cigarettes for smoking cessation in patients with periodontitis
Source: Pilot Feasibility Stud. 2019 Jun 4;5:74. doi: 10.1186/s40814-019-0451-4 (PMC6547559; doi:10.1186/s40814-019-0451-4)
Supplement: Supplementary file 20 — E-cigarette use in the control group. Details of those participants in the control group who used e-cigarettes. (DOCX 12 kb) [file 40814_2019_451_MOESM20_ESM.docx]

**Additional file 20. E-cigarette use in the control group**

| **Participant** | **Using an e-cigarette?** | | | |  | **Smoking quit status** | |
| --- | --- | --- | --- | --- | --- | --- | --- |
|  | **Quit date** | **4 weeks** | **3 months** | **6 months** |  | **RS6-eCO** | **RS6-S** |
| 1006 | Yes | Yes | No | No |  | Quitter | Quitter |
| 1008 | Yes | Yes | No | No |  | Smoker | Smoker |
| 1041 | No | No | Yes | No |  | Smoker | Smoker |
| 1063 | Yes | Yes | Yes | Yes |  | Quitter | Smoker |
| 1065 | No | No | No | Yes |  | Smoker | Smoker |
| 1067 | Yes | Yes | Yes | No |  | Smoker | Smoker |
| 1071 | No | No | Yes | Yes |  | Smoker | Smoker |
| 1078 | No | Yes | Yes | No |  | Smoker | Smoker |
| Total (n) | 4 | 5 | 5 | 3 |  |  |  |
