# Supplementary material for: A feasibility study with embedded pilot randomised controlled trial and process evaluation of electronic cigarettes for smoking cessation in patients with periodontitis
Source: Pilot Feasibility Stud. 2019 Jun 4;5:74. doi: 10.1186/s40814-019-0451-4 (PMC6547559; doi:10.1186/s40814-019-0451-4)
Supplement: Supplementary file 21 — Methods of smoking cessation used by randomisation group. Description of the methods of smoking cessation methods used by the participants. (DOCX 12 kb) [file 40814_2019_451_MOESM21_ESM.docx]

Additional file 21. Methods of smoking cessation used by randomisation group.

| **Smoking cessation method** | **Randomisation group [n (%)]** | | |
| --- | --- | --- | --- |
|  | **Control group**  **n=40** | **Intervention group**  **n=40** | **Total**  **n=80** |
| NHS stop smoking services | 16 (40%) | 0 | 16 (20%) |
| General Medical Practitioner (GMP) support | 2 (5%) | 0 | 2 (3%) |
| NRT (non-e-cigarette) | 8 (20%) | 4 (10%) | 12 (15%) |
| Varenicline (Champix) | 5 (13%) | 0 | 5 (6%) |

Some participants used multiple methods e.g. NHS stop smoking service and NRT.
