# Supplementary material for: A feasibility study with embedded pilot randomised controlled trial and process evaluation of electronic cigarettes for smoking cessation in patients with periodontitis
Source: Pilot Feasibility Stud. 2019 Jun 4;5:74. doi: 10.1186/s40814-019-0451-4 (PMC6547559; doi:10.1186/s40814-019-0451-4)
Supplement: Supplementary file 22 — Data completeness. Details of data completeness for the outcome measures. (DOCX 15 kb) [file 40814_2019_451_MOESM22_ESM.docx]

**Additional file 22. Data completeness**

| **Data element** | **Visit** | **Consented for procedure** | **Completed procedure/ data collected** | **Data completeness** | **Comment** |
| --- | --- | --- | --- | --- | --- |
| Pocket Probing Depths | Baseline | 80 | 80 | 100% | 1030- Data not collected from three teeth. The indices were being taken alongside the patient’s treatment under local anaesthetic due to anxiety. They failed to attend part way through these sessions hence the data incompleteness. |
|  | Visit 5 | 64 | 61 | 95% | 1040, 1048, 1058- Oral health indices not collected on three participants at visit 5. This is because their periodontal treatment had extended beyond the original 2 visits or had been delayed, meaning not enough healing time has elapsed following the treatment to collect the indices. Smoking outcome data was still collected. |
|  | Visit 6 | 58 | 58 | 100% |  |
| Gingival Index | Baseline | 80 | 80 | 100% | See comments in PPD comments above |
|  | Visit 5 | 64 | 61 | 95% | See comments in PPD comments above |
|  | Visit 6 | 58 | 58 | 100% |  |
| Plaque Index | Baseline | 80 | 80 | 100% | See comments in PPD comments above |
|  | Visit 5 | 64 | 61 | 95% | See comments in PPD comments above |
|  | Visit 6 | 58 | 58 | 100% |  |
| Bleeding on Probing | Baseline | 80 | 80 | 100% | See comments in PPD comments above |
|  | Visit 5 | 64 | 61 | 95% | See comments in PPD comments above |
|  | Visit 6 | 58 | 58 | 100% |  |
| Clinical Attachment Loss | Baseline | 80 | 80 | 100% | See comments in PPD comments above |
|  | Visit 5 | 64 | 61 | 95% | See comments in PPD comments above |
|  | Visit 6 | 58 | 58 | 100% |  |
| Clinical Oral Dryness Score | Baseline | 80 | 80 | 100% |  |
|  | Visit 5 | 64 | 61 | 95% | See comments in PPD comments above |
|  | Visit 6 | 58 | 58 | 100% |  |
| Expired air Carbon Monoxide | Baseline | 80 | 80 | 100% |  |
|  | Visit 2 | 76 | 75 | 99% | Carbon monoxide machine failure (1046) |
|  | Visit 4 | 67 | 66 | 99% | Carbon monoxide machine failure (1006) |
|  | Visit 5 | 64 | 64 | 100% |  |
|  | Visit 6 | 58 | 58 | 100% |  |
| Saliva sample | Baseline | 80 | 80 | 100% |  |
|  | Visit 2 | 76 | 76 | 100% |  |
|  | Visit 4 | 67 | 67 | 100% |  |
|  | Visit 5 | 64 | 64 | 100% |  |
|  | Visit 6 | 58 | 58 | 100% |  |
| Subgingival plaque sample | Baseline | 80 | 80 | 100% |  |
|  | Visit 2 | 76 | 76 | 100% |  |
|  | Visit 5 | 64 | 61 | 95% | See comments in PPD comments above |
|  | Visit 6 | 58 | 58 | 100% |  |
| Gingival crevicular fluid sample | Baseline | 80 | 80 | 100% |  |
|  | Visit 5 | 64 | 61 | 95% | See comments in PPD comments above |
|  | Visit 6 | 58 | 58 | 100% |  |
| Fagerstrom Test for Nicotine Dependence  (6 items) | Baseline | 80 | 80 | 100% |  |
|  | Visit 2 | 76 | 76 | 100% |  |
|  | Visit 4 | 67 | 67 | 100% |  |
|  | Visit 5 | 64 | 64 | 100% |  |
|  | Visit 6 | 58 | 58 | 100% |  |
| Mood and Physical Symptoms Scale  (12 items in three subsections with 7,2,3 questions) | Baseline | 80 | 80 | 100% | Participant 1021 missed 5 items from this questionnaire so has been classed as missing in data analysis. |
|  | Visit 2 | 76 | 76 | 100% |  |
|  | Visit 4 | 67 | 67 | 100% |  |
|  | Visit 5 | 64 | 64 | 100% |  |
|  | Visit 6 | 58 | 57 | 99% | Participant 1071 missed two items from this questionnaire. Following the ‘rule of halves’ it was not possible in impute means, as more than half of the subsection was missing, and the whole questionnaire was classed as missing. |
| Oral Health Quality of Life Questionnaire  (16 items) | Baseline | 80 | 80 | 100% |  |
|  | Visit 6 | 58 | 58 | 100% |  |
