# Supplementary material for: A feasibility study with embedded pilot randomised controlled trial and process evaluation of electronic cigarettes for smoking cessation in patients with periodontitis
Source: Pilot Feasibility Stud. 2019 Jun 4;5:74. doi: 10.1186/s40814-019-0451-4 (PMC6547559; doi:10.1186/s40814-019-0451-4)
Supplement: Supplementary file 23 — Sample size calculation and pooled standard deviations of outcome measures. Sample size calculation and pooled standard deviations of periodontal outcome measures. References: [33, 36–38, 40, 47–56]. (DOCX 30 kb) [file 40814_2019_451_MOESM23_ESM.docx]

**Additional file 23. Sample size calculation and pooled standard deviations of periodontal outcome measures**

For the smoking abstinence outcome measure, we propose to use Russell Standard 6-month sustained abstinence, defined as self-report of smoking no more than five cigarettes in the previous 6 months and not smoking in the previous week, verified by an eCO reading below 10 ppm. A control group rate of smoking abstinence at 6 months of 7% was used based on previous research [1] and in keeping with the rate seen in this pilot study (5%, 95% CI: 1%-17%). We chose a minimally clinically important difference (MCID) of 8% in keeping with previous studies which have used MCID in the range 6% to 10% [2-8]. Therefore, in order to detect an 8% difference in 6-month smoking abstinence rates between intervention arms, with a control group rate of 7% (90% power, 5% significance level, two-sided test) 337 participants will be required per arm, 674 in total [9]. Given any randomised participants who are lost to follow-up will be included in the ITT analysis of smoking abstinence as smokers at 6 months, this sample size has not been adjusted for attrition. However, the consent rate amongst those eligible in the pilot trial was 67%, (95% CI: 58%-75%). Given that the definitive study will be conducted in multiple centres and the pilot trial was conducted in a single centre (and as part of a doctoral fellowship), it would be prudent to use the lower bound of this 95% CI and on this basis, the future definitive study would need to approach 1162 potentially eligible patients in order to consent and randomise 674.

For the periodontal health outcome measure there are several potential outcome measures that could be used. Whole mouth mean PPD is often used, despite not being the best method to assess clinical significance [10]. This calculation uses a MCID of 0.25 mm in line with previous research [11]. Pooled standard deviations of the change from baseline to 6 months for the outcome measures assessed in the pilot trial are presented below. For PPD the pooled standard deviation was 0.56mm. Therefore, to detect a difference between intervention arms in the mean change from baseline in PPD of 0.25 mm (pooled standard deviation of 0.56 mm, 90% power, 5% significance level, two-sided test) 107 participants will be required per arm, 214 in total [12].

Assessing the proportion of healthy or diseased sites may be a more relevant measure of clinical significance. A PPD of 4 mm is often used as a threshold as pockets ≤4 mm are manageable by the clinician and patient, and pockets ≥5 mm have a poorer long-term prognosis [13, 14]. Proportionally large differences between groups, of 25-50%, are probably required for clinical significance [10]. The pooled standard deviation of the change in the percentage of diseased sites (PPD ≥5 mm) from the pilot trial was 13.5%, and the observed mean reduction from baseline in the control group was 19.0% (which is in line with other studies [15]). To detect a relative 25% difference in the mean reduction in the percentage of diseased sites, 19% versus 23.75% (pooled standard deviation 13.5%, 90% power, 5% significance level, two-sided test) 171 participants will be required per arm, 342 in total.

Both of the sample sizes calculated for these periodontal outcome measures (mean PPD and percentage of diseased sites) are less than the sample size calculated for smoking abstinence (n=674 in total). Taking into account the participant retention rate observed in the pilot trial (73%, 95% CI: 62%-81%), PDD data at 6 months would be expected to be available for approximately 492 participants. Using the same standard deviations as in the PDD sample size calculations above, it can be calculated that, a 0.16 mm difference between intervention arms in the mean change in PPD and a 21% relative difference in percentage of diseases sites would be detectable with PDD data on 246 participants per arm.

The sample size calculations were performed using the *proc power twosamplemeans and twosamplefreq* procedures in SAS version 9.4 of the SAS System for Windows 7, copyright © 2012 SAS Institute Inc.

**Pooled standard deviation for periodontal outcome measures**

| **Parameter** | **Pooled standard deviation** |
| --- | --- |
| 3 month change in mean PPD | 0.51 |
| 6 month change in mean PPD | 0.55 |
| 3 month change in mean PPD [mm] of those sites with a baseline PPD ≥5 mm | 0.65 |
| 6 month change in mean PPD [mm] of those sites with a baseline PPD ≥5 mm | 0.65 |
| 3 month change in mean PPD [mm] of those sites with a baseline PPD ≥6 mm | 1.00 |
| 6 month change in mean PPD [mm] of those sites with a baseline PPD ≥6 mm | 0.96 |
| 3 month change in mean PPD [mm] of those sites with a baseline PPD ≥7 mm | 1.20 |
| 6 month change in mean PPD [mm] of those sites with a baseline PPD ≥7 mm | 1.36 |
| 3 month change in mean PPD [mm] of those sites with a baseline PPD ≤4 mm | 0.30 |
| 6 month change in mean PPD [mm] of those sites with a baseline PPD ≤4 mm | 0.35 |
| 3 month change in percentage of sites with PPD ≥5 mm | 13.96 |
| 6 month change in percentage of sites with PPD ≥5 mm | 13.51 |
| 3 month change in percentage of sites with PPD >6 mm | 7.70 |
| 6 month change in percentage of sites with PPD >6 mm | 7.46 |
| 3 month change in mean MGI | 0.35 |
| 6 month change in mean MGI | 0.50 |
| 3 month change in mean PI | 0.45 |
| 6 month change in mean PI | 0.45 |
| 3 month change in meal CAL | 0.70 |
| 6 month change in mean CAL | 0.81 |
| 3 month change in % BOP score | 12.67 |
| 6 month change in % BOP score | 13.55 |
| 3 month change in CODS | 1.80 |
| 6 month change in CODS | 1.46 |
| 3 month change in PESA [mm^2^] | 383.04 |
| 6 month change in PESA [mm^2^] | 396.23 |
| 3 month change in PISA [mm^2^] | 468.09 |
| 6 month change in PISA [mm^2^] | 461.00 |
| 6 month change in OHQoL-UK | 14.29 |

1. Carr AB, Ebbert J. Interventions for tobacco cessation in the dental setting. The Cochrane database of systematic reviews. 2012:Cd005084.

2. Bullen C, Howe C, Laugesen M, McRobbie H, Parag V, Williman J, et al. Electronic cigarettes for smoking cessation: a randomised controlled trial. Lancet. 2013;382:1629-37.

3. Caponnetto P, Campagna D, Cibella F, Morjaria JB, Caruso M, Russo C, et al. EffiCiency and Safety of an eLectronic cigAreTte (ECLAT) as tobacco cigarettes substitute: a prospective 12-month randomized control design study. PLoS One. 2013;8:e66317.

4. Hajek P, Myers-Smith K, Dawkins L, Goniewicz M, Knight-West O, McRobbie H, et al. The efficacy of e-cigarettes compared with nicotine replacement therapy, when used within the UK stop smoking service. 2015. <http://www.isrctn.com/ISRCTN60477608>. Accessed 13/12/2018.

5. Stapleton J, West R, Hajek P, Wheeler J, Vangeli E, Abdi Z, et al. Randomized trial of nicotine replacement therapy (NRT), bupropion and NRT plus bupropion for smoking cessation: effectiveness in clinical practice. Addiction. 2013;108:2193-201.

6. Sarkar BK, West R, Arora M, Ahluwalia JS, Reddy KS, Shahab L. Effectiveness of a brief community outreach tobacco cessation intervention in India: a cluster-randomised controlled trial (the BABEX Trial). Thorax. 2017;72:167-73.

7. West R, May S, McEwen A, McRobbie H, Hajek P, Vangeli E. A randomised trial of glucose tablets to aid smoking cessation. Psychopharmacology (Berl). 2010;207:631-5.

8. Lindson-Hawley N, Coleman T, Docherty G, Hajek P, Lewis S, Lycett D, et al. Nicotine patch preloading for smoking cessation (the preloading trial): study protocol for a randomized controlled trial. Trials. 2014;15:296-.

9. Dobson AJ, Gebski VJ. Sample Sizes for Comparing Two Independent Proportions Using the Continuity-Corrected Arc Sine Transformation. Journal of the Royal Statistical Society Series D (The Statistician). 1986;35:51-3.

10. Addy M, Newcombe R. Statistical versus clinical significance in periodontal research and practice. Periodontol 2000. 2005;39:132-44.

11. Preshaw PM, Hefti AF, Novak MJ, Michalowicz BS, Pihlstrom BL, Schoor R, et al. Subantimicrobial dose doxycycline enhances the efficacy of scaling and root planing in chronic periodontitis: a multicenter trial. J Periodontol. 2004;75:1068-76.

12. Julious S. Sample sizes for clinical trials. New York: Chapman and Hall; 2010. p. 45.

13. Lang NP, Tonetti MS. Periodontal risk assessment (PRA) for patients in supportive periodontal therapy (SPT). Oral health & preventive dentistry. 2003;1:7-16.

14. Matuliene G, Pjetursson Bjarni E, Salvi Giovanni E, Schmidlin K, Brägger U, Zwahlen M, et al. Influence of residual pockets on progression of periodontitis and tooth loss: Results after 11 years of maintenance. J Clin Periodontol. 2008;35:685-95.

15. Preshaw PM, Holliday R, Law H, Heasman PA. Outcomes of non-surgical periodontal treatment by dental hygienists in training: impact of site- and patient-level factors. Int J Dent Hyg. 2013;11:273-9.
